# Bifurcation of Excited-State Population Leads to Anti-Kasha Luminescence in a Disulfide-Decorated Organometallic Rhenium Photosensitizer

**DOI:** 10.1021/jacs.4c00548

**Published:** 2024-04-10

**Authors:** Julia Franz, Manuel Oelschlegel, J. Patrick Zobel, Shao-An Hua, Jan-Hendrik Borter, Lucius Schmid, Giacomo Morselli, Oliver S. Wenger, Dirk Schwarzer, Franc Meyer, Leticia González

**Affiliations:** † Institute of Theoretical Chemistry, University of Vienna, Währinger Straße 17, A-1090 Vienna, Austria; ‡ Institute of Inorganic Chemistry, University of Göttingen, Tammannstraße 4, D-37077 Göttingen, Germany; § Department of Dynamics at Surfaces, Max-Planck-Institute for Multidisciplinary Sciences, Am Faßberg 11, D-37077 Göttingen, Germany; ∥ International Center for Advanced Studies of Energy Conversion (ICASEC), D-37077 Göttingen, Germany; ⊥ Department of Chemistry, University of Basel, St.-Johanns-Ring 19, CH-4056 Basel, Switzerland; # Vienna Research Platform for Accelerating Photoreaction Discovery, University of Vienna, Währinger Straße 17, A-1090 Vienna, Austria

## Abstract

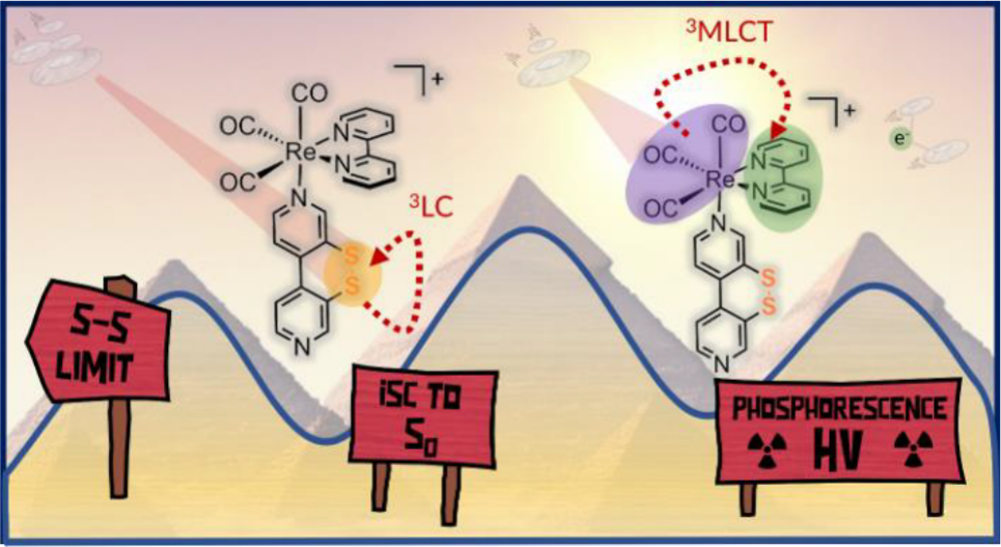

We report a rhenium diimine photosensitizer equipped with a peripheral disulfide unit on one of the bipyridine ligands, [Re(CO)_3_(bpy)(^S–S^bpy^4,4^)]^+^ (**1**^+^, bpy = 2,2′-bipyridine, ^S–S^bpy^4,4^ = [1,2]dithiino[3,4-*c*:6,5-*c*′]dipyridine), showing anti-Kasha luminescence. Steady-state and ultrafast time-resolved spectroscopies complemented by nonadiabatic dynamics simulations are used to disclose its excited-state dynamics. The calculations show that after intersystem crossing the complex evolves to two different triplet minima: a (^S–S^bpy^4,4^)-ligand-centered excited state (^3^LC) lying at lower energy and a metal-to-(bpy)-ligand charge transfer (^3^MLCT) state at higher energy, with relative yields of 90% and 10%, respectively. The ^3^LC state involves local excitation of the disulfide group into the antibonding σ* orbital, leading to significant elongation of the S–S bond. Intriguingly, it is the higher-lying ^3^MLCT state, which is assigned to display luminescence with a lifetime of 270 ns: a signature of anti-Kasha behavior. This assignment is consistent with an energy barrier ≥ 0.6 eV or negligible electronic coupling, preventing reaction toward the ^3^LC state after the population is trapped in the ^3^MLCT state. This study represents a striking example on how elusive excited-state dynamics of transition-metal photosensitizers can be deciphered by synergistic experiments and state-of-the-art calculations. Disulfide functionalization lays the foundation of a new design strategy toward harnessing excess energy in a system for possible bimolecular electron or energy transfer reactivity.

## Introduction

**Figure 1:**
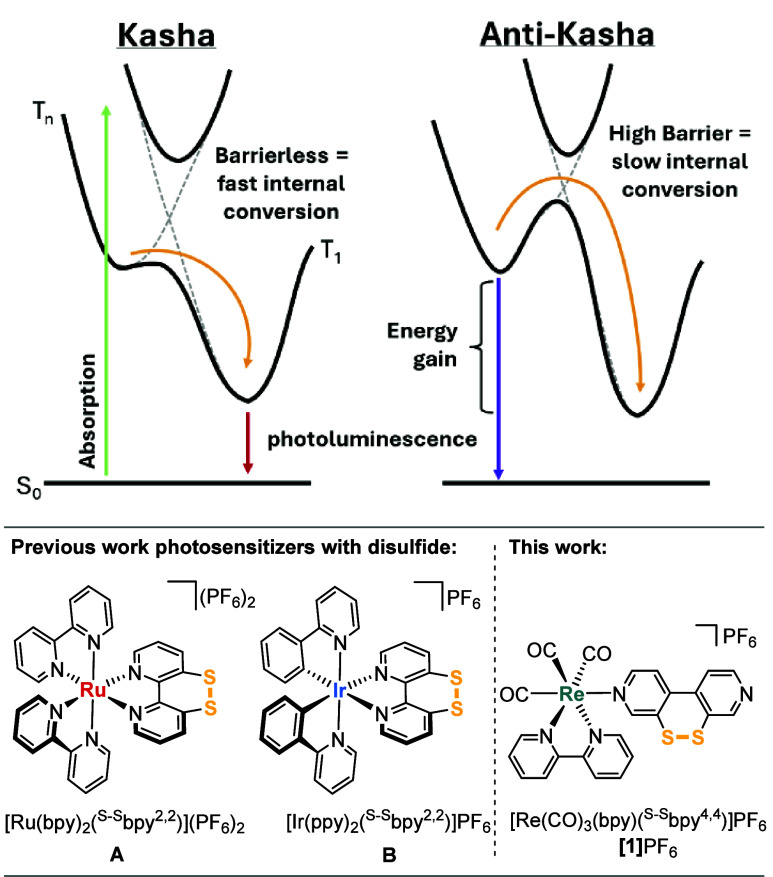
Top: Energy diagrams of Kasha (left) and anti-Kasha behavior (right) due to slow internal conversion (IC) between higher- and lower-energy states (adapted from ref (7)). Bottom: Structures of complexes with sulfurated ligand ^S–S^bpy^2,2^**A**,^16,17^**B**,^18^ and complex **[1]**PF_6_ investigated herein.

Substantial efforts are currently dedicated to meet global energy demands by exploiting sunlight. To this aim, efficient materials for solar energy conversion and solar fuel generation are needed.^1−4^ The ultimate example for an efficient solar energy conversion scheme is plant growth. In natural photosynthesis, sunlight is absorbed across the visible spectrum,^5^ yet the energy of blue photons absorbed by chlorophyll is partly lost due to rapid relaxation from an initially populated higher-energy state to the photochemically active, lowest-energy state via internal conversion (IC).^6^ Triggering photochemical reactions with these higher-energy excited states would greatly improve photonic efficiency and therefore minimize losses,^7,8^ in turn making energetically more demanding reactions possible. In the pursuit of finding new and efficient ways for solar energy conversion,^9,10^ recent years have seen increasing interest in investigating reactivity from higher-lying excited states.^8^ To date, only a few systems can leverage higher-lying states to initiate photochemistry^8,11,12^ because a fundamental statement is prohibiting access: Kasha’s rule states that relaxation to the lowest-energy state of a given multiplicity is ultrafast, and therefore, all photochemistry occurs from this state.^13−15^ In this usual case, population from higher-energy states decays rapidly via IC to the lowest-energy state due to strong electronic coupling, resulting in a low barrier between the two states of interest (Figure 1, top left).^8^ Weakening electronic coupling and raising this barrier (Figure 1, top right) leads to a deceleration of the IC rate so that photon emission or bimolecular reactivity (requiring ∼ns lifetimes) from that state can compete. Hence, finding ways to slow down IC and making a higher-energy state photochemically active can open new avenues for exploiting excited-state reactivity, including artificial photosynthesis,^7,19^ optoelectronics, photovoltaic devices,^20,21^ and molecular photoswitches.^8,22^

Most systems known to exhibit anti-Kasha behavior are organic chromophores, such as azulene, while transition-metal complexes remain underexplored. Previously studied systems include porphyrins,^23,24^ as well as ruthenium or molybdenum polypyridine systems.^7,12,19,25,26^ Despite recent advances,^11,25,27,28^ there is a growing need to explore relaxation dynamics initiated by higher excited states, also with computational protocols.^28^ Understanding and controlling the many photophysical phenomena that underpin vital natural processes hold the key to developing novel technologies.^29−31^ While spectroscopy usually provides only a global response of the system to light, computer simulations help disentangle the multiple underlying spectroscopic signatures. However, such simulations still require approximations to make them computationally affordable, introducing inherent methodological errors. In this light, there is a natural need for synergy between spectroscopic experiments and computational simulations, where the strengths of each side are leveraged to gain a better description of the system.

Recently, we investigated the excited-state dynamics of disulfide-functionalized complexes [Ru(bpy)_2_(^S–S^bpy^2,2^)]^2+^ (**A**; bpy = 2,2′-bipyridine)^16,17^ and [Ir(ppy)_2_(^S–S^bpy^2,2^)]^+^ (**B**; Hppy = 2-phenylpyridine)^18^ containing the sulfurated 2,2′-bipyridine derivative ^S–S^bpy^2,2^ (Figure 1, bottom left).^32^ The electron-accepting disulfide moiety was able to fully direct charge flow in the excited state toward the ^S–S^bpy^2,2^ ligand, where a direct response via S–S bond elongation was revealed by computational simulations, indicating that the excited electron populates the antibonding σ* orbital of the S–S bond.^17^ The disulfide-centered excited state is generally lower in energy compared to the parent complexes. However, we noticed that in the ground state, the disulfide unit does not interact strongly with the complex framework,^16,18,33^ and thus substitution with the disulfide does not significantly influence the overall electronics of the system, in contrast to functional groups with charges, inductive effects, or extended aromatic systems.^34^ Vlček and co-workers for example showed that a positively charged methylviologen ligand partially directs charge flow toward it.^35^ We thus hypothesized that spatial decoupling of a chromophoric photosensitizing unit and the disulfide electron accepting unit might also lead to the desired electronic decoupling necessary for deceleration of the IC.^36^ Previous works from T. J. Meyer^37−40^ and Vlček within the last decades focused on the electronic interactions of different ligands with rhenium diimine chromophores^41−46^ and the resulting effects on the time evolution of vibrational and optical spectra. However, the observation of anti-Kasha photoluminescence resulting from suppressed and incomplete IC has never been reported.

Herein, we report a novel rhenium(I) complex, *fac*-[Re(CO)_3_(bpy)(^S–S^bpy^4,4^)]PF_6_ ([**1**]PF_6_, featuring the ligand [1,2]dithiino[3,4-*c*:6,5-*c*′]dipyridine (^S–S^bpy^4,4^) in the axial position of the {(CO)_3_Re(bpy)} chromophore (**C**; Figure 1, bottom right)), that shows anti-Kasha photoluminescence. The impact of the peripheral disulfide moiety on the charge flow, time scales, and evolution of the excited-state dynamics is unraveled by ultrafast transient infrared spectroscopy (TRIR), complemented by nonadiabatic dynamics simulations. Steady-state emission spectroscopy reveals the fate of the complex after the ultrafast processes. Rhenium diimine complexes typically emit through a ^3^MLCT state originating from a transition from the rhenium metal center to the equatorial bpy-type ligand^47,41,45,48^ and feature long-lived excited states and easy tunability.^49−52^ Further, the intense carbonyl stretching vibration is highly sensitive to the electronic environment, making time-resolved infrared spectroscopy an excellent tool to study its dynamics.^42,45,53−55^

## Results

### Synthesis and Characterization of the Ligand and Complex

**Scheme 1:**
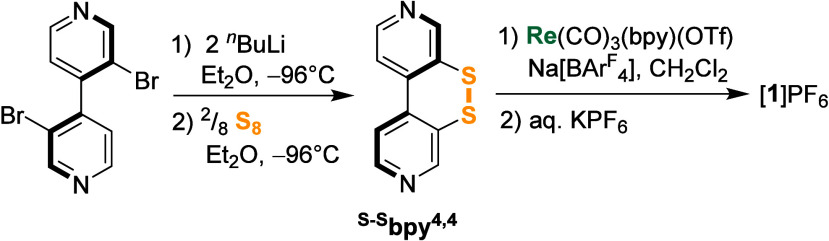
Synthesis of the Ligand ^S–S^bpy^4,4^ and Complex [**1**]PF_6_

The ligand ^S–S^bpy^4,4^ has previously been synthesized via a multistep procedure starting from pyridin-3-ol in an overall yield of 14%.^56−58^ We now developed a convenient two-step protocol, starting with the homocoupling of 3-bromopyridine to form 2,2′-dibromo-4,4′-bipyridine, based on a slightly modified procedure from Baumgartner et al.^59^ From here, the disulfide is incorporated by 2-fold bromide–lithium exchange with ^*n*^BuLi in diethyl ether at −94 °C followed by quenching of the lithiated species with elemental sulfur, as shown in Scheme 1 (see Experimental Section for details).

After workup, ^S–S^bpy^4,4^ was obtained as a yellow solid with a yield of 18%. The overall yield starting from 3-bromopyridine is comparable with the literature procedure, but the number of steps is dramatically reduced from eight to two. The rhenium(I) complex [**1**]PF_6_ was then prepared by reacting the precursor complex Re(CO)_3_(bpy)(OTf) (OTf = triflate, SO_3_CF_3_) with an excess of ^S–S^bpy^4,4^ and the additive Na[BAr^F^_4_] ([BAr^F^_4_]^−^ = tetrakis(3,5-bis(trifluoromethyl)phenyl)borate) in dichloromethane.^60^ The addition of Na[BAr^F^_4_] to abstract the triflate and the use of an excess of ^S–S^bpy^4,4^ allow for a rapid ligand exchange and formation of the target complex [Re(CO)_3_(bpy)(^S–S^bpy^4,4^)]BAr^F^_4_ ([**1**]BAr^F^_4_) as the major product. As a minor product (∼1:50), the bimetallic ^S–S^bpy^4,4^-bridged complex [Re(CO)_3_(bpy)–(^S–S^bpy^4,4^)–Re(CO)_3_(bpy)]^2+^ was identified, but not isolated. After chromatographic purification, anion exchange with aqueous KPF_6_ and crystallization from dichloromethane/hexanes, the final product [**1**]PF_6_ was obtained as an analytically pure yellow solid in 78% yield. [**1**]PF_6_ was fully characterized, including mass spectrometry and elemental analysis (see Experimental Section).

**Figure 2:**
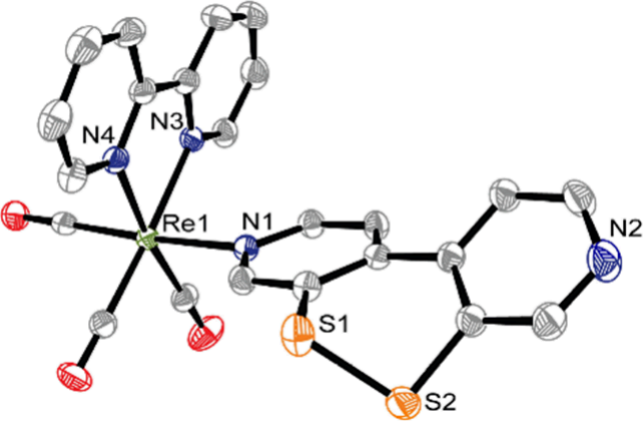
ORTEP drawing of **1**^**+**^ (thermal ellipsoids at 50% probability level); hydrogen atoms, counterions, and solvent molecules are omitted for clarity. Selected bond lengths [Å]: Re1–C21 1.932(3), Re1–C22 1.918(3), Re1–C23 1.925(3), Re1–N1 2.214(2), Re1–N3 2.171(2), Re1–N4 2.164(2), S1–S2 2.059(2).

[**1**]PF_6_ crystallizes in the monoclinic space group *P*2_1_/*n* with one molecule of CH_2_Cl_2_ in the unit cell. The central Re ion is coordinated octahedrally by three CO molecules in *fac*-arrangement, the equatorial 2,2′-bipyridine, and the ligand ^S–S^bpy^4,4^ (Figure 2). The S–S bond length is 2.059 Å, and the C–S–S–C torsion angle is 60°; both values are similar to the ones observed for **A** (2.045 Å and 57°).^33^ However, the torsion angle between the two pyridine rings of monodentate ^S–S^bpy^4,4^ in [**1**]PF_6_ (35°) is much larger than of the *N*,*N*′-chelating ^S–S^bpy^2,2^ ligand in **A** (18°), indicating a higher degree of flexibility/rotational freedom compared to the ^S–S^bpy^2,2^ ligand in complexes **A** and **B**.

The IR spectrum of [**1**]PF_6_ in a KBr matrix (see Section S1.1 in the Supporting Information) shows three intense bands in the CO stretching region at 2033, 1934, and 1912 cm^–1^. In tetrahydrofuran (THF) solution, the high-energy band shifts to 2035 cm^–1^ and the two low-energy bands merge to form one broad band around 1930 cm^–1^ (see Figure 4a). Such quasi-degeneracy of the two low-energy bands in solution has been reported for axially *N*-coordinated Re complexes.^61^ Voigt profile fitting (Section S1.1) was applied to determine the center frequencies of the two low-energy modes to be 1925 and 1936 cm^–1^ in THF solution. The high-energy band originates from an A′(1) totally symmetric in-phase stretching vibration of all three COs, while the A′(2) is the out-of-phase vibration. The low-energy band has A″ symmetry and involves the antisymmetric stretching vibration of the equatorial COs.^45^ NMR analysis (Section S1.1) shows that the complex is *C*_*s*_-symmetric in THF solution, indicating that the toggling of the disulfide unit is fast on the NMR time scale. Note that THF was chosen as a solvent for all investigations because the complex was found to gradually photodissociate in acetonitrile solution into [Re(bpy)(CO)_3_(NCCH_3_)]^+^ and free ^S–S^bpy^4,4^.

**Figure 3:**
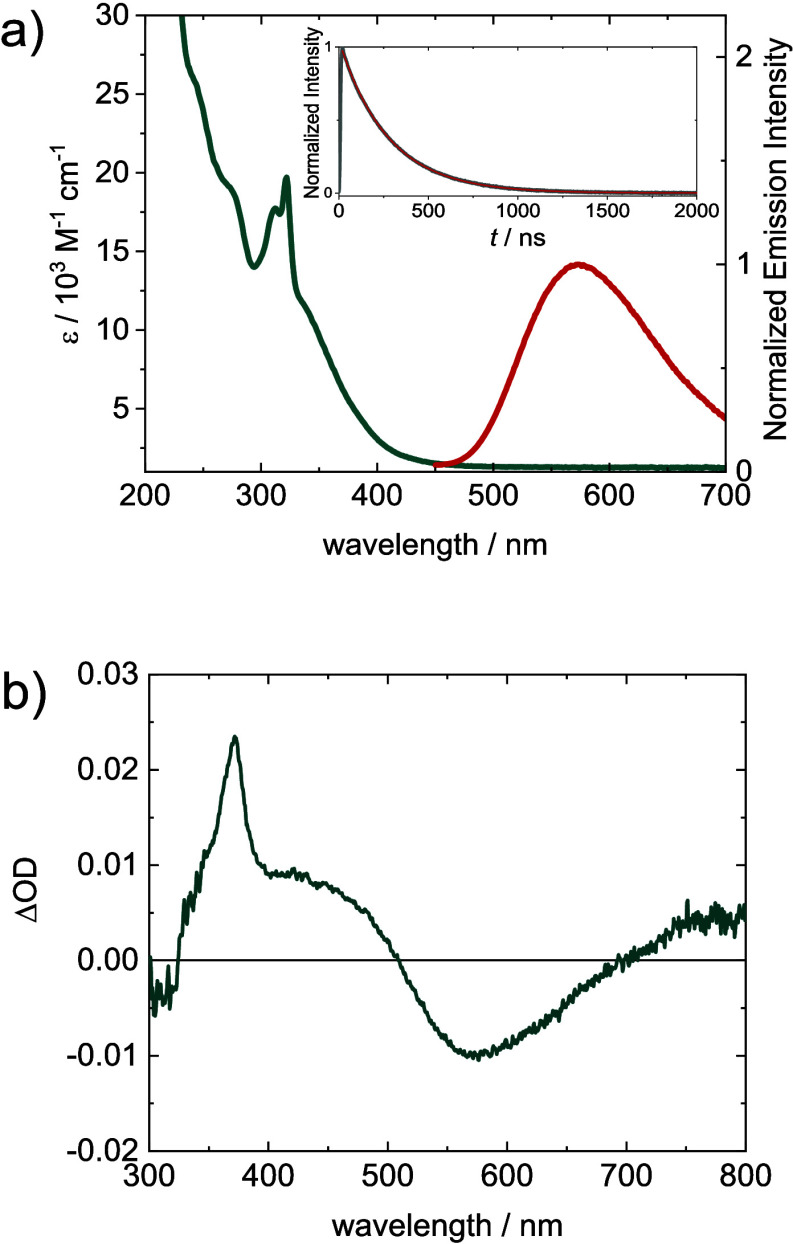
(a) UV–vis absorption (green line) and emission spectra (red line, excitation at 370 nm) of [**1**]PF_6_ (25 μM) in deaerated THF. Inset: Time-resolved emission decay at 573 nm (excitation at 355 nm), with monoexponential fit (270 ns). (b) Transient absorption spectrum in deaerated THF measured in a time window of 20 ns after excitation at 355 nm.

**Figure 4:**
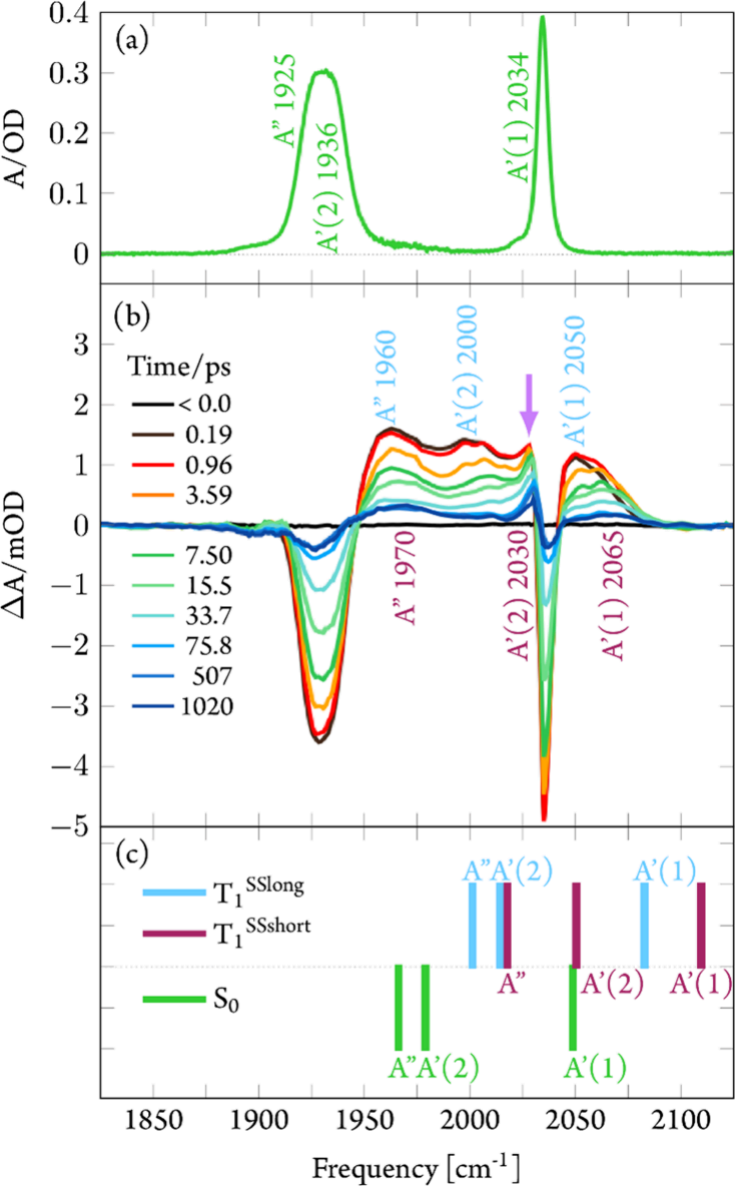
(a) FTIR spectrum of [**1**]PF_6_ in THF. (b) Transient difference spectra of [**1**]PF_6_ in THF recorded after 400 nm excitation at time delays as indicated. The purple arrow at the 2030 cm^–1^ peak is a guide for the eye; see below for explanation. (c) Calculated CO stretching frequencies for the ground-state S_0_ and the triplet minima T_1_^SSlong^ (of ^3^LC character, see below) and T_1_^SSshort^ (of ^3^MLCT character, see below). See Discussion for details.

### Luminescence Properties

The absorption spectrum of [**1**]PF_6_ in THF (Figure 3a, green line) shows the typical features of a rhenium diimine complex, with a sharp peak at 322 nm and a broad shoulder toward lower energies. The low-energy shoulder is typically assigned to the MLCT excitation from the rhenium center to the equatorial bpy ligand. After absorption at 370 nm (3.35 eV), [**1**]PF_6_ exhibits luminescence centered at 573 nm (2.16 eV) with a lifetime of τ = 270 ns (Figure 3a, red line and inset). This emission corresponds to phosphorescence, as the presence of the heavy rhenium atom allows for efficient intersystem crossing (ISC). The broad and structureless, long-lived emission of rhenium carbonyl diimine complexes is typically assigned to originate from a ^3^MLCT (d_π_π*_bpy_) state.^61,62^ In the transient absorption spectrum (Figure 3b) recorded 20 ns after UV excitation, an excited-state absorption (ESA) in the UV region with a sharp peak at 372 nm appears, indicative of formation of the equatorial bpy^•–^ radical anion as a result of MLCT excitation.^39,63^ The ESA extends up until 500 nm. At 573 nm, spontaneous emission is observed, which corresponds nicely to the observed steady-state emission band. When traced kinetically, both the 372 and the 573 nm components decay with similar lifetimes (τ = 274 ± 1 ns), also in excellent agreement with the one measured via luminescence spectroscopy (Figure 3a, inset).

The absorption/emission features are similar to the ones of unsulfurated [Re(CO)_3_(bpy)(4,4′-bpy)]PF_6_,^39,64^ suggesting that sulfuration does not change the main electronics of the system. However, the photoluminescence quantum yield in deaerated THF of ϕ_total_ = 0.3 ± 0.1% is about 1 order of magnitude smaller than for similar rhenium diimine complexes reported in the literature (cf. 3% in [Re(CO)_3_(bpy)(py)]PF_6_, py = pyridine).^39,64−66^ Interestingly, the radiative lifetime is comparable to typical Re diimine complexes emitting through an ^3^MLCT state, which seems surprising, as both values are connected through the radiative rate constant *k*_r_ (eq 1). This implies the existence of an additional dark process that reduces the photoluminescence quantum yield according to
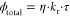
1where η is the efficiency of populating the emissive ^3^MLCT state, which in this case should be around 10%. Hence, we conducted ultrafast transient absorption spectroscopy to investigate this observation in closer detail.

### Transient Absorption Spectroscopy

The excited-state dynamics of [**1**]PF_6_ in THF were investigated using fs pump–probe absorption spectroscopy. Transient difference spectra in the visible (400–730 nm) and in the mid-IR region of the carbonyl stretching vibrations (1700–2100 cm^–1^) were measured upon 400 or 266 nm excitation with about 100 fs time resolution (Section S1.2).

The transient IR spectra obtained following 400 nm excitation (Figure 4b) show instantaneous bleaching of the ground-state CO stretching vibrations and the appearance of broad blue-shifted bands with maxima at approximately 1960, 2000, and 2050 cm^–1^.

**Figure 5:**
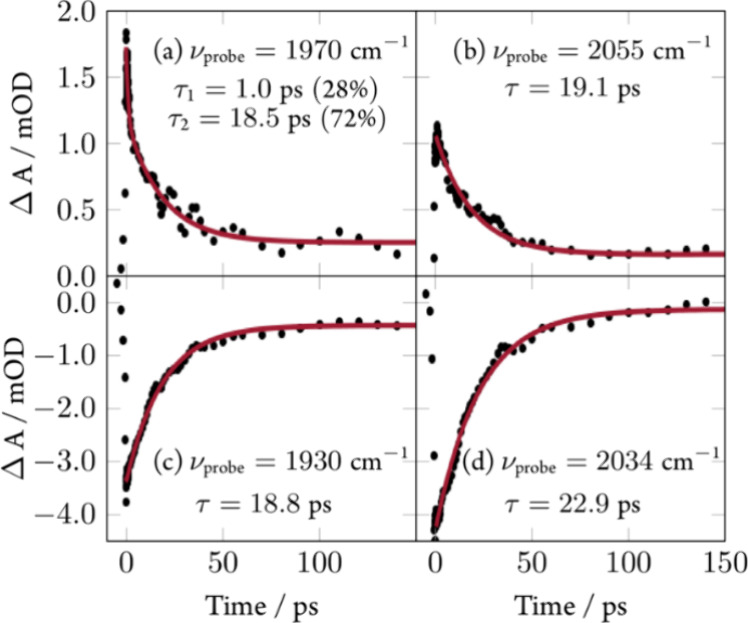
Time traces for the data of Figure 4b at selected probe frequencies with exponential fits.

Within several tens of ps these bands decay toward a constant absorption level whereby the 2000 and 2050 cm^–1^ features apparently undergo a blueshift. Concomitantly, the bleached ground-state bands recover by ∼90%, indicating the formation of a long-lived product. Analyzing these processes quantitatively by fitting exponential decays to time traces for several selected probe frequencies (Figure 5) reveals one dominant average time constant of τ_1_ = 20 ± 3 ps being responsible for excited-state decay and ground-state recovery. At probe frequency ν_probe_ = 1970 cm^–1^ there is also evidence for another time constant of τ_2_ = 1.0 ± 0.3 ps involved in the excited-state dynamics.

The photoproduct spectrum at pump–probe delays > 100 ps (Figure 4b, blue lines) is characterized by two broad absorption bands at 1970 and 2065 cm^–1^ and a narrower peak at 2030 cm^–1^. A closer inspection shows that this peak is visible already in the earliest spectra (purple arrow in Figure 4b), indicating that the long-lived photoproduct is formed on a sub-ps time scale. We observe no evidence for a strong pump wavelength dependence of the excited-state dynamics. Transient IR spectra measured after 266 nm excitation show essentially the same features as for 400 nm excitation (Section S1.2). The major difference is the apparently delayed emergence of the 2030 cm^–1^ peak. We attribute this to a large increase of the vibrational excess energy in the excited states (due to the higher applied photon energy at 266 nm), which broadens all the vibrational transitions and reveals the 2030 cm^–1^ band only when the molecule has cooled down. A key finding of Figure 4b is however reproduced, namely, that the initial ground-state bleach recovers by 90% within the first 100 ps, indicating that the yield of the long-lived product (∼10%) is independent of excitation energy.

The fs transient UV–vis absorption spectra are consistent with the IR data. After 340 nm excitation, enhanced absorption over the whole visible spectral range is observed, which decays to a constant offset spectrum within 80 ps (see Figure S8 in Section S1.2). Already the earliest transients show a peak at 375 nm persisting over the whole time window, in agreement with the 20 ns spectrum of Figure 3b, indicating instant formation of the long-lived bpy^•–^ radical anion of the ^3^MLCT state. The decay toward the photoproduct spectrum is characterized by two time constants: τ_1_ = 11 ± 2 ps and τ_2_ = 0.6 ± 0.2 ps (see Figure S8).

### Experimental and Calculated Absorption Spectra

**Figure 6:**
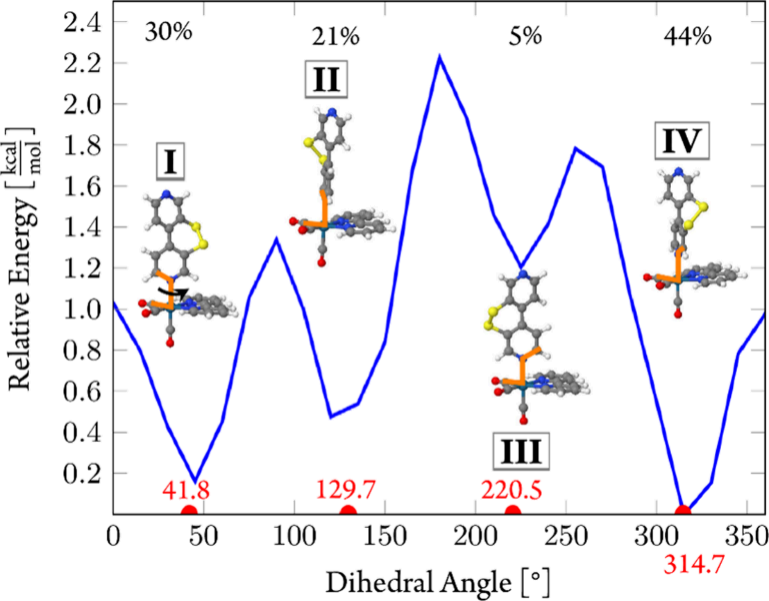
Relaxed scan around the Re–N bond of the axial ligand (indicated by the arrow at structure **I**) of **1^+^** at the PBE0/TZVP level of theory in the gas phase. The scanned dihedral angle is indicated in orange in the molecular structures. Percentages refer to Boltzmann populations at *T* = 300 K.

As a next step, we calculate the absorption spectrum and compare it with the experimental one. Corresponding computational details are reported in Section S2.1. We start by considering the role of the rotation around the Re–N bond of an axial N-donor ligand, which has been discussed for other rhenium carbonyl diimine complexes.^67^ In the case of **1**^**+**^ with ^S–S^bpy^4,4^ as the axial ligand, a relaxed scan around the Re–N bond shows four minima (**I**–**IV**), all displaced by consecutive rotation of ca. 90° (Figure 6).

The lowest energy conformer **IV** resembles the geometry observed crystallographically for [**1**]PF_6_, as shown in Figure 2. However, as all local minima are separated by small barriers of only 1–2 kcal mol^–1^, all four conformers should be accessible in solution at room temperature and, thus, should be considered in the calculation of the absorption spectrum.

We calculate the absorption spectrum in THF up to energies of ca. 4.5 eV (275 nm) with time-dependent density functional theory (TDDFT) and a triple-ζ basis set (TZVP). Six different density functionals were tested (Section S2.2), from which the PBE0 was selected as best performer and used for further calculations. In order to include the vibrational effects of the molecule moving in its electronic ground state, Wigner sampling at 300 K was performed.^68,69^ The number of geometries taken in each of the four ensembles was chosen according to the Boltzmann population at *T* = 300 K, totaling 550 distinct geometries.

**Figure 7:**
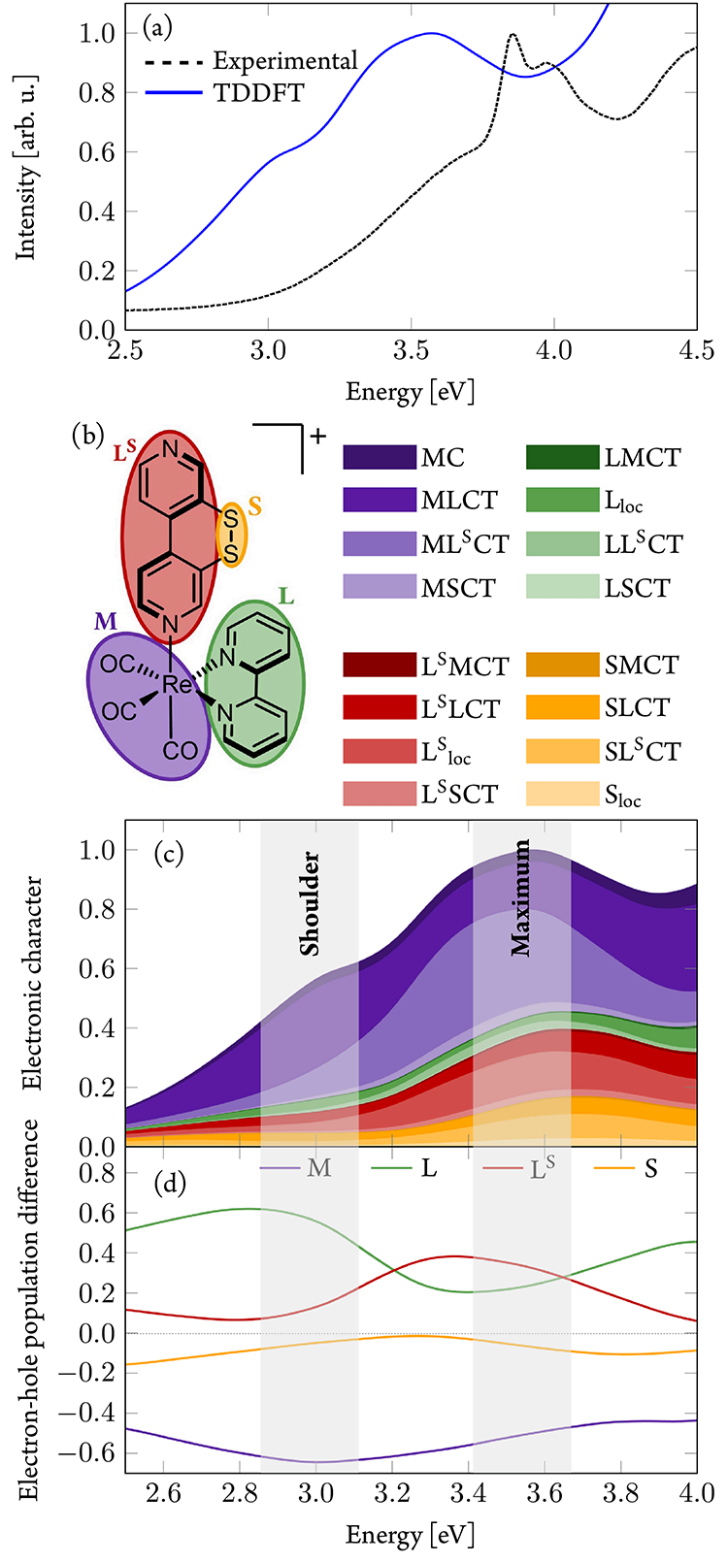
(a) Comparison of experimental absorption spectrum of [**1**]PF_6_ in THF solution (black, dotted line) and calculated absorption spectrum of **1**^+^ (PBE0/TZVP; blue, solid line) in the gas phase. (b) Fragmentation of the complex used in the transition-density matrix analysis. (c) Electronic character of states contributing to the spectrum. (d) Electron–hole difference population in the spectrum.

Figure 7a shows the resulting calculated absorption spectrum compared to the experimental one. The latter features a split band with two peaks at 3.85 and 3.97 eV, preceded by a shoulder at ca. 3.6 eV. The simulated spectrum displays a single broad maximum at 3.55 eV with a small shoulder at lower energies. Accordingly, PBE0/TZVP underestimates the experimental energies by ca. 0.3–0.5 eV, yet the general shape is reasonably reproduced. The influence of solvent effects and spin–orbit couplings on the absorption spectrum is discussed in Section S2.3. The electronic states contributing to the absorption spectrum are obtained from the analysis of the transition-density matrix between the electronic ground state and the excited states at each geometry.^70^ To this aim, we divided the complex into four fragments (Figure 7b): the Re(CO)_3_ fragment (M), the bpy ligand (L), the S–S bridge (S), and the 4,4′-bipyridyl core of the ^S–S^bpy^4,4^ ligand (L^S^). The metal center and the three CO ligands are combined into one fragment because they behave as one unit across the entire energy range of the spectrum (Section S2.4), as observed before in other rhenium(I) carbonyl diimine complexes.^45,71,72^ In our previous investigations, we found that the disulfide unit is somewhat disconnected from the aromatic system and hence can and should be treated separately. These four fragments allow characterizing the wave functions in terms of linear combination of the 16 different possible excitation types (Figure 7b): local excitations within each fragment (MC, L_loc_, L^S^_loc_, S_loc_) or X → Y charge transfer (CT) excitations XYCT, where an electron is transferred from fragment X to Y, leaving a hole in fragment X when compared to the reference ground state.

Figure 7c illustrates the obtained contributions of the different excitations to the absorption spectrum. In the low-energy range reaching until the shoulder, CT excitations from the Re(CO)_3_ group to the equatorial 2,2′-bpy ligand (MLCT) prevail (purple), while at higher energies around the maximum of the absorption band, CT (light purple) to the ^S–S^bpy^4,4^ ligand dominates (ML^S^CT).

Excitation of rhenium(I) carbonyl diimine complexes typically populates MLCT states involving the equatorial diimine ligand;^45,73,74^ these are the states here denoted as MLCT and represent those found in **1**^**+**^ at energies around the shoulder and below (<3.2 eV). However, also at these energies, there are already substantial contributions of other electronic characters. We observe a mixture of electronic characters present throughout the entire computed spectrum.

The different CT contributions can be summed up to contributions that share the same donor fragment (“hole”) and those that share the same acceptor fragment (“electron”). For each fragment, one can then calculate the electron–hole difference to quantify the amount of charge that is transferred upon excitation; see Figure 7d. As the positive (negative) differences denote larger electron (hole) parts on a fragment, the Re(CO)_3_ fragment (M) has large negative electron–hole difference population at all energies, thus acting as the origin of the charge flow. An additional small negative electron–hole difference is seen at the disulfide unit (S) of the ^S–S^bpy^4,4^ ligand, albeit much less compared to the Re(CO)_3_ fragment. Thus, the presence of the disulfide unit increases slightly the overall CT character of the excitations (as noted in the higher extinction coefficient at the shoulder compared to the spectrum of a reference compound in Figure S12). At lower energies, the charge flow is directed from the metal to the bpy ligand (L), as it is known in the literature. Between energies of 3.2 and 3.7 eV, i.e., around the maximum of the absorption band, charge is predominantly transferred to the aromatic core of the ^S–S^bpy^4,4^ ligand (L^S^). At even higher energies, more charge again flows to the bpy ligand L upon excitation. Only at energies above 4 eV (Figure S11) are ππ* states involving the bpy ligand (green) populated in greater amount.

### Triplet Optimization Starting from the Franck–Condon Geometry

**Figure 8:**
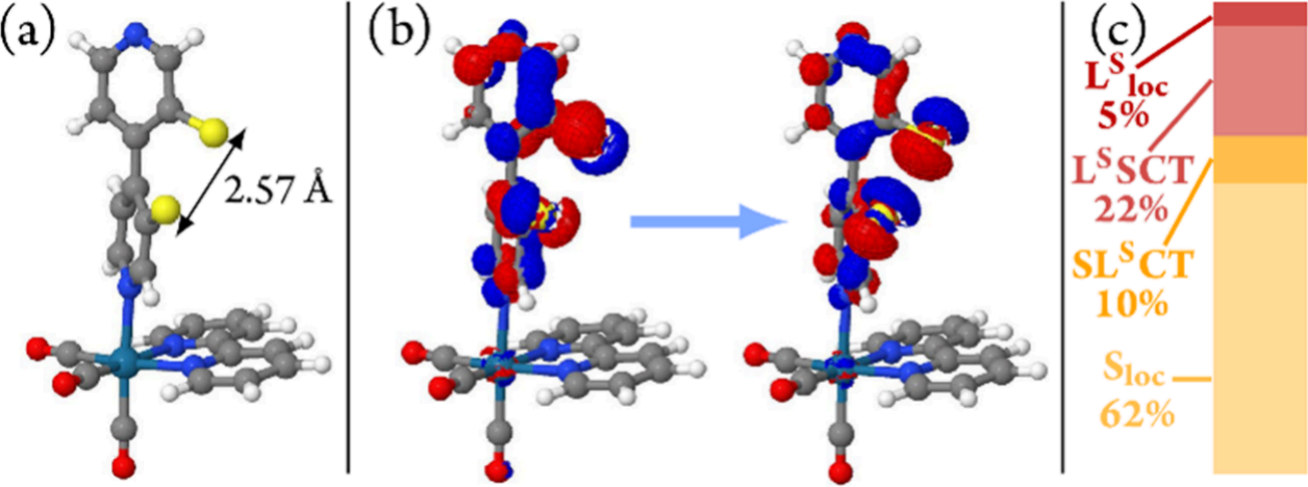
(a) Optimized geometry of the lowest-lying triplet state starting from the Franck–Condon geometry. (b) Natural transition orbitals characterizing the T_1_ state at this geometry, called the T_1_^SSlong^ state. (c) Transition-density matrix analysis of the T_1_^SSlong^ state, showing its predominant ^3^LC character.

In order to investigate the emissive state of **1**^+^ and to unravel the effect of the disulfide unit, we searched for the lowest-energy triplet state (Section S3.1). A geometry optimization starting from the Franck–Condon geometry of either of the four conformers **I**–**IV** leads to similar structures with energies differing by less than 0.03 eV. Depending on the starting conformer, the structures are connected by ca. 90° rotation around the Re–N bond. The structure obtained starting from conformer **IV** (of Figure 6) is shown in Figure 8a. It is characterized by an increased S–S bond length of 2.57 Å compared to the 2.05 Å at the Franck–Condon geometry and 2.06 Å for the crystallographically determined structure of [**1**]PF_6_, which is accompanied by a larger torsion angle between the two pyridine rings of the ^S–S^bpy^4,4^ ligand. This geometric distortion can be understood by the character of the T_1_ state: a ligand-centered (LC) excitation in ^S–S^bpy^4,4^ from the aromatic π and sulfur p orbitals to the σ* orbital of the S–S bond (see the natural transition orbitals of the T_1_ state in Figure 8b). Due to its structural features, we will refer to this state as T_1_^SSlong^. For later reference, Figure 8c shows the composition of this state in terms of electronic excitations and shows that it has predominantly a ^3^LC character. The lowest-energy triplet state at the Franck–Condon geometry possesses the same electronic character. Thus, starting from the Franck–Condon geometry, increasing the S–S bond length stabilizes this T_1_ state by facilitating population of the σ* orbital of the S–S bond.

Increasing the S–S bond length to 2.57 Å stabilizes the T_1_^SSlong^ (^3^LC) state significantly, so that its energy gap to the ground state is only 0.56 eV (2214 nm). However, compared to the experimental emission energy of 2.16 eV (573 nm), there is a difference of 1.60 eV. As the PBE0 functional led to errors of 0.3–0.6 eV in the computed absorption spectrum, in agreement with related cases,^75^ there are two plausible hypotheses to explain this difference: (i) either the PBE0 functional predicts the wrong emissive state or (ii) there exists another triplet state able to account for the experimentally observed phosphorescence.

To test the first hypothesis, we calculated the T_1_ energy at this geometry with other density functionals (Section S3.2, Table S1). The DFT predictions are rather consistent, with a maximum T_1_ excitation energy of 0.90 eV for the long-range corrected functional ωB97X-V, still 1.26 eV apart from the experimental value. We thus conclude that it is unlikely that the large difference between computed and experimental emission energy can be attributed to a failure of DFT. In order to test the second hypothesis, we performed nonadiabatic dynamics simulations hoping they provide information about relevant populated triplet states.

### Short-Time TDDFT Nonadiabatic Dynamics Simulations

To find which electronic states could be responsible for the orange phosphorescence of **1**^**+**^, nonadiabatic trajectory surface hopping simulations based on *on-the-fly* PBE0/TZVP quantum chemistry were performed (henceforth referred to as TDDFT/SH simulations).^76^ The dynamics are started by an excitation in the energetic range of 2.7–3.2 eV, compatible with our experimental excitation wavelength (370 nm, 3.35 eV) as they consider the redshift found in the PBE0-calculated spectrum. Initially, 101 trajectories were propagated during 100 fs including 15 singlet and 15 triplet excited states. Due to statistical anomalies, five trajectories had to be excluded in the analysis (further computational details are in Section S4.1).

**Figure 9:**
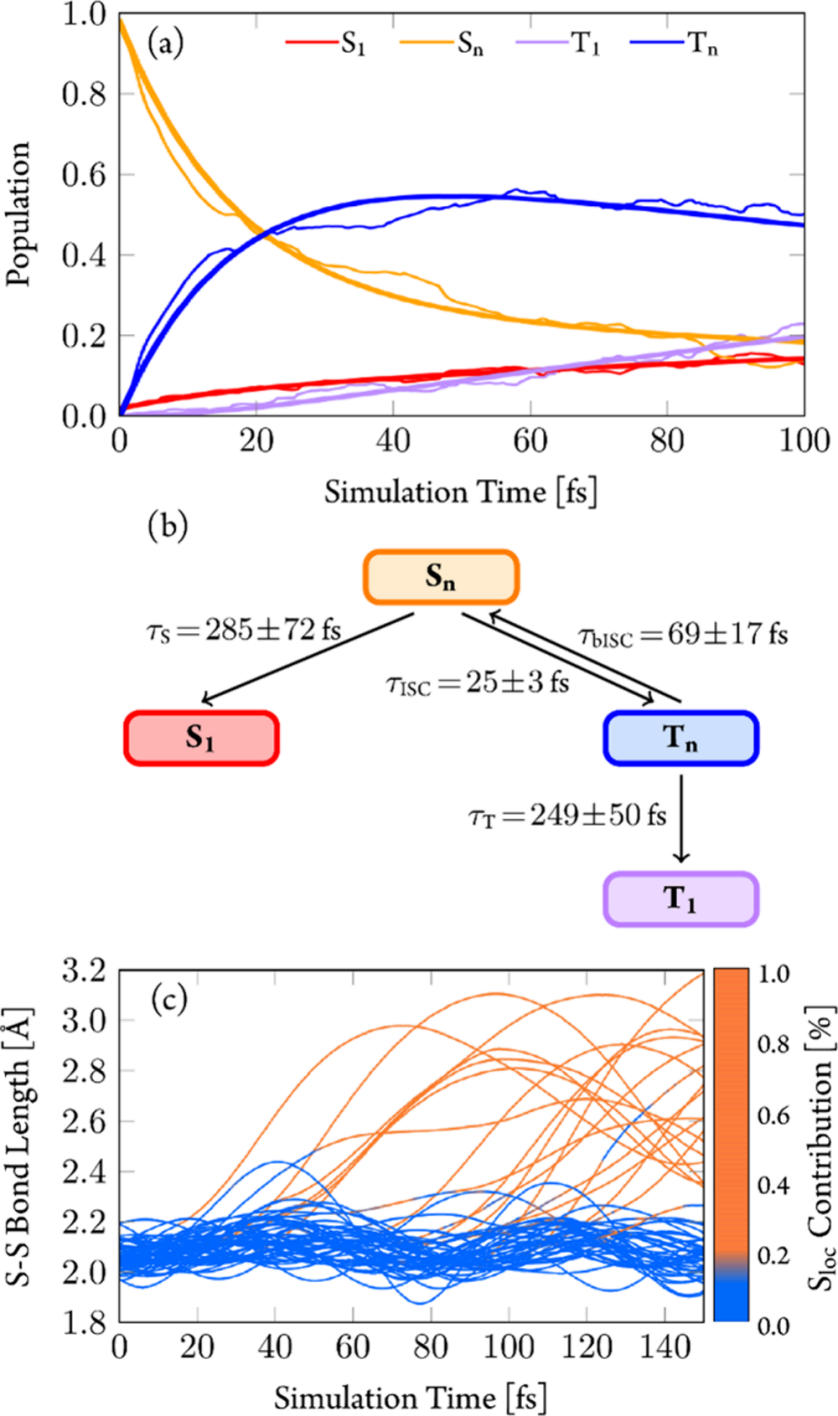
(a) Adiabatic electronic-state populations from TDDFT/SH dynamics. Higher-lying singlet and triplet states combined to one line each. Thick lines represent fitted curves according to the mechanism shown in (b). (c) Time evolution of S–S bond length for individual trajectories. Color coding for the amount of S_loc_ excitation character from the transition-density matrix analysis.

Figure 9a shows the obtained time evolution of the adiabatic electronic state populations (thin lines). After initial excitation to the singlet states, most of the population reaches the T_*n*_ states within 60 fs. After ca. 20 fs, the population of the T_1_ state (purple line) begins to increase, reaching ca. 20% after 100 fs. The dynamics suggest a simple kinetic model in which population is transferred from the S_*n*_ states via internal conversion (IC) to the S_1_ state and, via ISC, to the T_*n*_ states. From the T_*n*_ states, relaxation to the T_1_ state occurs via IC.

The full computed mechanism (Section S4.2) is summarized in Figure 9b, and the resulting exponential fits of the populations are shown by thick lines in Figure 9a. As the fitted curves resemble closely the simulated populations, this simple mechanism seems reasonable. Accordingly, ISC from the S_*n*_ to T_*n*_ occurs with a time constant of τ_ISC_ = 25 ± 3 fs, while IC from the S_*n*_ to the S_1_ state is slower (τ_S_ = 285 ± 72 fs). In the T_*n*_ states, IC to the T_1_ takes place with a time scale of τ_T_ = 249 ± 50 fs, and back-ISC to the singlet states has a time constant of τ_bISC_ = 69 ± 17 fs (see also Section S4.3). According to this mechanism, around 15% population should be trapped in the S_1_ state. The fate of the S_1_ state population will be discussed below.

In terms of nuclear degrees of freedom, an analysis of the trajectories evidenced two different relaxation pathways (Figure 9c) within 150 fs of simulation time. Most trajectories (79 of 96, 82%) stayed around the vicinity of the Franck–Condon geometry (pathway 1, blue curves), while the remaining trajectories (17 of 96, 18%) underwent pronounced elongation of the S–S bond length (pathway 2, orange curves). The different electronic character of both types of trajectories is indicated by the amount of disulfide-directed excitation character (S_loc_). Trajectories involving long S–S bonds (orange curves) do not lead to dissociation of the S–S bond; after reaching maximum values around 3.0 Å, the bond shrinks again. This bond length decrease does not reach the values around 2.1 Å of the trajectories near the Franck–Condon region (blue curves), but rather oscillates around 2.6 Å, the bond length of the T_1_^SSlong^ (^3^LC) geometry (2.57 Å). These oscillations continue (Section S4.4) for two trajectories propagated up to 200 fs.

**Figure 10:**
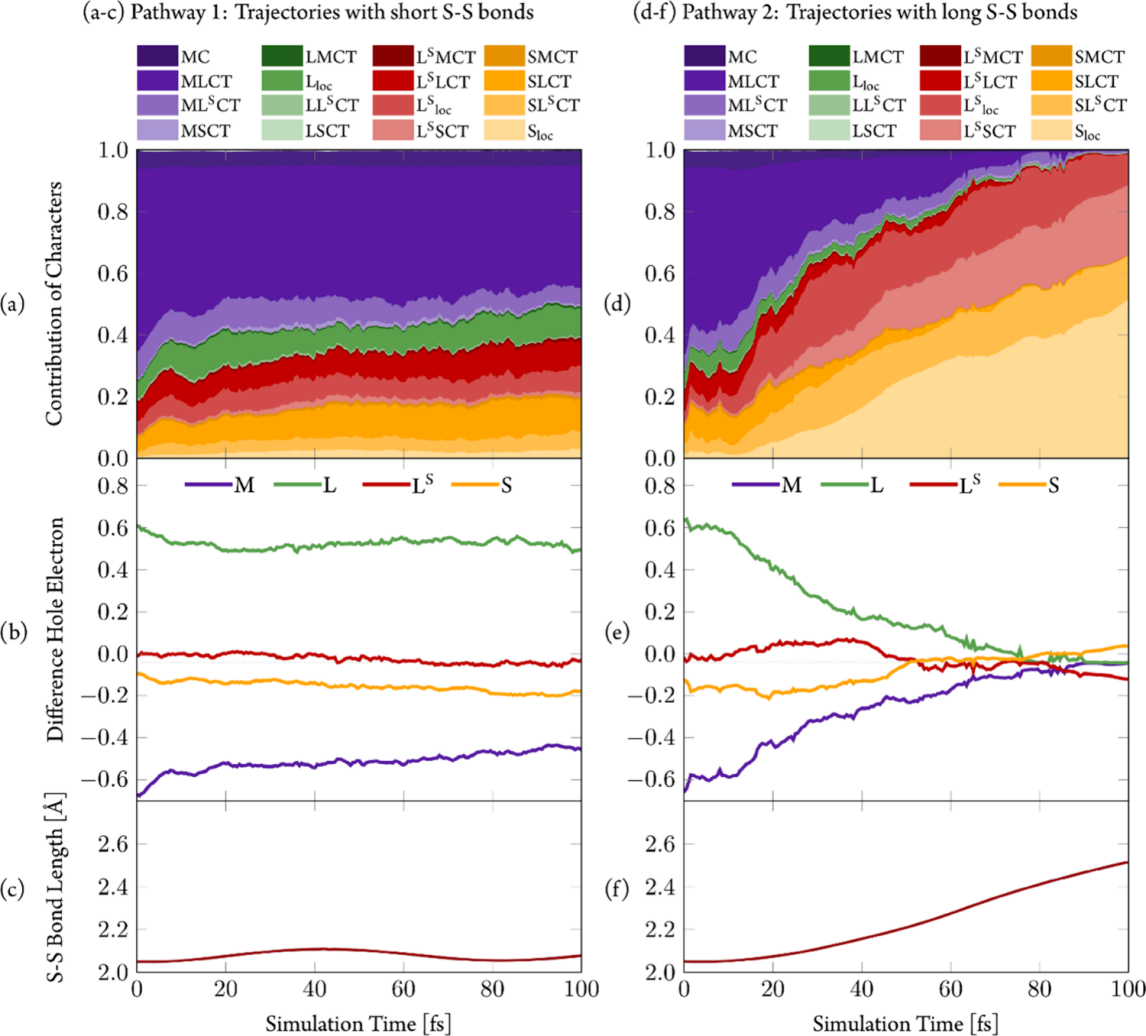
Transition density matrix analysis of the trajectories in pathway 1 (a–c, short S–S bonds) and pathway 2 (d–f, long S–S bonds). (a/d) Character of trajectories. (b/e) Electron–hole difference population. (c/f) S–S bond length. All quantities averaged over all trajectories in the respective pathway.

Upon comparing the electronic characters of the trajectories (Figure 10), significant differences emerge. Pathway 1 shows steady behavior (Figure 10a–c), which can be characterized as MLCT and therefore does not show significant changes in S–S bond length. Pathway 2, on the other hand, undergoes significant changes (Figure 10d,e). The trajectories start initially also in states with predominant MLCT character, but during the simulation time, the S–S bond increases (Figure 10f). This leads to stabilization of the disulfide-centered state compared to the MLCT, and the latter is replaced by local excitations at the disulfide unit (S_loc_) and—to a smaller extent—local excitations at the ^S–S^bpy^4,4^ core (L^S^_loc_). Charge transfer excitations from the disulfide unit to the ^S–S^bpy^4,4^ core (SL^S^CT) and back (L^S^SCT) also contribute significantly to the electronic wave functions of these trajectories. However, the similar extent of these CT excitations quenches any actual charge flow. Their presence in the electronic character of the excitation (Figure 10d), nevertheless, is a manifestation of the multiconfigurational character of the excited states.

This behavior shows a simple mechanism for the pathway 2 trajectories (Section S6.1). Initially staying in MLCT states around the Franck–Condon geometry, these trajectories at some point switch to states with dominant S_loc_+L^S^_loc_ character. From them, the S–S bond starts to increase, leading to the T_1_^SSlong^ (^3^LC) geometry. For comparison, trajectories following pathway 1 (Section S6.2) remain mostly in MLCT states, and even if they temporarily switch to electronic states of different character, e.g., involving ligand-to-ligand charge transfer (L^S^LCT), they always return to MLCT electronic states.

### Long-Time Linear Vibronic Coupling Based Nonadiabatic Dynamics Simulations

The large computational cost of *on-the-fly* surface-hopping simulations can be alleviated by parametrizing the TDDFT potential energy surfaces with model potentials according to the linear vibronic coupling (LVC) approach (Section S7.1).^77^ LVC potentials are fitted with a Taylor expansion around a reference geometry and expressed on the basis of the (harmonic) normal modes of this geometry. The description of the LVC potentials is thus only accurate close to this reference geometry. The use of LVC potentials should be restricted to rather rigid molecules, as is often the case for transition-metal complexes. The suitability of this “rigidity approximation” for **1**^+^ is discussed in Section S7.2. In combination with LVC potentials, trajectory surface hopping allows efficient dynamics simulations of systems including dozens of electronic states and hundreds of nuclear degrees of freedom during several ps.^78^

**Figure 11:**
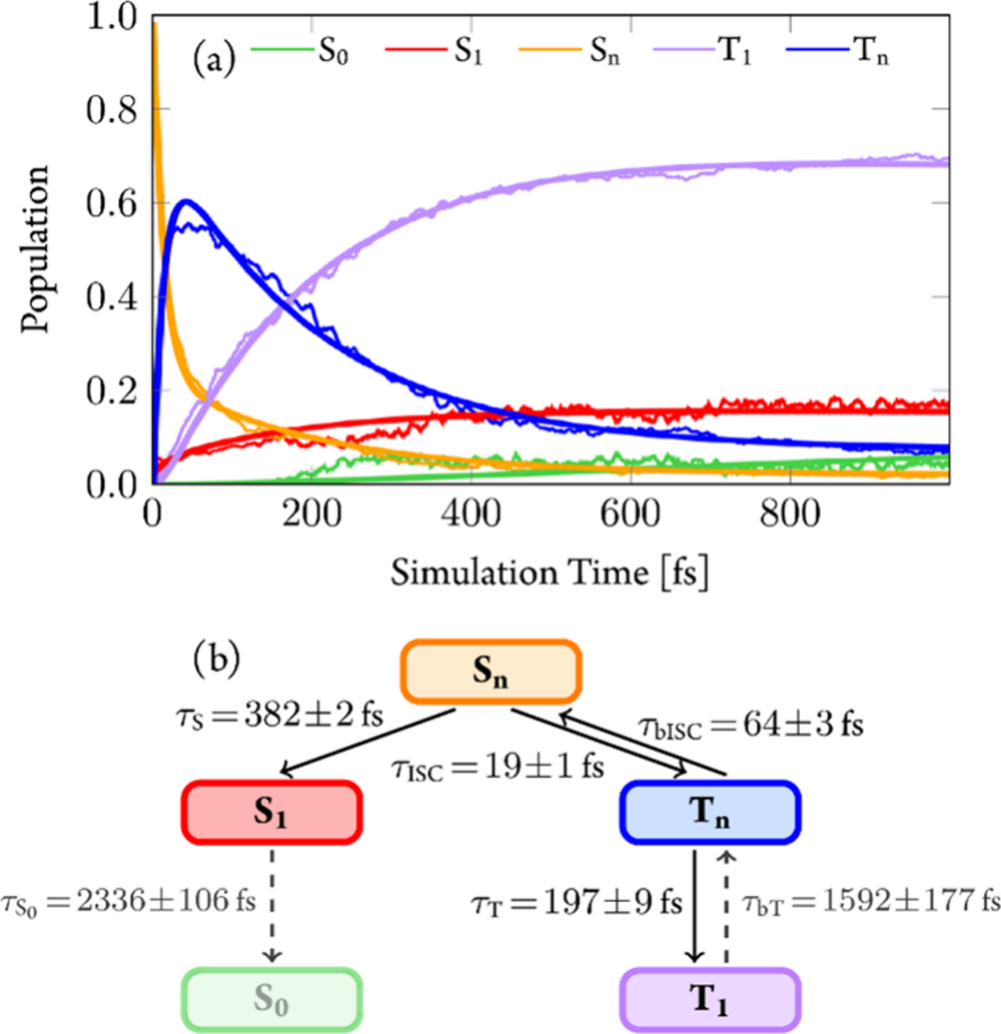
(a) Time evolution of the adiabatic electronic-state populations (thin lines) during the LVC/SH simulations and fits (thick lines) corresponding to the mechanism shown in (b).

Figure 11a shows the time evolution of the adiabatic electronic-state populations of the LVC/SH simulations carried out during 1 ps. The first 100 fs resembles the behavior of the TDDFT/SH dynamics (recall Figure 9a and see a direct comparison in Section S7.3), giving us confidence in the LVC model, at least for short times. The resulting kinetic model (Figure 11b) returns the fits shown in Figure 11a by thick lines.

Similar to the TDDFT/SH dynamics, most of the electronic population starting from higher-lying singlet states S_*n*_ undergoes ISC to high-lying triplet states T_*n*_ before relaxing to the T_1_. A small fraction of population relaxes from the singlet manifold S_*n*_ to the S_1_. After 150 fs, population also starts to deactivate from the S_1_ into the S_0_ ground state. It is questionable whether such a process can be described correctly (or at all) within the present LVC/SH approach, due to the difficulties of (TD)DFT to describe S_1_/S_0_ conical intersections.^79^ Fortunately, S_1_ → S_0_ internal conversion is only a minor reaction channel here. Furthermore, this channel does not influence the dynamics en route to the T_1_, our state of interest. We note that no transfer from the S_1_ to the S_0_ was observed in the TDDFT/SH dynamics, nor in selected trajectories propagated up to 150 and 200 fs. A comment on the character of the S_1_ state is given at the end of this section.

**Figure 12:**
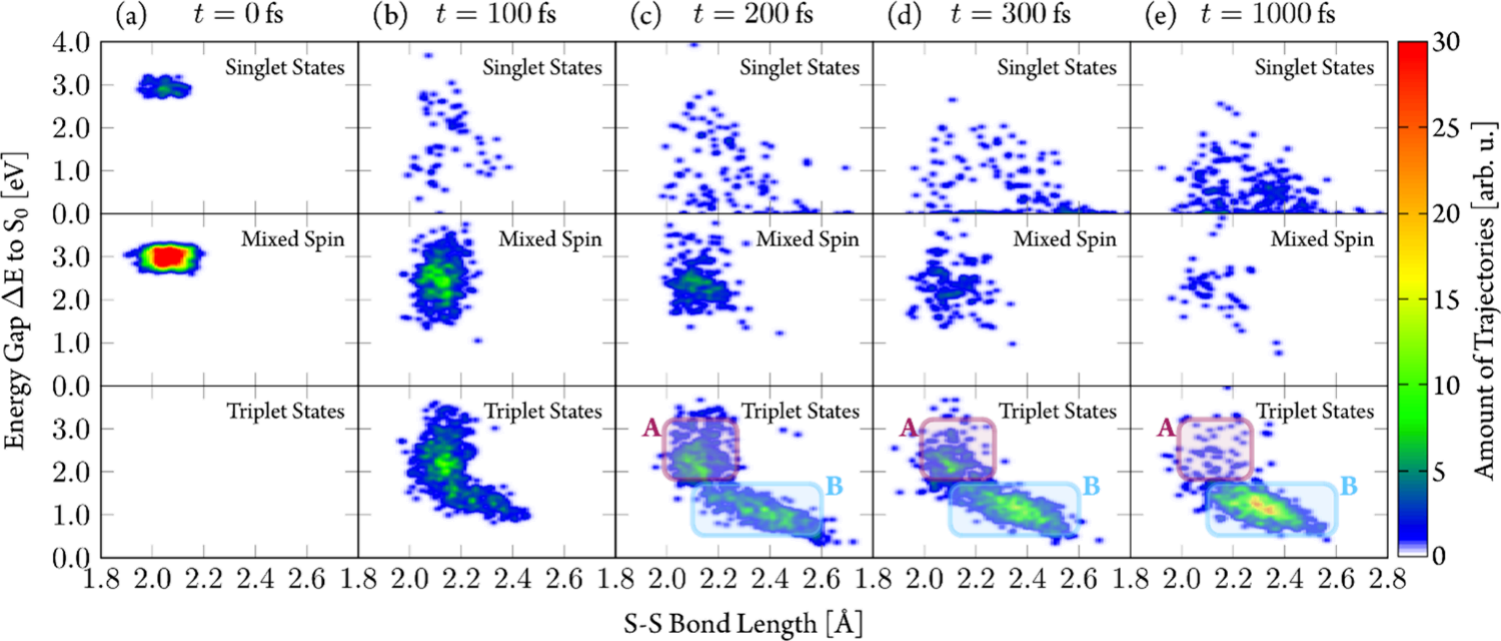
Energy gap Δ*E* to the ground state S_0_ as a function of the S–S bond length of LVC/SH trajectories at different simulation times. Trajectories distinguished by spin expectation value as singlet states (⟨*S*^2^⟩ < 0.2), mixed spin states (0.2 < ⟨*S*^2^⟩ < 1.8), and triplet states (⟨*S*^2^⟩ > 1.8). Regions A and B identify clusters of trajectories characterized by energy gaps (Δ*E*) of 2.0 eV and S–S bond lengths of 2.1 Å (purple rectangle) and energy gaps (Δ*E*) around 1.0 eV and S–S bond lengths of 2.3–2.5 Å (blue rectangle), respectively.

We recall the two relaxation pathways to the triplet states observed in the TDDFT/SH dynamics: pathway 1, where molecules keep a geometry close to the initial equilibrium one, and pathway 2, where the geometries display elongated S–S bonds. We investigated both pathways in the LVC/SH dynamics by plotting (Figure 12) the energy gap distribution between the active state and the S_0_, Δ*E*, of each trajectory as a function of the S–S bond length at different times. Thereby, we divide trajectories according to their spin expectation values: singlet states (⟨*S*^2^⟩ < 0.2), triplet states (⟨*S*^2^⟩ < 1.8), and mixed-spin states (0.2 < ⟨*S*^2^⟩ < 1.8). The trajectories start at excitation energies around 3.0 eV with S–S bond lengths around 2.1 Å and mixed-spin states (Figure 12a). After 100 fs, a large fraction of trajectories is still in spin-mixed states (b), which are now found at lower energy gaps Δ*E*. As the time progresses, the number of mixed-spin trajectories decreases, ending in either “pure” triplet or singlet states. For singlet trajectories, a rapid decrease in energy gap Δ*E* is seen already after 100 fs, accompanied by a wide spread over S–S bond lengths. Interestingly, we found Δ*E* close to zero along a large range of S–S bond lengths (2.0–2.7 Å), indicating that the S_1_ → S_0_ IC is not localized in a narrow area.

After 100 fs, the majority of trajectories have entered the triplet manifold and show smaller energy gaps. The distribution of S–S bond lengths has still the largest contributions in the initial region around 2.1 Å, indicating that ISC takes place. However, a fraction of trajectories moves toward longer S–S bond lengths up to 2.4 Å. This process is continued at later times with more triplet trajectories moving toward smaller energy gaps Δ*E* and longer S–S bond lengths.

**Figure 13:**
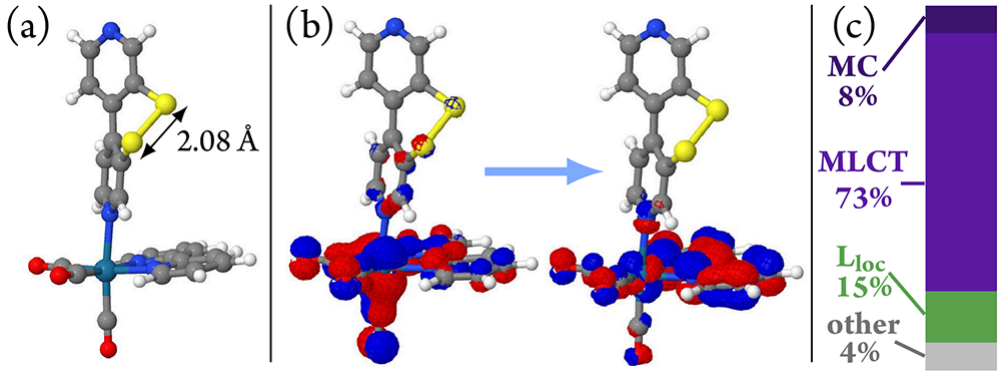
(a) Optimized geometry of the lowest lying triplet state starting from the snapshots of region A of the LVC/SH dynamics. (b) Natural transition orbitals characterizing the T_1_ state at this geometry, labeled T_1_^SSshort^ state. (c) Wave function analysis of the T_1_^SSshort^ state showing its predominant ^3^MLCT character, so that this state will be also referred to as the ^3^MLCT state.

After 200 fs simulation (c), the triplet trajectories appear to cluster around two regions: one characterized by energy gaps Δ*E* of 2.0 eV and S–S bond lengths of 2.1 Å (region A, purple rectangle in Figure 12) and another with energy gaps Δ*E* around 1.0 eV and S–S bond lengths of 2.3–2.5 Å (region B, light blue rectangle). Over time, trajectories move toward region B, where almost all of the population ends up after 1 ps. The S–S bond elongation in region B is reminiscent of the pathway 2 trajectories from the TDDFT/SH dynamics where trajectories reached much larger S–S bond lengths of 2.6–3.0 Å. The different extent of bond elongation, however, is likely due to the way in which the LVC potentials are constructed, in particular because the basis of harmonic normal modes prevents too large displacements away from the reference geometry (Section S7.2). Yet, it seems likely that region B trajectories from the LVC/SH dynamics correspond to the pathway 2 trajectories from the TDDFT/SH dynamics (Figure 9c). This raises the question whether the trajectories of region A from the LVC/SH dynamics also correspond to pathway 1 from the TDDFT/SH dynamics and whether both region A and region B trajectories can give clues about the fate of pathway 1 and 2 trajectories. To answer this question, we have taken snapshots from trajectories in region A and optimized their structures, first on the LVC potential energy surface and subsequently using TDDFT (Sections S7.4 and S7.7). Remarkably, many optimizations starting from region A resulted in a new T_1_ minimum characterized by a short S–S bond length of 2.08 Å and thus referred to as T_1_^SSshort^. Figure 13 shows the optimized triplet geometry, its associated natural-transition orbitals, and transition-density matrix analysis. This T_1_^SSshort^ is mostly characterized by an excitation from the Re d orbitals—mixed with CO contributions—to the equatorial bpy ligand (L), so it will be also referred to as the ^3^MLCT state.

The wave function analysis predicts 73% of MLCT character, 8% of local excitations at the Re(CO)_3_ fragment (MC), and 15% of local excitations at the bpy ligands (L_loc_). This is different from the character of the initially excited states in the dynamics, which contained contributions involving the ^S–S^bpy^4,4^ ligand (Figure 10a/d at *t* = 0 fs).

Interestingly, the energy gap to S_0_ for the T_1_^SSshort^ (2.08 eV) is close to the experimentally observed phosphorescence maximum (2.16 eV), suggesting that this is the triplet state responsible for phosphorescence. Support of this assignment comes from the character of the T_1_^SSshort^ (^3^MLCT) state, which is typical for long-lived emissive states in other Re^I^(CO)_3_(diimine) complexes.^45,61^ We will confirm this preliminary assignment when explaining the time-resolved experiments in the next section. Before doing so, we still need to address the fate of the pathway 2/region B trajectories and discuss the possible interconversion between the two triplet minima.

In the LVC/SH dynamics, the majority of the trajectories end up in region B. This might be an artifact of the approximate nature of the LVC potentials (Section S6.3), which due to their harmonic shape do not allow for S–S bonds longer than the ones accessible in the reference TDDFT potentials. Importantly, the TDDFT potential energy surface exhibits a T_1_/S_0_ crossing point close to the T_1_^SSlong^ (^3^LC) minimum. The energy barrier separating the T_1_^SSlong^ minimum and this T_1_/S_0_ minimum energy crossing point is only 0.14 eV, making it accessible to the TDDFT/SH trajectories of pathway 2. In contrast, the corresponding T_1_/S_0_ crossing point in the LVC potential energy surface lies ca. 3 eV above the T_1_ minimum in region B due to the harmonic shape (Section S7.2). Thus, the LVC/SH trajectories are trapped in region B, unable to relax to the S_0_. This is in contrast to the on-the-fly TDDFT/SH trajectories that can be expected to undergo ISC back to the ground state S_0_ at later simulation times due to the presence of the low-lying S_0_/T_1_ minimum-energy crossing point.

As both the TDDFT/SH and LVC/SH dynamics predict rapid relaxation to the T_1_ potential energy surface on a 200–250 fs time scale, the long-time excited-state dynamics are majorly governed by its shape. We believe that the T_1_^SSshort^ (^3^MLCT) minimum is responsible for the experimentally observed phosphorescence, while the region around the T_1_^SSlong^ (^3^LC) minimum gives access to an additional nonradiative deactivation channel back to the S_0_ ground state. Thus, it is interesting to discuss how both regions are connected on the T_1_ surface. The T_1_^SSshort^ (^3^MLCT) minimum is thereby found close to the Franck–Condon region at an adiabatic excitation energy of 2.25 eV (relative to the global S_0_ minimum), stabilized compared to the vertical excitation energy of the T_1_ state at the global S_0_ minimum of 2.42 eV. The T_1_^SSlong^ minimum is farther away from the Franck–Condon region and, thus, geometrically more distinct with an adiabatic excitation energy of 1.56 eV (Section S7.5). Attempts to find a transition state on the T_1_ potential energy surface connecting both minima using the nudged-elastic band method were unsuccessful; a linear interpolation between both minima showed a small barrier of ca. 0.1 eV in the triplet surface (Section S7.5). Potential energy scans along the S–S bond length upon relaxing both the S_0_ and T_1_ states showed no barrier in the T_1_ state upon elongation of the S–S bond length, while the T_1_ state adopts predominantly ^3^LC character at all relaxed geometries. This suggests that once a triplet state assumes ^3^LC character, its fate is bound to relax toward the T_1_^SSlong^ (^3^LC) minimum.

In both TDDFT/SH and LVC/SH simulations, we observed a small part of the electronic population in the S_1_ state. While we do not think that this part plays an important role in the relaxation mechanism, we performed additional geometry optimizations in the lowest singlet excited state S_1_ to characterize its potential energy landscape, as described in detail in Section S8 in the SI. Interestingly, we find two distinct minima, S_1_^SSlong^ and S_1_^SSshort^ with long (2.57 Å) and short (2.08 Å) S–S bond lengths, with ^S–S^bpy^4,4^ local-excitation character and MLCT excitation characters, respectively, thus mirroring the feature of the lowest triplet potential energy surface.

## Discussion

### Interpretation of Experimental Data

The transient IR spectra of Figure 4b show two excited-state components, which are formed on a sub-picosecond time scale and feature lifetimes of 20 ± 3 ps and <1 ns. The decay of the 20 ps component accounts for 90% of the ground-state recovery, whereas 10% of the excited-state population is long-lived beyond the experimental time scale of 1 ns. The experimentally determined photoluminescence quantum yield for [**1**]PF_6_ of ϕ_total_ = 0.3% is about 1 order of magnitude smaller than similar Re carbonyl diimine systems (cf. 3% for [Re(CO)_3_(bpy)(py)]PF_6_ or 9% for [Re(CO)_3_(bpy)(4,4′-bpy)]PF_6_,^64,80^ in complete agreement with the fast ^3^LC/S_0_ relaxation channel swiftly removing 90% of the excited-state population and leaving η = 10% in the ^3^MLCT state, which is responsible for the observed photoluminescence spectrum. Parallel fast population of two excited states, where one is the long-lived ^3^MLCT, is consistent with the UV–vis transients of Figure S8 showing formation of the bpy^•–^ radical anion at 375 nm on a sub-picosecond time scale. The emission maximum of the ^3^MLCT at 2.16 eV agrees nicely with the calculated S_0_/T_1_^SSshort^ energy gap of 2.08 eV. The lifetime of the long-lived component corresponds to the measured luminescence decay time of 270 ns, implying radiative and nonradiative rate constants for ^3^MLCT of *k*_r_ = 1.1 × 10^5^ s^–1^ and *k*_nr_ = 3.6 × 10^6^ s^–1^, respectively. The radiative rate constant of 10^5^ s^–1^ is commonly found in Re diimine complexes emitting purely from a ^3^MLCT state.^62^

These observations strikingly agree with our nonadiabatic simulations exhibiting two triplet populations emerging within 200–250 fs. Part of the trajectories end up in the so-called T_1_^SSlong^ (^3^LC) state (pathway 2, corresponding to the ^3^LC state), which is located close to a T_1_/S_0_ crossing point, enabling fast ground-state recovery by ISC. The other trajectories follow pathway 1 toward the T_1_^SSshort^ (^3^MLCT) configuration (corresponding to the ^3^MLCT state) in the vicinity of the ground-state equilibrium geometry. Accordingly, we attribute the experimentally observed 20 ps component to the decay of the ^3^LC state and the long-lived photoproduct to the ^3^MLCT state. As the elongation of the S–S bond requires major structural changes, we attribute the 1 ps component visible in the IR and UV–vis transients to geometrical relaxation of the axial ^S–S^bpy^4,4^ ligand while approaching the T_1_^SSlong^ (^3^LC) minimum.

This assignment is supported by the computed CO vibrational stretching frequencies of the S_0_ (Figure 4c, green sticks) and the two triplet minima T_1_^SSlong^ (blue sticks) and T_1_^SSshort^ (purple sticks). The calculations predict two split CO frequencies at 1966 and 1979 cm^–1^ and one at 2049 cm^–1^. Compared to the experimentally observed bands (Figure 4a), these frequencies are slightly blue-shifted by 15–50 cm^–1^, yet the splitting of the two low-frequency modes is nicely reproduced (see analysis of the FTIR spectrum in Figure S6). The two sets of calculated T_1_ stretching frequencies are blue-shifted with respect to the S_0_ state, indicating a decrease of electron density at the metal center leading to less back-bonding into π* orbitals of the CO ligands. The MLCT state directly involves metal oxidation, explaining the origin of the blueshift. For T_1_^SSlong^ (^3^LC), the blueshift is less pronounced and can be due to the 22% L^S^SCT portion of this state, which is characterized by a πσ* transition, removing electron density from the π-system of the ^S–S^bpy^4,4^ ligand (Figure 8) and thereby increasing its π-acceptor abilities. This leaves less electron density to be donated to the CO ligands. Because the charge flows toward the disulfide which is largely decoupled from the remaining framework, this can be considered a “real” charge transfer removing electron density from the aromatic system that electronically communicates with the rhenium metal center. This leads to an overall smaller blueshift of the CO bands and agrees with the time evolution of the transient absorption spectra (Figure 4b) showing directly after excitation positive features at 1960, 2000, and 2050 cm^–1^, which decay (τ = 20 ps) and uncover long-lived and more blue-shifted bands at 1970, 2030, and 2065 cm^–1^. During the initial phase, the spectra of the two states are superimposed, with ^3^LC being responsible for the majority of the intensity since it is populated with 90%. After this short-lived state has decayed back to the ground state, the spectral features of the long-lived ^3^MLCT are uncovered, accounting for only 10% of the total population. During the decay of the ^3^LC state, the high-energy A′(1) band undergoes a dynamic blueshift. A similar effect was also observed for related Re diimine complexes—yet without simultaneous decay of the A′(1) band—and attributed to vibrational relaxation and/or solvent-assisted electronic relaxation of the two triplet states.^41,45^ In our case, we attribute the blueshift to vibrational relaxation of the ^3^LC. An alternative interpretation based on reaction of a vibrationally hot ^3^MLCT toward the ^3^LC state can be excluded (Section S12).

### Excited-State Dynamics Mechanism

**Figure 14:**
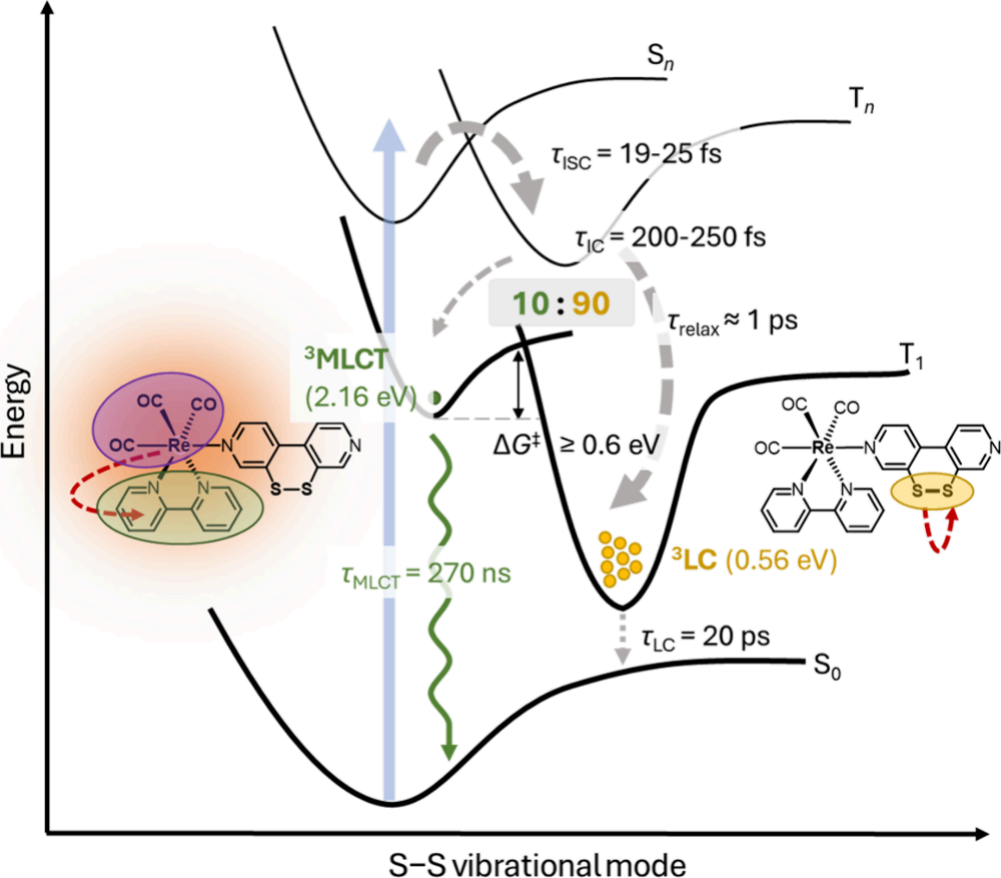
Energy diagram of the excited-state dynamics mechanism of [**1**]^+^.

The interpretation of the TRIR experiments together with our dynamics simulations allows us to formulate a comprehensive excited-state relaxation mechanism of **1**^**+**^ after irradiation, summarized in Figure 14. After excitation with UV light, the major relaxation pathway is the population of the triplet states. In both TDDFT/SH and LVC/SH dynamics, we obtain a time constant of τ_ISC_ ≈ 20 fs for the ISC from the (initially excited) higher-lying singlet states S_*n*_ to the higher-lying triplet states T_*n*_. The character of the initial excitation is ^1^MLCT, which is carried over to the higher-lying triplets. In the triplet manifold, relaxation down to the T_1_ takes place along two pathways: In pathway 1, the system keeps close to the Franck–Condon geometry, and so it ends up in the T_1_^SSshort^ minimum, a ^3^MLCT state. In pathway 2, the system enters a pseudo-dissociative state described by a local excitation in the sulfurated ligand, populating the σ*(S–S) orbital, a ^3^LC state. The latter pathway drives the system toward longer S–S bonds, which requires substantial geometrical restructuring of the ^S–S^bpy^4,4^ ligand; however, the disulfide does not dissociate since the system ends up in the T_1_^SSlong^ (^3^LC) minimum.

Both pathways occur simultaneously on a time scale of τ_T_ ≈ 200 fs with a 10:90 ratio of pathway 2 being favored over pathway 1. Close to the ^3^LC minimum there is a T_1_/S_0_ crossing that allows the system to relax nonradiatively to the ground state with a time constant of τ_LC_ = 20 ps. The ^3^MLCT species is long-lived with a lifetime of τ_MLCT_ = 270 ns at 298 K and an efficiency for radiative decay from ^3^MLCT of about 3% (i.e., 10 times ϕ_total_, accounting for η = 10% probability of populating the ^3^MLCT state), indicating that nonradiative decay is still the dominant relaxation channel. This raises the question why part of the population is trapped in the higher-lying ^3^MLCT state, instead of funneling into the lower ^3^LC state. In order to prevent IC, communication between both states is hindered, either due to a reasonable barrier between the ^3^MLCT and ^3^LC states or because electronic coupling between the two states is negligible; that is, they do not interact. Attempts to optimize a transition state failed. We also examined the temperature dependence of the ^3^MLCT lifetime (Section S1.3). In the temperature range from 5 to 50 °C, the lifetime decreases only slightly, which is compatible with a barrierless ISC back to the ground state, as observed for rhenium diimine complexes before.^81^

This left us to conclude that if there is a barrier between ^3^MLCT and ^3^IC states, this is so high that in the considered temperature range (5–50 °C), the ^3^MLCT → ^3^LC reaction is negligible. Based on transition state theory and assuming a pre-exponential factor of 10^12^ s^–1^, we calculated a lower limit for the free energy of activation Δ*G*^⧧^ of 0.6 eV to observe no temperature dependence of the photoluminescence lifetime in the range 5 to 50 °C (Section S1.3), resulting from this IC process. This energy is similar to the error of our TDDFT protocol, explaining why localization of the barrier is difficult.

**Scheme 2:**
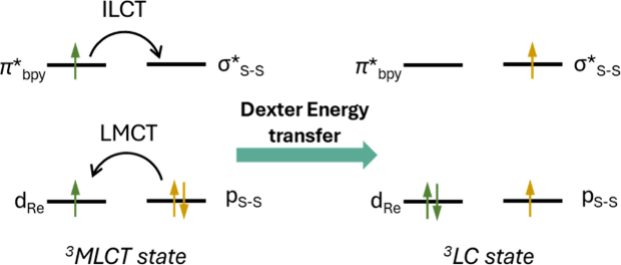
Orbital View of the Dexter Energy Transfer Process between the MLCT and LC States via Two-Electron Exchange Only the orbitals with the highest charge transfer character are shown.

This barrier can be interpreted chemically from a Marcus-theory point of view: the reorganization energy due to rearrangement of bonds during the transition, especially the disulfide– and metal–ligand bonds, has to be quite substantial, therefore slowing down the formal electron transfer, which is the IC process.^82^ The IC between the two orthogonal ^3^MLCT and ^3^LC states formally involves the transfer of two electrons within four different orbitals. In a qualitative way, this process can be depicted (Scheme 2) using intramolecular energy transfer from the excited chromophore to the disulfide via a Dexter mechanism.^43^ One electron is transferred in an ILCT from the π* of the bpy ligand to the σ* of the disulfide bond, and the other one via an LMCT from the disulfide to the rhenium metal center.

The rate of IC is slow if either the electronic wave functions are poorly overlapping or there is a large energy gap between the two states.^8,11,25,36^ Both conditions are reasonably fulfilled in our case, since the disulfide moiety is largely decoupled from the aromatic system,^16^ and the two states have a calculated energy gap of 1.6 eV; therefore, IC is not occurring and the bifurcation happens already at earliest timescales. The interconversion of two excited states via IC has been reported before by Vlček and co-workers for an axially styrylpyridine-substituted rhenium complex. From their TA, they observe complete IC to the lowest-energy ^3^IL state within 3.5 ps, preventing the formation of any long-lived emissive state (for their *trans*-isomer).^43^ However, in our case, the ^S–S^bpy^4,4^ ligand is distorted, which greatly decreases the rate of electron exchange, as shown for a dirhenium (3,3′-dimethyl-4,4′-bpy)-bridged complex.^39^ Thus, Figure 14 represents the most plausible mechanism for the bimodal excited-state decay of **1**^+^. We disregarded a mechanism based on the reaction of a vibrationally hot ^3^MLCT toward the ^3^LC state, as it would require that the reaction rate constant *k*_r_ of the vibrationally hot ^3^MLCT toward ^3^LC can compete against vibrational relaxation; we show this is not the case in Section S12. Furthermore, the relative yield of short-lived (90%) and long-lived (10%) population would depend on excess vibrational energy dumped by the pump photon into the ^3^MLCT. However, for the two pump wavelengths 266 and 400 nm, the same population ratio is observed (Figures 4 and S7). Additionally, our data suggest that both ^3^MLCT and ^3^LC are populated ultrafast, directly after the pump pulse (see also Figures S26 and S35).

Finally, we discuss the small, but non-negligible, portion of trajectories that initially deactivate to the singlet manifold. Since spin–orbit coupling in 5d metals is large, fluorescence represents only a negligible relaxation process on ultrafast (<100 fs) time scales.^54^ Due to the limitations of the simulations, the fate of these trajectories could not be followed up. Also, we did not observe any sign of further relaxation to the ground state in the experiments. In the TRIR spectra, we do not observe the typical broad, red-shifted bands caused by repopulation of the S_0_ with excess vibrational energy. However, it is unclear whether the absence of any experimental sign is due to the low population of the singlet manifold or an overestimation of the simulations, or whether the singlet population can indeed return and join either of the two observed pathways 1 and 2.

Our proposed mechanism with emission from a higher-lying state due to slow IC to the low-energy state represents an exception to Kasha’s rule.^8,11^ There have been examples of similar energy transfer schemes in rhenium complexes involving the (CO)_3_Re(bpy) chromophore and varying axial ligands (notably with a styrylpyridine in axial position),^43^ yet these systems exhibit no emission from the higher-lying state due to fast and therefore nearly complete IC to the lowest energy state. The unique properties of **1**^**+**^ also open new avenues for our series of disulfide-decorated complexes (Figure 1, **A**, **B**). While previously, charge transfer and emission were observed from disulfide-centered states at lower energies compared to the parent complexes lacking the disulfide group, now we are able to trap a certain part of the excited-state population in a higher-lying, emissive state, which can be used for excited-state bimolecular reactivity, as opposed to the lower-energy LC-centered state. We therefore gain enough driving force and lifetime for **1**^**+**^ to take part in light-induced bimolecular reactivity. This leaves the question of what are the design principles guiding this exceptional behavior. Chemically speaking, the reason is the orthogonality of the (CO)_3_Re(bpy) chromophore unit and the electron-accepting (and therefore lower-energy) disulfide moiety, leading to weak electronic and vibrational coupling, decelerating electron transfer. In all other systems, the charge-accepting unit is coupled to some kind of aromatic π-system with the ligands (e.g., alkenes, extended aromatic rings, azo-groups),^37,39,48,83−86^ which is not the case in our disulfide functionality. One of the key factors appears to be the structural responsiveness of the disulfide unit, which leads to large structural changes due to severe elongation of the S–S bond in the ^3^LC state, viz., to a large reorganization barrier upon internal conversion. The disulfide is still an electron acceptor, since 90% of population flows toward it, yet a noticeable portion of the population is trapped in the higher-lying triplet state. The excitation energy therefore is used more efficiently through emission via this anti-Kasha state.

## Conclusion

The photosensitizer [Re^I^(CO)_3_(bpy)(^S–S^bpy^4,4^)]PF_6_ ([**1**]PF_6_) has been developed, based on the popular rhenium-tricarbonyl-diimine scaffold but featuring an axially coordinated ^S–S^bpy^4,4^ ligand—a 4,4′-bpy ligand equipped with a disulfide bridge in the 3,3′-positions—for which a convenient two-step synthesis is reported. In contrast to complexes with the related chelating ligand ^S–S^bpy^2,2^ (cf. **A**, **B**, Figure 1), the peripheral disulfide unit in **1**^**+**^ can readily respond to changes in the S–S bond length by adjusting the torsion of the two pyridine subunits around the central C–C bond of ^S–S^bpy^4,4^, while not being strongly connected electronically to the aromatic framework. The excited-state dynamics of **1**^**+**^ have been studied by steady-state and time-resolved spectroscopic methods and elucidated mechanistically by full-dimensional nonadiabatic dynamics simulations. The latter has demonstrated its predicting power, beyond empirical interpretations of experimental data or stationary calculations of vibrational frequencies. The structurally responsive S–S bridge opens an unprecedented relaxation channel in the excited-state dynamics, different from nonfunctionalized rhenium-carbonyl-diimine complexes.

After excitation, **1**^**+**^ populates ^1^MLCT states involving the equatorial bpy ligand, from which ultrafast ISC to the triplet manifold takes place. Further, **1**^**+**^ follows two distinct pathways populated on a 200 fs time scale in a 10:90 ratio, leading to two triplet states of very different nature: The lesser populated pathway 1 leads to a higher-energy ^3^MLCT state involving the equatorial bpy ligand, similar in character to the initially excited singlet states. This triplet state is long-lived (270 ns) and emits orange light (at 570 nm) with a photoluminescence quantum yield of around 3%, leading to the observed overall photoluminescence quantum yield of **1**^**+**^ of 0.3%. The dominant pathway 2 involves a locally excited state (^3^LC) at the ^S–S^bpy^4,4^ ligand with an electron transferred into the antibonding σ* orbital of the S–S unit. This lower-energy state relaxes through elongation of the S–S bond but before disulfide dissociating reaches a triplet minimum, denoted T_1_^SSlong^ (^3^LC), that is, close to a T_1_/S_0_ crossing point. Through this channel, **1**^**+**^ can relax back to the ground state in a nonradiative manner on a 20 ps time scale. The interconversion between the two states via intramolecular energy transfer is prevented sufficiently due to weak electronic and vibrational coupling, giving rise to an effective barrier of >0.6 eV. This makes phosphorescence from the higher-lying ^3^MLCT state competitive. To the best of our knowledge, **1**^**+**^ is the first rhenium diimine complex displaying anti-Kasha emission behavior reported to date. This anti-Kasha state helps to minimize losses in excitation energy. We anticipate that the decoupling of the excited state located in the (CO)_3_Re(bpy) chromophore and lowest-energy state located in the disulfide unit has significant implications for bimolecular quenching experiments, which are currently being studied in our laboratories.

For applications in molecular wiring or optical sensors, knowledge of the influence of substitution on the photophysical properties is crucial. We intend to exploit the design principle developed in this work, in order to further increase the portion of the population in the emissive state, yet still having the disulfide functional group, which can undergo chemical transformations. The availability of the ^3^LC relaxation channel in this and related complexes could possibly also be manipulated via interaction of the disulfide unit with chemical agents such as exogenous metal ions or protons (pH control) or hydrogen bond donors including biomolecules such as nucleotides. In the case of ^S–S^bpy^4,4^, also the peripheral N atom of the ligand is available for protonation, hydrogen bonding, or additional metal ion coordination, which provides a means for further tuning of the excited-state dynamics. Future (time-resolved) resonance Raman measurements could be used to directly monitor the evolution and structural responsiveness of the disulfide moiety upon excitation, serving as a complementary method to the transient IR spectroscopy.

Understanding the relaxation mechanism was made possible by integrating theoretical calculations with experimental data. Further nonadiabatic simulations including explicit solvation within a hybrid quantum mechanical/molecular mechanics model might unveil specific solute–solvent interactions that could compete with the inherent nuclear relaxation dynamics.

## Experimental Section/Methods

Details about the manipulations, starting materials, and instruments used for compound characterization are provided in the Supporting Information (Section S1.1).

### Synthesis of the Ligand ^S–S^bpy^4,4^

3,3′-Dibromo-4,4′-bipyridine (1.0 g, 3.2 mmol, 1.0 equiv) was suspended in dry Et_2_O (100 mL) and cooled to −94 °C. ^*n*^BuLi (2.5 M in hexane, 2.8 mL, 7.0 mmol, 2.2 equiv) was added dropwise. The reaction mixture turned orange and was stirred for 45 min at that temperature. Then, sulfur (1.6 g, 6.4 mmol, 2.0 equiv) was added in small portions. The flask was removed from the cold bath after 10 min and stirred overnight at r.t. The reaction mixture was quenched with water (100 mL), and the organic layer was separated. The aqueous phase was extracted with CH_2_Cl_2_ (3 × 100 mL), dried over Na_2_SO_4_, and concentrated. Column chromatography on silica (4:1 CH_2_Cl_2_/acetone) gives the title compound as a bright yellow solid (yield: 18%). Slow evaporation of CH_2_Cl_2_ gave crystals suitable for X-ray diffraction. The analytical data are in good agreement with the ones already published.^57^

### Synthesis of [Re(CO)_3_(bpy)(^S–S^bpy^4,4^)]PF_6_ ([**1**]PF_6_)

Re(CO)_3_(bpy)(OTf) (150 mg, 0.26 mmol, 1.0 equiv) was dissolved in CH_2_Cl_2_ (25 mL), Na[BAr^F^_4_] (231 mg, 0.26 mmol, 1.0 equiv) was added, and the mixture stirred for 5 min. This solution was then added dropwise to a solution of ^S–S^bpy^4,4^ (285 mg, 1.30 mmol, 5.0 equiv) in CH_2_Cl_2_ (15 mL), and the resulting reaction mixture was stirred for 1 h in the dark. The mixture was filtered and concentrated to a volume of around 3 mL under reduced pressure, and the product was purified by column chromatography on silica (gradient of 100% acetone to 100:20 acetone/sat. aq. KNO_3_). A sat. aq. KPF_6_ solution (3 mL) was added, and the organic solvent removed under reduced pressure. The precipitating solid was extracted with CH_2_Cl_2_ (3 × 15 mL), the CH_2_Cl_2_ phase dried over Na_2_SO_4_ and filtered, and the solvent evaporated. Crystallization by layering of a CH_2_Cl_2_ solution with hexane afforded [**1**]PF_6_ as yellow crystalline solid (yield: 78%). The excess ligand is recovered after column chromatography by evaporation of the solvent under reduced pressure, redissolving in CH_2_Cl_2_, filtration through a glass fiber filter, and evaporation of the solvent. ^1^H NMR (600 MHz, thf-*d*_8_): δ (ppm) = 9.28 (ddd, *J* = 5.5, 1.5, 0.7 Hz, 2H), 8.77 (s, 1H), 8.69 (s, 1H), 8.67 (d, *J* = 8.2 Hz, 2H), 8.60 (d, *J* = 5.2 Hz, 1H), 8.37 (td, *J* = 8.0, 1.6 Hz, 2H), 8.14 (dd, *J* = 6.0, 0.6 Hz, 1H), 7.88 (d, *J* = 5.9 Hz, 1H), 7.86 (ddd, *J* = 7.7, 5.5, 1.2 Hz, 2H), 7.71 (dd, *J* = 5.2, 0.5 Hz, 1H), 5.51 (s, 2H, CH_2_Cl_2_ contained in the crystal). ^13^C{^1^H} NMR (126 MHz, thf-*d*_8_): δ (ppm) = 196.4, 157.0, 154.6, 152.4, 152.2, 151.8, 150.0, 146.1, 142.5, 141.3, 137.1, 132.8, 129.9, 126.4, 125.9, 54.9. ^15^N NMR (51 MHz, thf-*d*_8_): 240.8 (bpy), 237.7 (^S–S^bpy^4,4^). ESI(+)-MS (CHCl_3_): *m*/*z* (rel. int.) = 645 [M]^+^ (100), 427 [M – (^S–S^bpy^4,4^)]^+^ (10), 455 [Re(bpy)(CO)_3_N_2_]^+^ (1). ATR-IR: ν (cm^–1^) = 2030 (s, ν_CO_), 1931 (s, ν_CO_), 1908 (s, ν_CO_), 1602 (m), 1470 (m), 1441 (m), 1399 (m), 828 (s, ν_PF_), 765, 727. IR (KBr): ν (cm^–1^) = 2033 (s, ν_CO_), 1934 (s, ν_CO_), 1912 (s, ν_CO_), 842 (s, ν_PF_). UV–vis (THF, *c*_opt_ = 2.5 × 10^–5^ M): λ_max_ (nm)/ε (10^3^ cm^–1^ M^–1^) = 272 (sh, 19), 312 (17), 322 (19), 336–420 (sh, 11–0). Elem. Anal. Calc. for C_22_H_14_N_4_O_3_ReS_2_PF_6_·CH_2_Cl_2_ (there is one molecule of CH_2_Cl_2_ present in the unit cell, which is not removed under reduced pressure): C 32.96, H 1.84, N 6.41, S 7.33. Found: C 33.15, H 2.05, N 6.35, S 7.40.

### X-ray Crystallography

X-ray data for [**1**]PF_6_ were collected on a STOE IPDS II diffractometer (monochromated Mo Kα radiation, λ = 0.71073 Å) by use of ω or ω and φ scans at low temperature. The structures were solved with SHELXT and refined on *F*^2^ using all reflections with SHELXL.^87,88^ Non-hydrogen atoms were refined anisotropically. Hydrogen atoms were placed in calculated positions and assigned to an isotropic displacement parameter of 1.2*U*_eq_(C). Face-indexed absorption corrections were performed numerically with the program X-RED.^89^ CCDC 2237141 contains the supplementary crystallographic data for this paper. These data can be obtained free of charge from The Cambridge Crystallographic Data Centre via www.ccdc.cam.ac.uk/structures.

### Transient UV-Pump–Vis-Probe Setup

UV/vis pump–probe experiments were performed with a 1 kHz Ti:sapphire oscillator/regenerative amplifier system (Solstice Ace, Spectra Physics) producing 35 fs laser pulses at 800 nm. Pump pulses at 400 and 266 nm were generated by second- and third-harmonic generation, respectively, attenuated to pulse energies of below 1 μJ, and focused to a diameter of about 200 μm at the sample. A white-light continuum generated by focusing a small portion of the 800 nm light in a CaF_2_ crystal of 4 mm thickness was used for probing. About 50% of the white light was used to record a reference spectrum. The other half was for probing pump pulse induced changes in the spectrum by superimposing both beams at the center of the sample cell. A synchronized chopper blocked every second pump pulse to determine difference spectra with and without the pump pulse. The relative plane of polarization of pump and probe light was adjusted to 54.7°. The spectra of reference and probe continua were each recorded at wavelengths of 350–730 nm with a 256-element linear diode array attached to a spectrograph. A translation stage (M-415.DG, Physik Instrumente) was used to adjust the time delay between pump and probe pulses. The measured time-dependent transient spectra were corrected for a wavelength-dependent temporal shift introduced by group delay dispersion within the white-light-probe continuum. Experiments were performed with a hermetically sealed quartz glass cell of 2 mm optical path length filled under an argon atmosphere. A magnetic stirrer was included to avoid accumulation of photoproducts in the laser focus.

### Transient UV-Pump–IR-Probe Setup

This experiment is based on a 1 kHz Ti:sapphire oscillator/regenerative amplifier system (Coherent, Libra) producing 100 fs pulses at 800 nm. As in the previous setup pump pulses were generated by second- (400 nm) and third-harmonic (266 nm) generation. Tunable mid-IR probe pulses with a bandwidth of about 200 cm^–1^ were generated by difference frequency mixing of idler and signal pulses from a home-built two-stage optical parametric amplifier^90^ pumped by 0.5 mJ of the regenerative amplifier output. The mid-IR beam was split into a reference and a probe beam. The probe pulse passed a translation stage and was superimposed with the pump pulse in the sample cell. Probe and reference pulses were directed to a polychromator and separately detected by a liquid-nitrogen-cooled HgCdTe-detector (Infrared Associates Inc.) with two linear arrays of 32 pixels each. The spectral resolution was 4.5 cm^–1^/pixel; that is, a single measurement covered a spectral range of about 130 cm^–1^. The transients of Figure 4b were generated from 5 sets of measurements with overlapping spectral ranges each shifted by 40 cm^–1^. The mid-IR beam path was purged with dry nitrogen to minimize pulse distortions by CO_2_ and water absorptions. The hermetically sealed stainless-steel sample cell equipped with two CaF_2_ windows of 1 mm thickness and a magnetic stirrer had an optical path length of 0.6 mm and comprised a total volume of 3 mL (Section S1.2 for the schematics). The sample was excited with pulse energies of 2 μJ focused to a diameter of 200 μm. No significant reduction of signal intensity or accumulation of photoproducts was observed during data acquisition.

### Computational Details

The excited-state dynamics of **1**^**+**^ were simulated using trajectory surface hopping.^91,92^ Two sets of simulations were performed, one where the electronic-state potentials were calculated on-the-fly using TDDFT with the PBE0 functional^93^ and double-ζ quality basis sets,^94,95^ and another using an LVC model parametrized at the same TDDFT level of theory.^77,96^ The behavior of the TDDFT potentials along S–S bond elongation was benchmarked against multireference calculations.^97^ Excited-state minima in the lowest-energy triplet state T_1_ and minimum-energy crossing points between the S_0_ and T_1_ were optimized with TDDFT. Electronic states were characterized using a transition-density matrix analysis.^70^ Full computational details are reported in Sections S2–S7.

### Photoluminescence Quantum Yield Determination

The photoluminescence quantum yield ϕ in THF was determined relative to [Re(CO)_3_(bpy)(py)]PF_6_ (py = pyridine)^98^ in DCE according to eq 2 from ref (99), with the known quantum yield ϕ of the reference standard [Re(CO)_3_(bpy)(Etpy)]PF_6_ (Etpy = 4-ethylpyridine) in DCE of 13.5%,^39^ the integrated emission intensity *I*, the absorbance at excitation wavelength *A*, and the refractive indices for THF (*n* = 1.405) and DCE (*n* = 1.4422).^100^
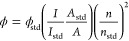
2

## Supporting Information Available

Computational details, additional analysis of the excited-state dynamics simulations, as well as experimental methods (PDF)
LVC template (ZIP)
