# Supplementary material for: Bifurcation of Excited-State Population Leads to Anti-Kasha Luminescence in a Disulfide-Decorated Organometallic Rhenium Photosensitizer
Source: J Am Chem Soc. 2024 Apr 10;146(16):11272–88. doi: 10.1021/jacs.4c00548 (PMC11046484; doi:10.1021/jacs.4c00548)
Supplement: Supplementary file 1 — ja4c00548_si_001.pdf [file ja4c00548_si_001.pdf]

SUPPORTING INFORMATION

for

Bifurcation of Excited-state Population leads to anti-Kasha Luminescence in a Disulfide-decorated Organometallic Rhenium Photosensitizer

Julia Franz,[a,#] Manuel Oelschlegel,[b,#] J. Patrick Zobel,[a,#] Shao-An Hua,[b] Jan-Hendrik Borter,[c] Lucius Schmid,[e] Giacomo Morselli,[e] Oliver S. Wenger,[e] Dirk Schwarzer,[c,]* Franc Meyer,[b,d,]* and Leticia González[a,f,]*

[a] Institute of Theoretical Chemistry, University of Vienna, Währinger Straße 17, A-1090 Vienna, Austria

[b] Institute of Inorganic Chemistry, Georg-August Universität Göttingen, Tammannstr. 4, D-37077 Göttingen, Germany

[c] Department of Dynamics at Surfaces, Max-Planck-Institute for Multidisciplinary Sciences, Am Faßberg 11, D-37077 Göttingen, Germany

[d] International Center for Advanced Studies of Energy Conversion (ICASEC), D-37077 Göttingen, Germany

[e] University of Basel, Department of Chemistry, St.-Johanns-Ring 19, CH-4056 Basel, Switzerland

[f] Vienna Research Platform for Accelerating Photoreaction Discovery, University of Vienna, Währinger Straße 17, A-1090 Vienna, Austria

[#]These authors contributed equally

Corresponding Authors

*E-Mail: leticia.gonzalez@univie.ac.at
*E-mail: franc.meyer@chemie.uni-goettingen.de
*E-mail: dschwar@mpinat.mpg.de

Table of Contents

S1 Experimental Section 4

S1.1 Spectroscopic Characterization 4

S1.1.1 Materials and Methods 4

S1.1.2 NMR Spectrum of the Ligand $^{S\text{-}S}bpy^{4,4}$ 4

S1.1.3 Characterization of $[Re(CO)_3(bpy)(^{S\text{-}S}bpy^{4,4})]PF_6$ ($[1]PF_6$) 5

S1.2 Transient Absorption Spectroscopy 7

S1.3 Temperature-dependent measurement of the phosphorescence lifetime 9

S1.4 Comment on Photostability of 1^+ 10

S2 Calculation of the Absorption Spectrum 10

S2.1 Computational Details 10

S2.2 Comparison of Functionals 11

S2.3 Influence of Solvent and of Spin-Orbit Coupling on the Absorption Spectrum 15

S2.4 Transition-Density Matrix Analysis 16

S3 Triplet Optimization 18

S3.1 Computational Details 18

S3.2 Comparison of Different Triplet Energies 18

S4 TDDFT Surface Hopping Dynamics 19

S4.1 Computational details 19

S4.2 Hops in the Short-Time TDDFT Nonadiabatic Dynamics 20

S4.3 Note on the Rate of Intersystem Crossing in the Simulation 21

S4.4 Evolution of S-S Bond Length of Trajectories Propagated for 200 fs 22

S5 Potential Energy Scan Along the S-S Bond of the Ligand $^{S\text{-}S}bpy^{4,4}$ 22

S5.1 Discussion 22

S5.2 Computational Details 26

S5.3 Influence of the IPEA Shift and State Mixing in CASSCF/CASPT2 Calculations 28

S6 Transition-Density-Matrix Analysis of Trajectories 30

S6.1 Pathway 2 Trajectories 30

S6.2 Pathway 1 Trajectories 30

S6.3 Electronic State Populations 31

S7 Long Time LVC Surface Hopping Dynamics 32

S7.1 Computational Details 32

S7.1.1 Parametrization of the LVC Model Hamiltonian 32

S7.1.2 LVC Absorption Spectrum 32

S7.1.3 Surface Hopping Dynamics 33

S7.2 Comparison of LVC Potentials and TDDFT Potentials 33

S7.3 Comparison of LVC/SH and TDDFT/SH Dynamics 34
S7.4 Triplet Optimization from Snapshots of the LVC/SH Dynamics 35
S7.5 Connection Between the Two Triplet Minima 36
S7.6 Electronic state populations 38
S8 Excited-State Singlet Minima 39
S9 Charge Redistribution in the Triplet Minima 40
S10 Parameters 42
S11 Geometries of Optimized Structures 42
S12 Other Possible Disregarded Mechanisms 48
S13 Crystallographic Details 50
S14 References 51

S1 Experimental Section

S1.1 Spectroscopic Characterization

S1.1.1 Materials and Methods

All manipulations were performed under an atmosphere of dry argon, unless otherwise stated. THF was purchased in HPLC quality (Sigma Aldrich), dried over NaK alloy, distilled, degassed and stored over 3Å molecular sieves. CH_2Cl_2 was degassed and stored over molecular sieves. Silica gel 60 (Sigma Aldrich) was used for column chromatography. 3,3'-dibromo-4,4'-bipyridine,[1] and $Re(bpy)(CO)_3OTf$ [2] were synthesized following modified literature procedures. All other chemicals were obtained commercially and used as received: $Re(CO)_5Cl$ (Sigma Aldrich), AgOTf (Sigma Aldrich), 2,2'-bipyridine (TCI), $Na[BAr^F{}_4]$ (BLD Pharmatech, $[BAr^F{}_4]^-$ = tetrakis(3,5-bis(trifluoromethyl)phenyl)borate).

NMR spectra were recorded on Bruker Avance III 300, Avance III HD 400 or Avance Neo 600 spectrometers. Chemical shifts (δ) are reported in ppm relative to residual solvent signals (thf-d_8: 3.58, 1.72 ppm, $CDCl_3$: 7.26 ppm). The following abbreviations were used for signal multiplicities: s (singlet), d (doublet), t (triplet), m (multiplet), br (broad). If not stated otherwise, all spectra were recorded at room temperature (r.t.).

IR spectra were measured with an Agilent Technologies Cary 630 FTIR spectrometer with Dial Path Technology and analyzed with FTIR MicroLab software.

ESI mass spectra were measured on a Bruker maXis ESI-QTOF machine.

Elemental analyses were obtained from the Analytical Laboratory of the Institute of Inorganic Chemistry at Georg-August-University Göttingen, using an Elementar Vario EL III analyzer.

UV-vis absorption spectra were recorded on a Cary 5000 instrument from Varian. A Fluorolog 3-22 instrument from Horiba Jobin-Yvon was employed for steady-state luminescence spectroscopy. The luminescence decay was measured on a LP920-KS apparatus from Edinburgh Instruments, equipped with a frequency-tripled pulsed Nd:YAG laser (Quantel Q-smart 450, ca. 10 ns pulse width).

S1.1.2 NMR Spectrum of the Ligand $^{S\text{-}S}bpy^{4,4}$

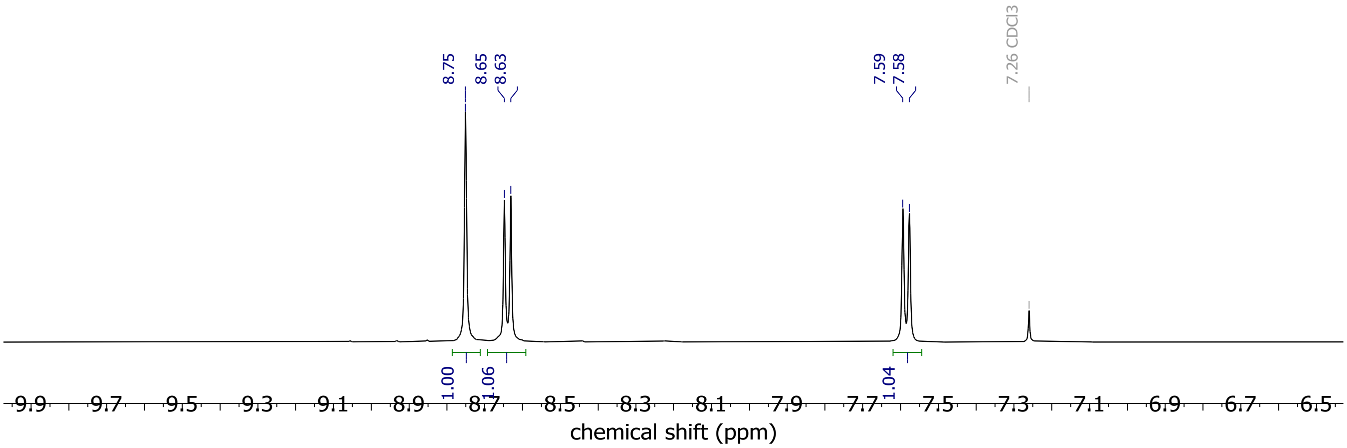

Figure S1. ^{1}H NMR spectrum of $^{S\text{-}S}bpy^{4,4}$ in $CDCl_3$.

S1.1.3 Characterization of $[Re(CO)_3(bpy)(^{S\text{-}S}bpy^{4,4})]PF_6$ ($[\mathbf{1}]PF_6$)

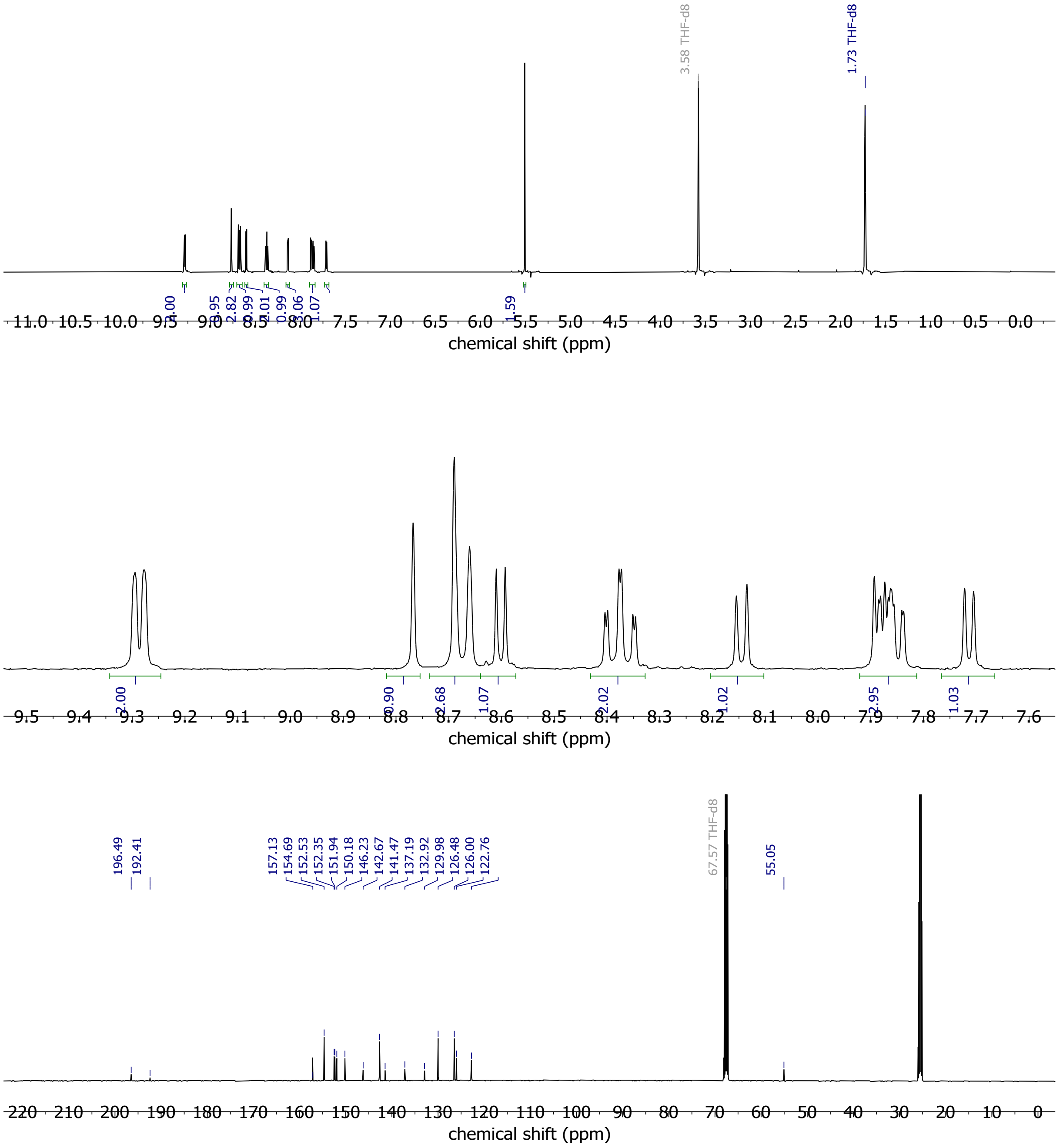

Figure S2. ^{1}H (top, middle) and $^{13}C\{^{1}H\}$ (bottom) NMR spectra of $[\mathbf{1}]PF_6$ in thf-d_8.

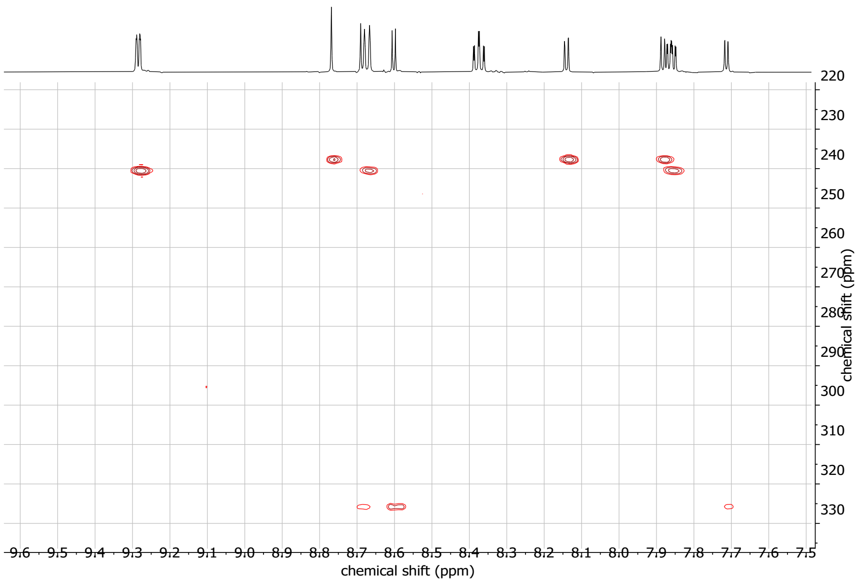

Figure S3. $^{1}H/^{15}N$ HMBC NMR spectrum of [**1**]PF_6 in thf-d_8.

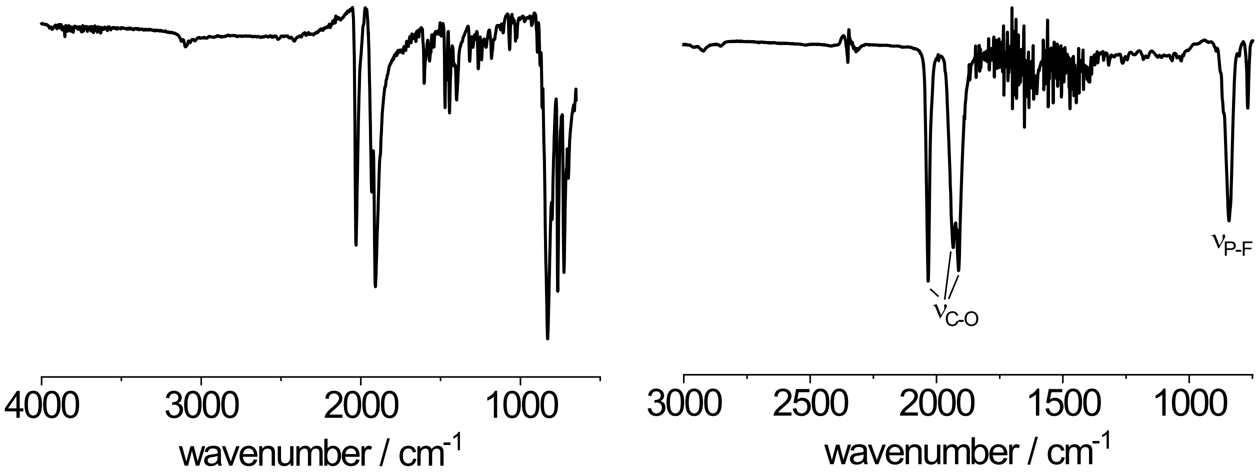

Figure S4. IR spectra of [**1**]PF_6 (ATR: left; KBr pellet: right).

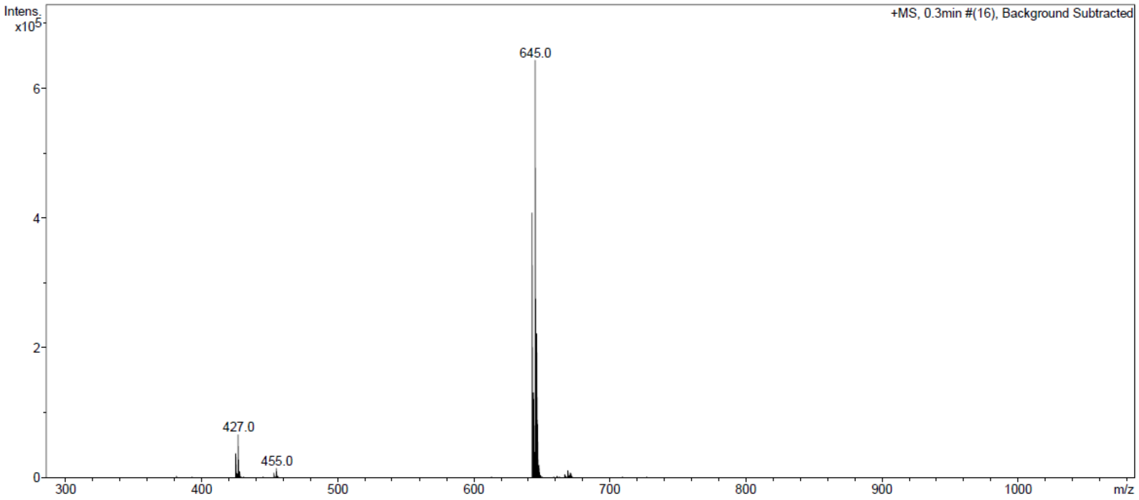

Figure S5. ESI(+) mass spectrum of [**1**]PF_6 in $CHCl_3$.

S1.2 Transient Absorption Spectroscopy

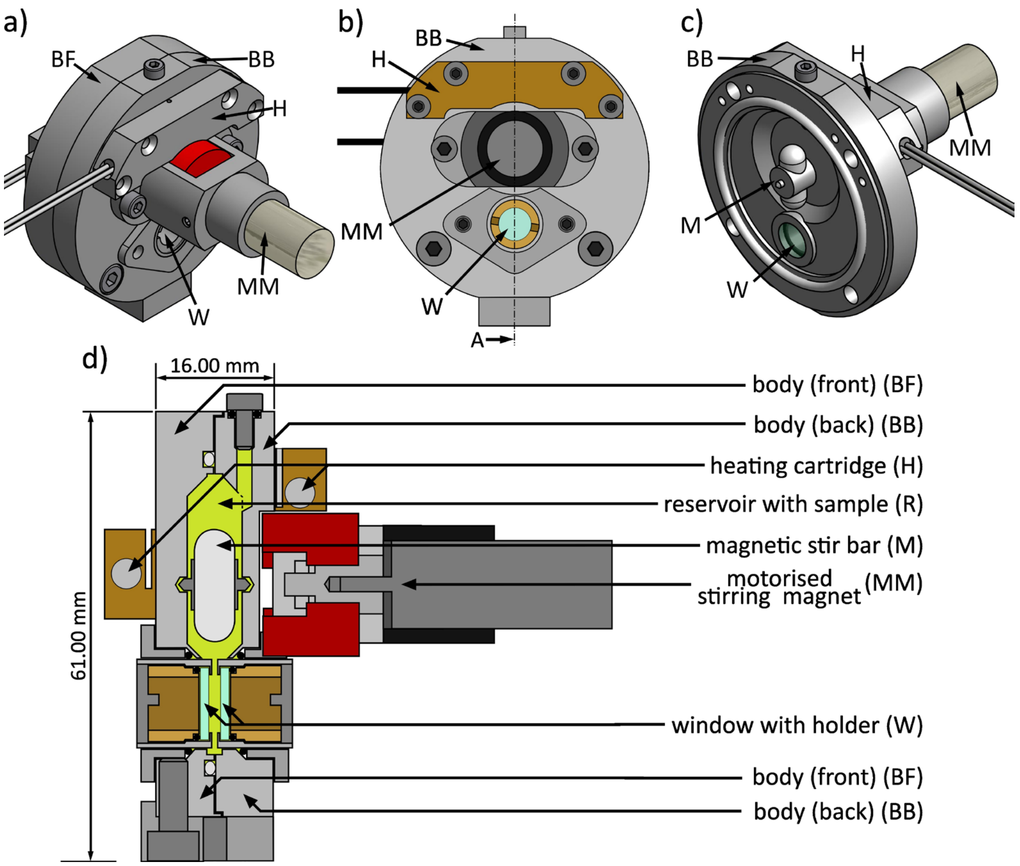

Schematic S1. Schematics of the hermetically sealed stainless-steel cell. a) angled view of the back side, b) rear view, c) angled view of the inner side of the back half, d) cross section perpendicular to b) at point A.

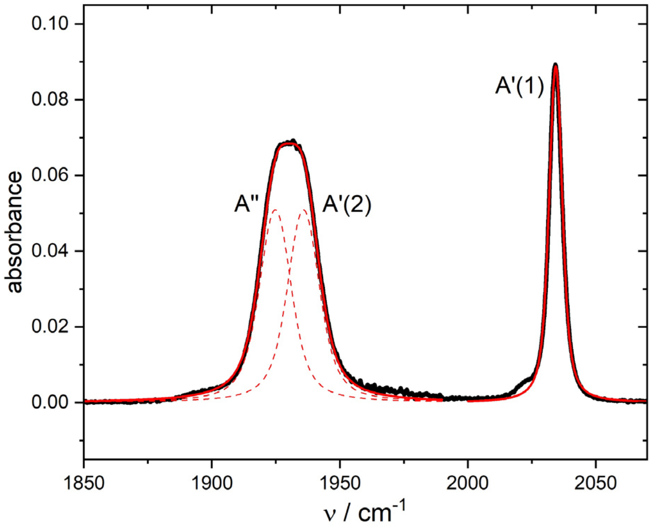

Figure S6. The FTIR spectrum of [1]PF$_6$ in THF (black) and fitted Voigt profiles (red); the contrib tions of the overlapping A'' and A'(2) bands are plotted by dashed red lines. The derived cent quencies are A'': 1924.8 cm^{-1}, A'(2): 1935.8 cm^{-1}, A'(1): 2034.3 cm^{-1}.

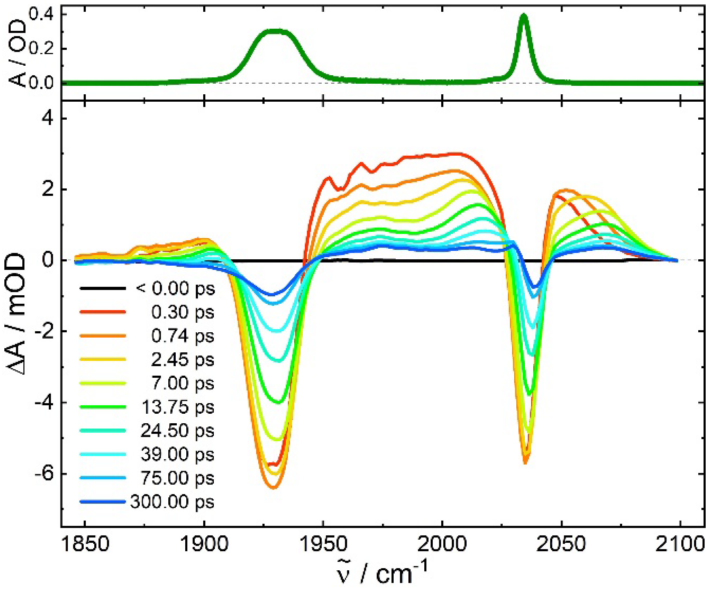

Figure S7. Transient IR difference spectra of [**1**]PF$_6$ in THF recorded after 266 nm excitation at indicated time delays.

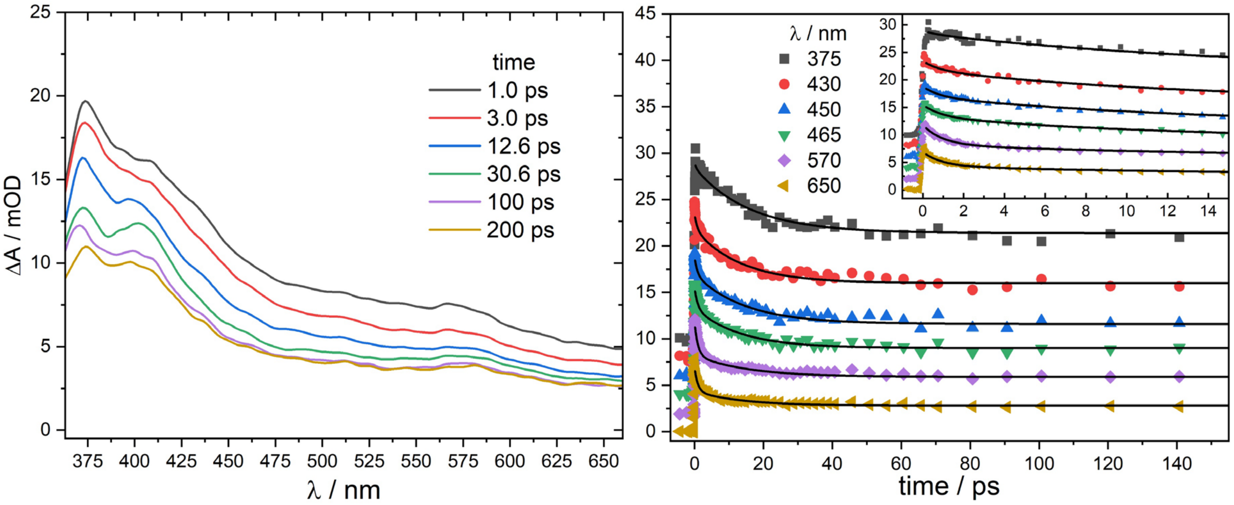

Figure S8. Left: Transient UV-vis difference spectra of [**1**]PF$_6$ in THF recorded after 340 nm ex citation at indicated time delays. Right: Time traces at indicated probe wavelengths (for cla trace is offset by 2 mOD); black lines are double exponential fits with global time constants τ_1 = 15±3 ps and τ_2 = 0.8±0.2 ps, and varying offset and amplitude ratios. Note, that these UV-vis transients are not superimposed by spontaneous emission. This explains the difference to the 20 ns spectrum of Figure 3 of the main paper, which due to a different experimental setup collects luminescence light causing negative absorption with a peak at 573 nm.

S1.3 Temperature-dependent measurement of the phosphorescence lifetime

A solution of **1** (0.1mM) in deaerated THF was analyzed by the TCSPC technique in the temperature range of 5 to 50°C (Figure S9a). Excitation occurred with a pulsed laser at 375 nm, detection occurred at 570 nm. The ratio of stop/start counts remained at <2%. The sample cuvette was degassed with argon after each measurement. Photoluminescence lifetime studies were performed on an FLS1000 spectrometer (time-correlated single photon counting technique) from Edinburgh Instruments using a pulsed LED (EPL-375 from Edinburgh Instruments, pulse width: 61.7 ps, linewidth for excitation at 375.2 nm), where the cuvette holder was equipped with a temperature controller (TC 1, Quantum Northwest).

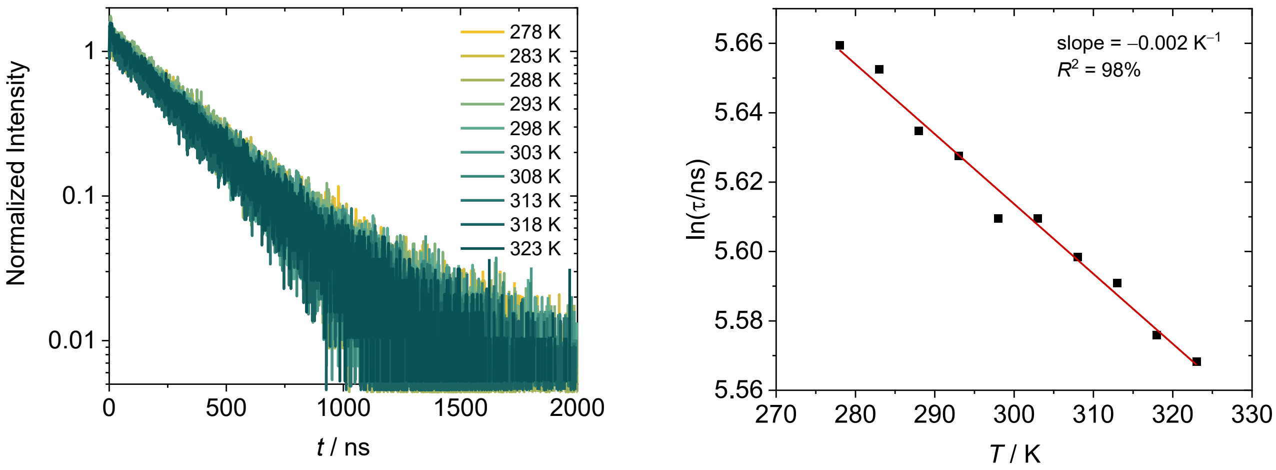

Figure S9a. Left: Time-resolved emission decay at 570 nm at different temperatures. Right: Plot of ln(τ) vs. *T*, with linear fit.

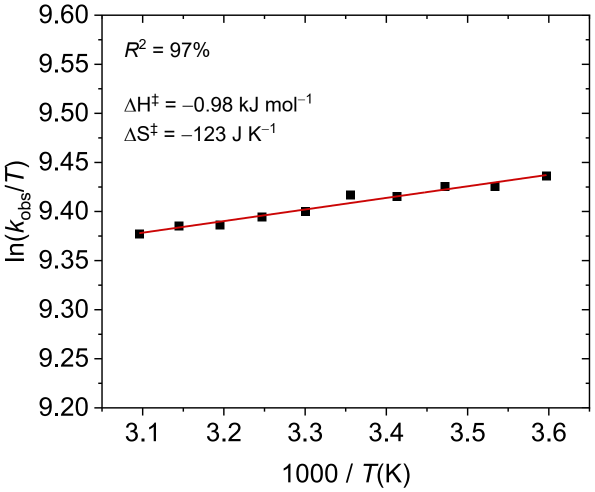

Figure S9b. Eyring Plot of ln(k_{obs}/T) vs. 1000/T(K) with linear fit and activation parameters.

Eyring analysis (Figure S9b) returns typical non-radiative decay via a barrierless transition back to the ground-state, of which the very low value for $\Delta H^{\ddagger}$ is indicative.
In order for the two states to be populated, internal conversion must be slow. Based on the radiative lifetime of the ^{3}MLCT state, and the measured temperature range from 278 to 323 K, we can estimate a lower limit of the activation energy for the IC process.

$$k_{\mathrm{obs}} = A \exp\left(-\frac{E_{\mathrm{a}}}{k_{\mathrm{B}}T}\right) \quad \text{(S1)}$$

$$\text{with: } k_{\mathrm{r}} + k_{\mathrm{nr}} = (270\ \mathrm{ns})^{-1};\ A = 10^{12}\ \mathrm{s}^{-1};$$

$$k_{\mathrm{obs}}^{-1} = (k_{\mathrm{r}} + k_{\mathrm{nr}} + k_{\mathrm{IC}})^{-1} \approx 270\ \mathrm{ns};\ k_{\mathrm{IC}} = (50\mu\mathrm{s})^{-1}$$

$$\boldsymbol{E_{\mathrm{a}} \geq 0.55\ \mathrm{eV}}$$

S1.4 Comment on Photostability of $\mathbf{1}^+$

$\mathbf{1}^+$ was irradiated, with strong stirring, in a 10 mm fluorescence cuvette in deaerated THF with Kessil® PR160L LEDs at 390(±20) and 427(±25)nm positioned ca. 2 cm away from the sample cuvette. Upon irradiation for ca. 1.1 photons/molecule (calculated using the absorbed light at the respective wavelength, and the duration of the irradiation), no changes in absorbances were detected (Figure S10). The incoming light was detected using a Thorlabs S120VC (200-1100 nm, 50 mW) photodiode detector.

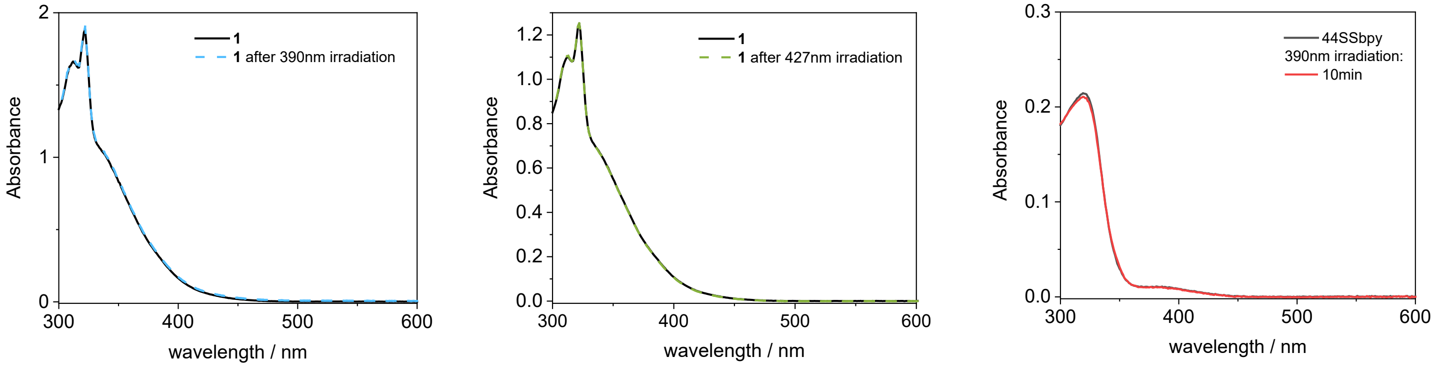

Figure S10. Photoirradiation of $\mathbf{1}^+$ at 390 (left) and 427nm (center) and free $^{\mathrm{S\text{-}S}}$bpy^{4,4} at 390nm (right). No change in absorbance was detected, indicating photostability.

Likewise, we performed these photon counting measurements for our transient absorption measurements, where we did not observe accumulation of photoproduct. From these experiments, we conclude that $\mathbf{1}^+$ is photostable under our experimental conditions.

S2 Calculation of the Absorption Spectrum

S2.1 Computational Details

Calculations in this section were performed using the ORCA4.2.1 program package.[3]

To find the ground state of $\mathbf{1}^+$, a scan of geometry optimizations along the Re-N bond length was performed (see Figure 6 in the main paper). Close to the minima in the potential, unconstrained geometry optimizations were performed. For these optimizations, the singlet ground-state structure of $\mathbf{1}^+$ was calculated with density functional theory (DFT) using the PBE0 functional,[4] the SARC-ZORA-TZVP[5] basis set for Re and the ZORA-def2-TZVP[6] basis set for the other elements (C, O, N, S, H) and the D4 dispersion correction.[7] For the self-consistent field calculations, the resolution-of-identity approximation (RIJCOSX),[8] the Grid4 integration grid and tight convergence criteria (TightSCF) were used. Scalar relativistic effects were incorporated with the zeroth-order regular approximation (ZORA).[9] The four resulting structures showed no modes with imaginary frequencies, confirming the identity of the minima.

To determine a suitable method to describe the excited states of **1**$^+$, time-dependent DFT (TDDFT) was used with the functionals PBE0, B3LYP,[10] CAM-B3LYP,[11] LC-BLYP,[12] and M06-2X.[13] Additionally, the long-range correction parameters of LC-BLYP were reoptimized specifically for **1**$^+$, to obtain the fitted functional LC-BLYP*.[14] The parameters employed are α=0.19, a variable exchange of 85%, a fixed HF exchange of 15%, and a fixed DFT exchange of 0%. For the TDDFT calculations, the Tamm-Dancoff approximation,[15] the D4 dispersion correction, ZORA, and the basis sets at the triple-zeta level from the ground-state optimizations were employed.

For the lowest-energy minimum **IV** (see Figure 6 in the main paper), an ensemble of 50 geometries were generated from a Wigner distribution at T=300 K.[16,17] At each geometry 30 singlet and 30 triplet excited states were calculated. The resulting stick spectra for each functional were convoluted using Lorentzian functions and a full-width at half-maximum of 0.25 eV. For the PBE0 functional, a larger ensemble of 550 geometries from Wigner distributions including all four minima according to their Boltzmann populations at T=300 K was generated, and the spectrum was recalculated.

The influence of solvent effects on the spectrum was investigated for tetrahydrofuran (THF) as solvent. For this, the geometry optimization and excited-state calculations were repeated using SMD solvent model.[18]

S2.2 Comparison of Functionals

The absorption spectra simulated using the different functionals and Wigner ensembles of 50 geometries around the minimum of conformer **IV** are shown in Figure S11a and S11b alongside the experimental absorption spectrum measured in THF. As can be seen, the experimental spectrum displays a double peak 3.85 and 3.97 eV (marked as B and C), that is preceded by a shoulder at 3.6 eV (marked as A). Compared to the experimental reference, the long-range corrected functionals, CAM-B3LYP, LC-BLYP and LC-BLYP*, as well as M06-2X show one broad blue-shifted absorption band.

Herein, the LC-BLYP* functional yields the best agreement with an approximate difference of 0.5 eV. The two hybrid functionals B3LYP and PBE0 show a red-shifted absorption band compared to the experimental band. PBE0 comes closest to the experimental values with a deviation of 0.35 eV. In addition, the absorption band obtained from PBE0 computations is broader and shows a small shoulder after its peak (marked with * in Fig. 11b), similar to the split band of the experiment. For this reason, we decided to use the PBE0 functional in the following calculations of the absorption spectrum and excited states of **1**$^+$.

To save computational costs, the comparison among functionals was first done for ensembles of 50 geometries only around the minimum of the lowest-energy conformer **IV** for the different functionals. Yet, as we show for one example below, the electronic structure of the excited states in the four conformers are similar; thus, it justifies that focusing on one minimum should give a good representation of the performance of the different functionals.

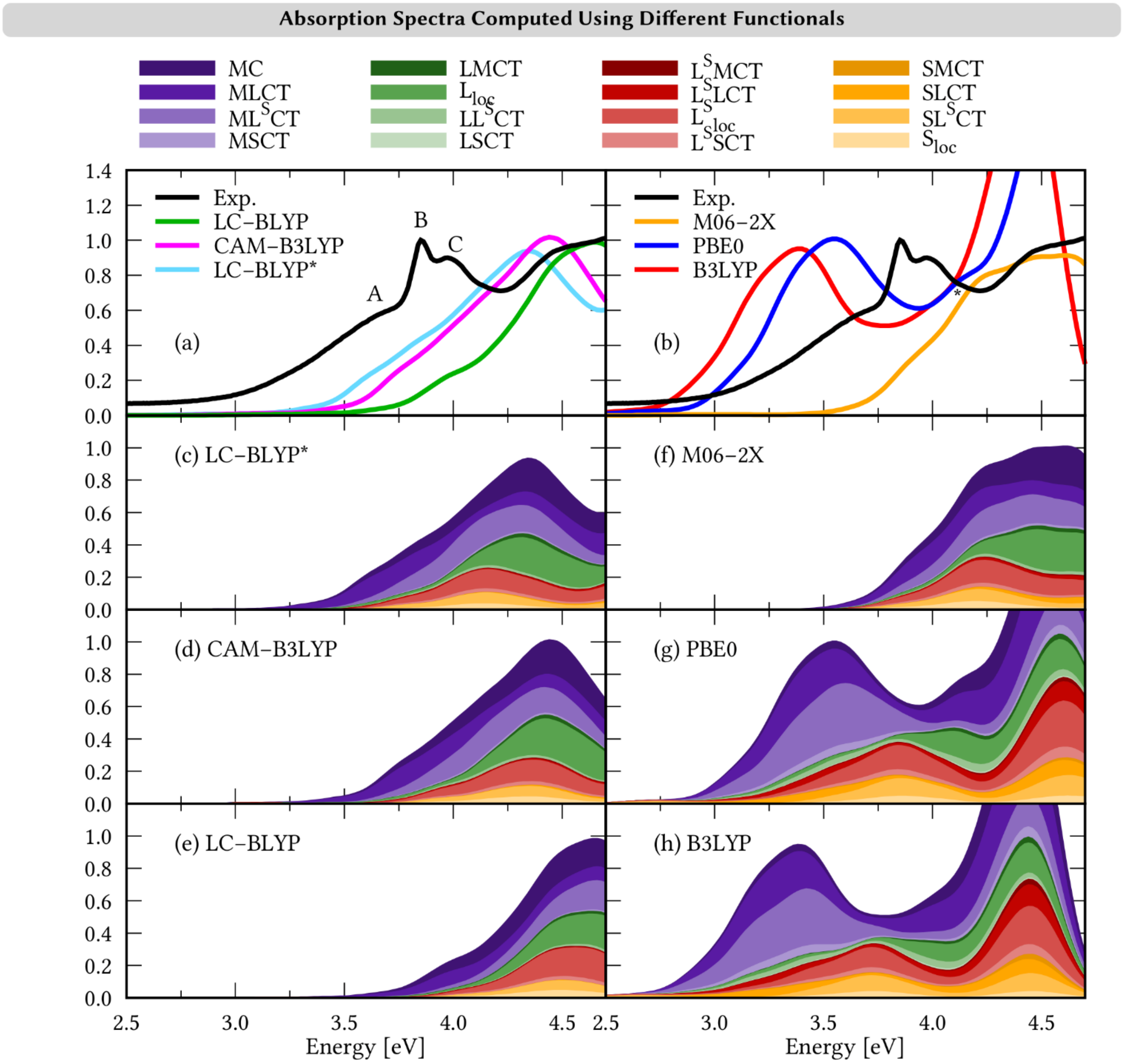

Figure S11. (a/b) Absorption spectrum of conformer **IV** computed using different functionals. (c-h) Transition-density matrix analysis of the different absorption spectra. The charge-transfer characters follow the fragmentation scheme shown in Figure 7b of the main manuscript.

Furthermore, a transition-density matrix analysis (see Section 2.4 for more details) was performed to analyse the spectra computed with the different functionals. As can be seen, the first absorption band for all functionals is dominated by excitations originating at the $Re(CO)_3$ moiety (fragment M, purple curves), i.e., metal-centered excitations (MC), metal-to-2,2'-bpy charge-transfer excitations (MLCT) and metal-to-$^{S\text{-}S}$byp^{4,4} charge-transfer excitations (ML^{S}CT). All functionals also show smaller contributions of excitations originating at the $^{S\text{-}S}$byp^{4,4} ligand, that is here fragmented into the aromatic core (fragment L^S, red curves) as well as the S-S bridge (fragment S, orange curves). These excitations are mainly comprised of localized excitations at the two fragments of the full $^{S\text{-}S}$bpy^{4,4} (L$^S_{loc}$, S_{loc}) or charge-transfer excitations between these units (L^{S}SCT, SL^{S}CT). Finally, the spectra computed using the different functionals differ in their contribution of excitations originating at the 2,2'-bpy ligand (L, green curves). These contributions are mainly excitations localized at the 2,2'-bpy ligand (L_{loc}). At the first absorption band, they are larger for the range-separated functionals LC-BLYP*, CAM-B3LYP, and LC-BLYP as well as the double-

hybrid M06-2X functionals compared to the hybrid PBE0 and B3LYP functionals. However, the large contribution from MC states is not typical for heavy transition metals with strong ligand field-splitting and are hence disregarded.

In Figure S12 we show the experimental absorption spectra of the free ligands bpy and $^{S\text{-}S}$bpy^{4,4}. As can be seen, the $^{S\text{-}S}$byp^{4,4} ligand exhibits an absorption band with a maximum at 320 nm (3.87 eV), while that of 2,2'-bpy lies at higher energies at 280 nm (4.42 eV). In the complex **[1]**$^+$, excitation localized at the 2,2'-bpy and $^{S\text{-}S}$bpy^{4,4} ligands should be expected to follow the same behavior with that of the $^{S\text{-}S}$byp^{4,4} ligand appearing at lower energies before localized 2,2'-bpy excitations. This behavior is followed best by the PBE0 functional (and the B3LYP functional) confirming further our choice of the PBE0 functional for the remaining of this work.

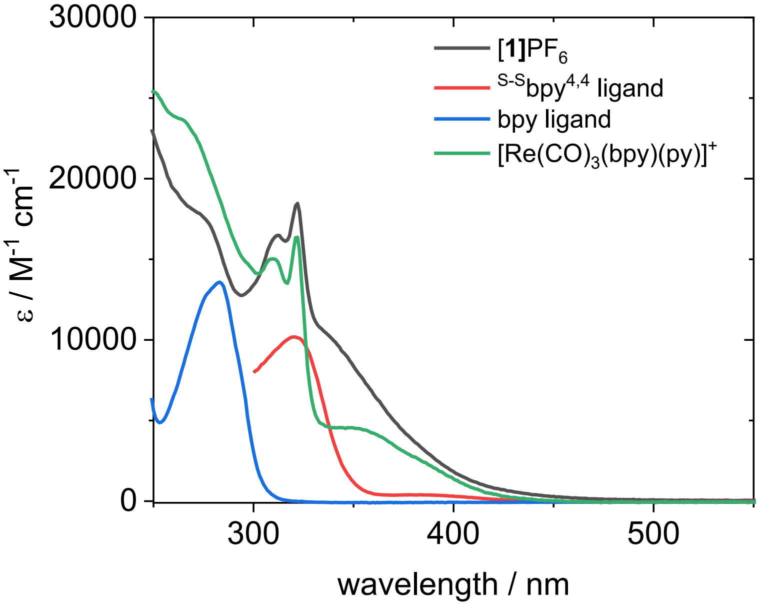

Figure S12. Absorption spectra of [**1**]$^+$ (black), as well as the free ligands bpy (blue) and $^{S\text{-}S}$byp^{4,4} (red), and the reference complex [Re(CO)$_3$(bpy)(py)]$^+$ (green) in THF.

Figure S13 shows the absorption spectra of the different conformers as well as their contributions to the full absorption spectrum when weighted by their Boltzmann populations at T = 300 K. As can be seen, the spectra of all four conformers are very similar, in terms of shape and position of the maxima. Thus, testing the performance of all functionals around the minimum of only one of the conformers is reasonable.

Absorption Spectra of the four Confomers

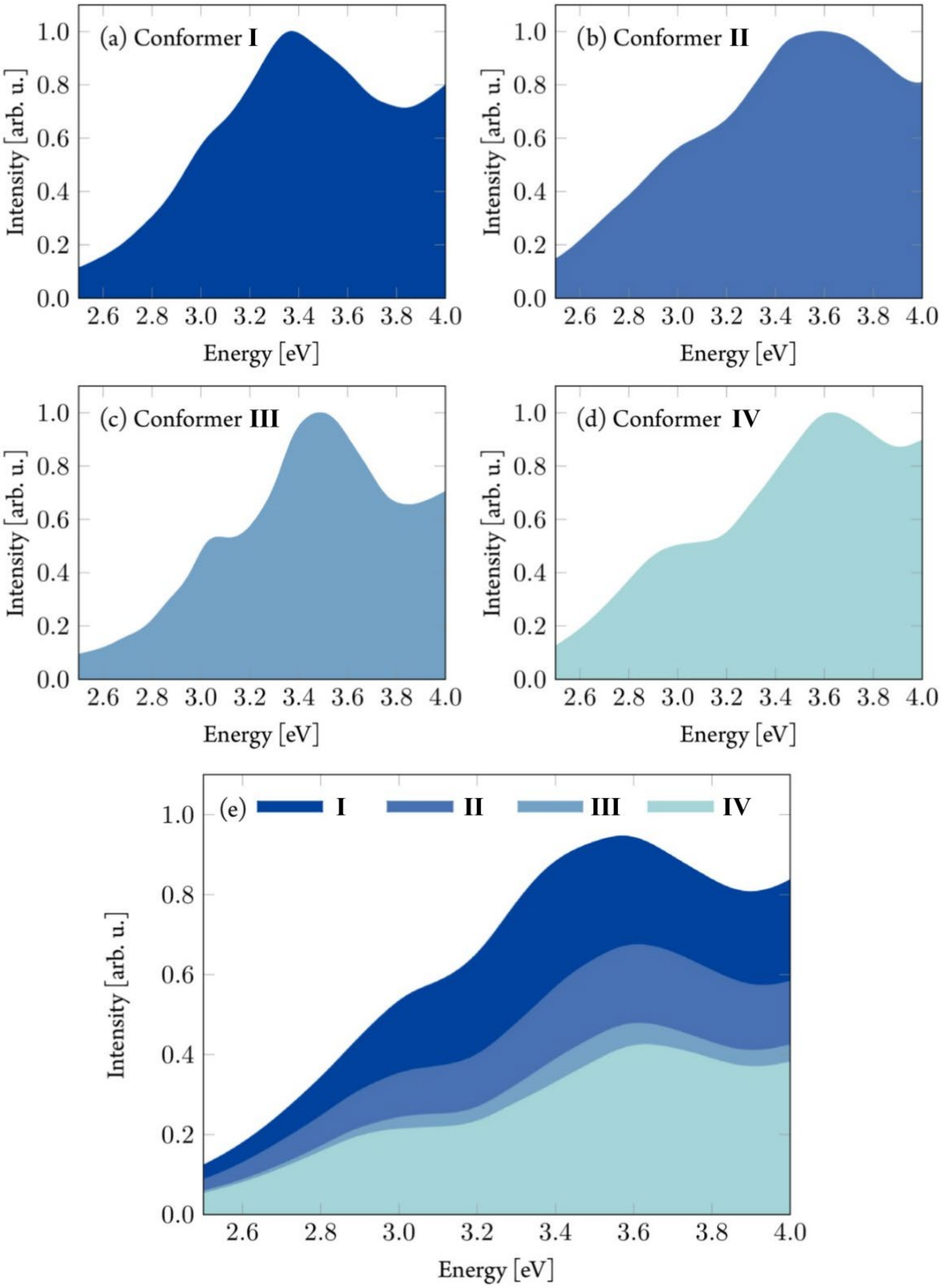

Figure S13. (a-d) Absorption spectra of the four different conformers (**I-IV**) computed using PBE0/TZVP. (e) Contributions of the four different conformers to the absorption spectrum. Conformers weighted according to their Boltzmann population at T = 300K.

S2.3 Influence of Solvent and of Spin-Orbit Coupling on the Absorption Spectrum

Here we investigated the influence of the solvent and inclusion of spin-orbit coupling on the absorption spectrum. As can be seen in Figure S14(a), including the solvent effects of THF via the SMD solvent model red-shifts the spectrum simulated at the PBE0/TZVP level of theory. The maximum of the first absorption band computed using the SMD solvent model now lies at 3.15 eV. This actually makes the agreement of the spectrum computed using the SMD solvent model with the experimental spectrum worse than for the spectrum computed in gas phase, despite the spectrum also being recorded in THF solution. Since the goal of our study is to simulate the excited-state dynamics of $[\mathbf{1}]^+$ this behavior is still acceptable for two reasons. First, performing the dynamics simulation using SHARC and TDDFT in the SHARC/ORCA4.2 interface is only possible in the gas phase, since ORCA4.2 does not provide excited-state gradients in the presence of a solvent model. Thus, we can take advantage of the apparent error cancellation that occurs when using the PBE0 functional and computing the excited states in the gas phase. Second, the shape of the experimental spectrum is well reproduced by the computed gas phase spectrum. While the computed gas-phase spectrum may only show a single broad peak instead of the double maxima visible in the experiment, it does reproduce the pre-band shoulder apparent in the experimental spectrum, signaling a description of the excited states, that yields somewhat underestimated energies but seems consistent across the investigated energy range.

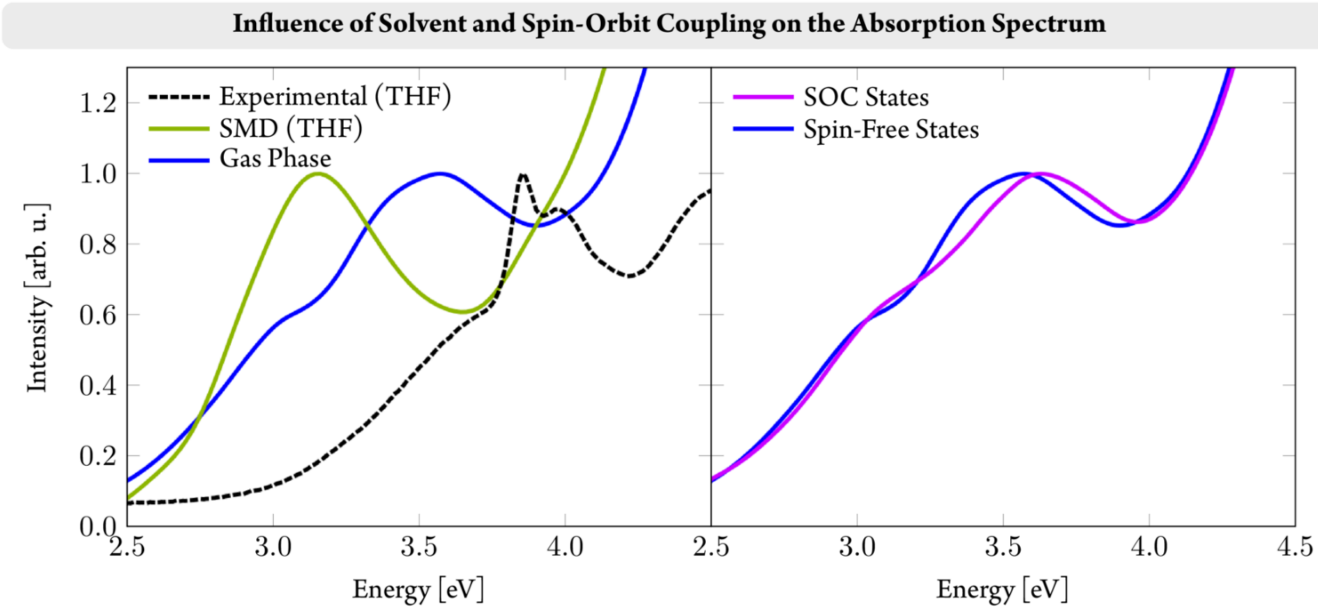

Figure S14. (a) Investigation of the influence of solvent effects of THF modelled by the SMD solvent model. (b) Investigation of the effect of including spin-orbit coupling when simulating the absorption spectrum.

The influence of spin-orbit coupling on the absorption spectrum is shown in Figure S14b, comparing the absorption spectrum in the absence (spin-free states) and presence (SOC states) of spin-orbit coupling. As can be seen, the absorption spectra resulting from both approaches are very similar. Thus, SOC does not seem to be responsible for any of the prominent features in the absorption spectrum such as the pre-band shoulder or the double maxima of the first absorption band (only seen in experiment). While the results including SOCs are more accurate, in the main paper we only show the results of the spin-free states, since only in this representation the transition-density matrix analysis is currently possible with our available software.

S2.4 Transition-Density Matrix Analysis

Electronically excited states are often characterized in terms of the canonical orbitals that characterize the most important configurations in the excited-state wave function. This characterization can become tedious in case many different electronic states have to be analyzed, and its results can be misleading when the excited-state wave functions have multiple configurations with non-negligible contributions. A more comprehensible description that also yields quantitative features of the excited states can be obtained from analyzing the transition density matrix.[19] For electronic structure methods that describe excited states only in the basis of singly excited configurations such as TDDFT, the one-particle density matrix contains the complete information on the excited state. Using a singular-value decomposition, the transition-density matrix provides a set of hole and electron natural transition orbitals (NTOs) that typically represent ca. 99% of the excitation character. When analyzing multiple geometries where it is not possible to compute meaningful averaged NTOs in a straightforward manner, this analysis can be further simplified. The molecule can be divided into several fragments and the contributions of the hole and electron parts to the full transition-density matrix can be computed. In this way, the excited-state wave function can be described in terms of contributions of excitations localized in each fragment and excitations of charge-transfer character between each pair of fragments. The amount of all contributions of the same character are then combined into charge-transfer number.[20]

For [**1**]$^+$ we initially performed this analysis by dividing the complex into five fragments: the rhenium metal center (M), the three carbonyl ligands (L^{CO}), the equatorial bipyridyl ligand (L), and the two separated fragments of the aromatic core of the axial $^{S\text{-}S}$bpy^{4,4} ligand (L^S) as well as the S-S bridge (fragment S) as shown in Figure S15(a). This fragmentation defines 25 distinct excitation characters (Figure S15(b)), and we show the different contributions of these excitation types to the absorption spectrum in Figure S15(c). The different CT contributions of the states in the absorption spectrum can be summed up to contributions that share the same donor fragment ("hole") and ones that share the same acceptor fragment ("electron"). For each fragment we can then calculate the electron-hole difference between the hole and electron parts in order to quantify the amount of charge that is transferred upon excitation. For the five fragments defined above, this analysis is shown in Figure S15(d). In this representation, we can see easily that the rhenium metal center and the three CO ligands behave very similar throughout the whole energy range covered in our spectrum: both fragments show a negative electron-hole difference population that varies unisono with the energy. Thus, in the excited states populated in the absorption spectrum, charge flow occurs simultaneously and in proportional amounts from the rhenium metal center and the three carbonyl ligands to the bpy and $^{S\text{-}S}$bpy^{4,4} ligands. Thus, we have combined the rhenium metal center the CO ligands to one fragment, and use the corresponding analysis including only four fragments in the main paper.

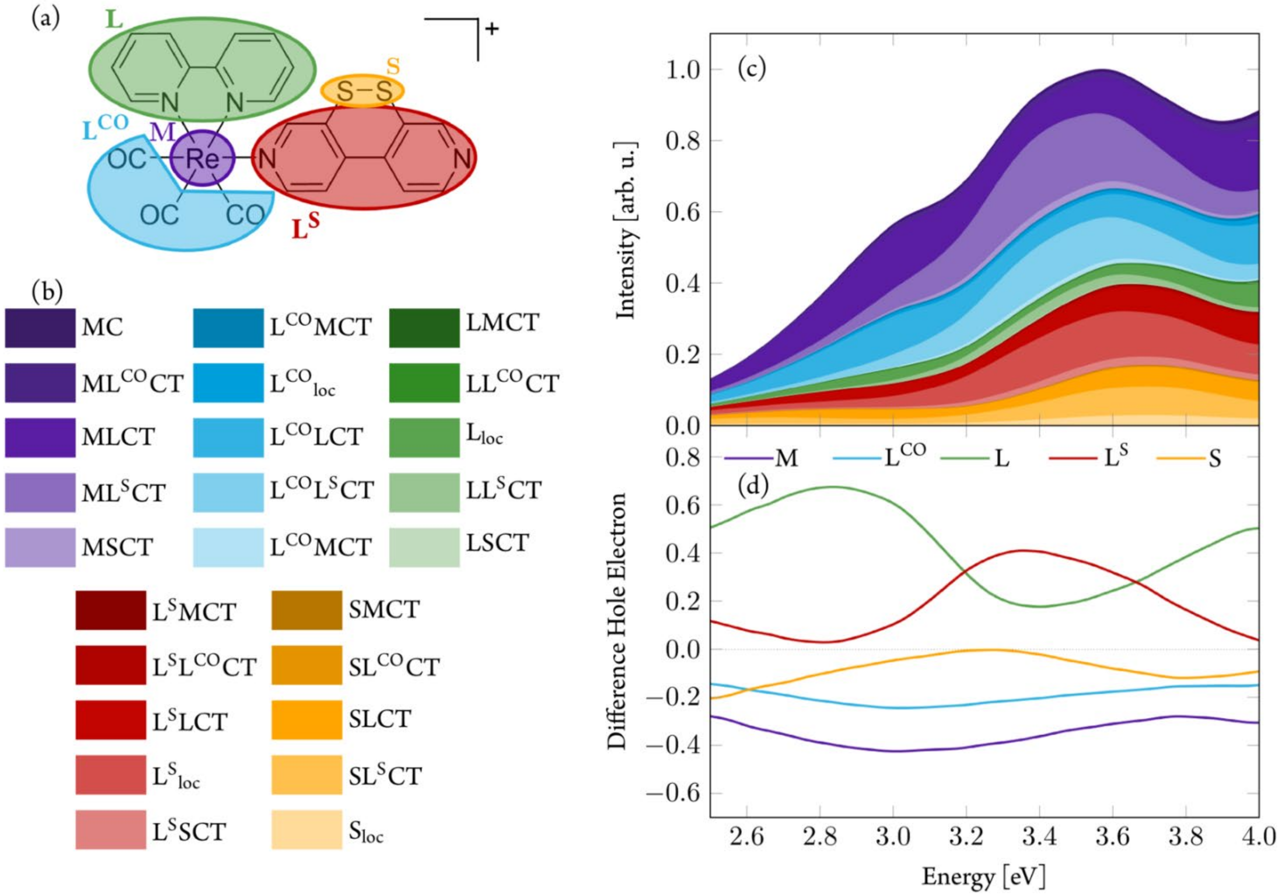

Figure S15. (a) Fragmentation of the complex. (b) Definition of distinct excitation characters. (c) Contribution to the absorption spectrum. (d) Electron-hole difference population of the excited states.

S3 Triplet Optimization

S3.1 Computational Details

Excited-state geometry optimizations were carried out for the T_1 state using the PBE0 functional and double zeta quality basis sets employing the ORCA4.2 program. Other computational parameters were used as described in Section S2.1. The presence of true minima of the optimized structures was confirmed through computing frequencies from the Hessian matrix. Geometry optimizations were started from the optimized ground-state geometry as well as from snapshots from the surface hopping dynamics as described in Section S7.4. For the minima referred to as T_1^{SSlong}, single-point calculations with other density functionals were performed as listed in Section S3.2

S3.2 Comparison of Different Triplet Energies

Based on the PBE0-optimized T_1^{SSlong} geometry, single-point calculations of the T_1 excitation energy were performed with a number of different density functionals. The results are reported in Table S1.

Table S1. T_1 excitation energies computed with different density functionals

Functional	T_1 Excitation Energy	Functional	T_1 Excitation Energy
PBE0	0.55 eV	PBE	0.56 eV
B3LYP	0.62 eV	CAM-B3LYP	0.71 eV
LC-BLYP	0.75 eV	LC-BLYP*	0.67 eV
ωB97X-V	0.90 eV	M06-2X	0.64 eV
Experiment	2.16 eV		

S4 TDDFT Surface Hopping Dynamics

S4.1 Computational details

The non-adiabatic dynamics of [**1**]$^+$ have been simulated using surface hopping[21] with the SHARC program package.[22] Electronic structure calculations were performed at the TDDFT level of theory, using the PBE0 functional, the SARC-ZORA-SVP[5] basis set for Re and the ZORA-def2-SVP[6] basis sets for the other elements (C, O, N, S, H). Other TDDFT parameters were kept as described in Section S2.1.

Initial coordinates and momenta for the dynamics were selected from a Wigner ensemble[16] of the most stable conformer IV of [**1**]$^+$ generated at a temperature of T=300 K.[17] Initial electronic states were chosen stochastically based on their oscillator strength[23] in an excitation energy window of 2.70-3.20 eV.

The different character of the electronic states with electron transfer from the $Re(CO)_3$ fragment either to the bpy or the $^{S\text{-}S}bpy^{4,4}$ ligand suggested the possibility of different dynamics dependent on excitation wavelength. However, since our time-resolved experiments showed no such behavior, simulations were started only in one energy region. Previously, we also reported nonadiabatic dynamics simulations of a related complex, $[Ru(bpy)_2(^{S\text{-}S}bpy^{2,2})]^{2+}$ ($^{S\text{-}S}bpy^{2,2}$ = [1,2]Dithiino[4,3-b:5,6-b′]dipyridine).[24] This complex also showed distinct energy regions to excite in either the bpy or the $^{S\text{-}S}bpy^{2,2}$ ligands. However, both excitations lead to rapid intersystem crossing and relaxation to the same triplet state after only ca. 250 fs.

First, 101 trajectories were simulated for a simulation time of 100 fs. The trajectories were propagated for 100 fs with a nuclear time step of 0.5 fs and an electronic time step of 0.02 fs. Due to statistical anomalies, 5 trajectories were excluded from the analysis (see below). An amount of 60 randomly selected trajectories were propagated for an additional 50 fs (150 fs total). Two example trajectories were propagated for a total of 200 fs.

An energy-based decoherence correction with a constant value of 0.1 a.u. was employed.[25] After a surface hop, the kinetic energy was adjusted by rescaling the velocity vectors. The surface hopping probabilities were computed based on wave-function overlaps.[26] A gradient selection was performed to speed up the computations, i.e., only gradients of states within ±0.5 eV of the active states were computed at each time step. This led to the computation of roughly 20 gradients at each time step. Including all 61 gradients would be computationally much more expensive, increasing the wall-clock time of a single time step by roughly 1 hour. Including only a selected amount of gradients however, allows hops only to states where the gradient was calculated.

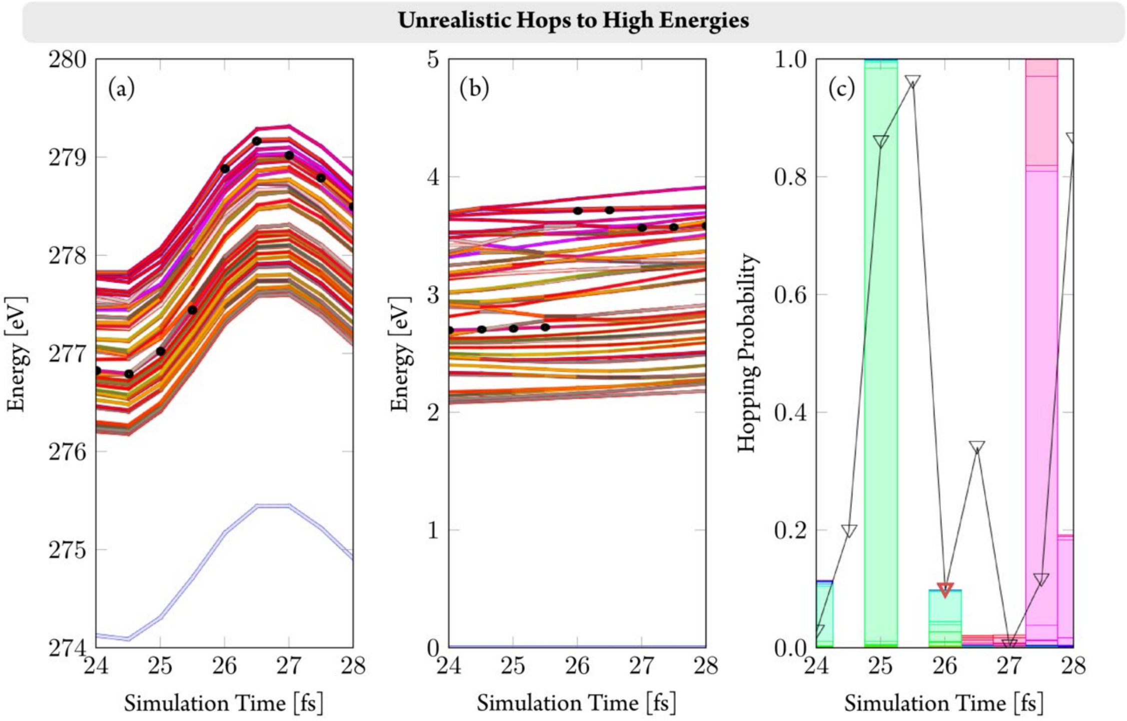

Figure S16. (a) Adiabatic states, the circle represents the active state. (b) Diagonal states, the circle represents the active state. (c) Hopping probabilities for the various adiabatic states, the triangle represents the random number.

Five trajectories were excluded from the statistical analysis due to the unrealistic occurrence of hops to high energies. One example is shown in Figure S13, with a hop at t=26 fs. Figure S16(a) shows the singlet and triplet states in the adiabatic representation, the active state is indicated by a circle. The diagonal states are shown in Figure S16(b). The hopping probability is seen in Figure S16(c), color coded for the various states. The drawn random number is indicated by a triangle. The probability of the chosen state is shown in blue and is roughly 0.0007%. Such hops with small probabilities are supposed to occur only rarely in a large sample size of trajectories. With a small sample size like as used in this study (101 trajectories) such paths would be over-represented and are thus excluded from analysis.

S4.2 Hops in the Short-Time TDDFT Nonadiabatic Dynamics

In order to establish the mechanism describing the time evolution of the electronic state populations, we have analyzed the number of hops between adiabatic electronic states during the surface hopping simulations. For this, we show the difference transition matrix in Table S2, that only shows the net number of hops for the 100 fs simulation time.

Table S2. Difference transition matrix of the 100 fs TDDFT surface hopping dynamics in the adiabatic representation. Row denotes initial state, column denotes final state.

	S_0	S_1	S_2	S_3	4	S_5	S_6	S_7	S_8	S_9	S_{10}	S_{11}	S_{12}	S_{13}	S_{14}	S_{15}	T_1	T_2	T_3	T_4	T_5	T_6	T_7	T_8	T_9	T_{10}	T_{11}	T_{12}	T_{13}	T_{14}	T_{15}	Sum
S_0	0	0	0	0	0	0	0	0	0	0	0	0	0	0	0	0	0	0	0	0	0	0	0	0	0	0	0	0	0	0	0	0
S_1	0	0	12	2	1	2	0	0	0	0	0	0	0	0	0	0	-1	-1	-6	-2	1	0	1	0	0	0	0	0	0	0	0	9
S_2	0	-12	0	13	10	1	0	0	0	0	0	0	0	0	0	0	0	-2	-7	5	-4	-1	1	0	0	0	0	0	0	0	0	4
S_3	0	-2	-13	0	8	4	0	0	0	-1	0	0	0	-1	0	0	0	0	-5	0	-4	0	3	-2	1	0	0	0	0	0	0	-12
S_4	0	-1	-10	-8	0	13	0	1	0	0	0	0	0	0	0	0	0	0	0	0	6	-15	-7	-1	2	0	0	-1	0	0	0	-21
S_5	0	-2	-1	-4	-13	0	3	1	-1	0	0	0	0	0	0	0	0	0	0	0	-4	-7	2	-1	-1	-3	0	0	0	0	0	-31
S_6	0	0	0	0	0	-3	0	-4	3	-1	0	0	0	0	0	0	0	0	0	0	0	1	-3	0	3	0	-2	2	0	0	0	-4
S_7	0	0	0	0	-1	-1	4	0	1	2	-1	0	0	0	0	0	0	0	0	0	0	0	-1	-2	-1	0	1	-1	1	-1	0	0
S_8	0	0	0	0	0	1	-3	-1	0	-2	0	0	0	-1	0	0	0	0	0	0	0	0	0	0	-1	2	-1	0	0	1	1	-4
S_9	0	0	0	1	0	0	1	-2	2	0	6	2	-1	-1	0	0	0	0	0	0	0	0	0	0	0	-1	2	-2	-3	-1	-3	0
S_{10}	0	0	0	0	0	0	0	1	0	-6	0	2	0	0	0	0	0	0	0	0	0	0	0	0	0	0	0	0	2	-1	2	0
S_{11}	0	0	0	0	0	0	0	0	0	-2	-2	0	2	1	0	0	0	0	0	0	0	0	0	0	0	0	0	0	0	0	1	0
S_{12}	0	0	0	0	0	0	0	0	0	1	0	-2	0	2	0	0	0	0	0	0	0	0	0	0	0	0	0	0	0	-1	0	0
S_{13}	0	0	0	1	0	0	0	0	1	1	0	-1	-2	0	-2	2	0	0	0	0	0	0	0	0	0	0	0	0	0	0	0	0
S_{14}	0	0	0	0	0	0	0	0	0	0	0	0	0	2	0	-1	0	0	0	0	0	0	0	0	0	0	0	0	0	-1	0	0
S_{15}	0	0	0	0	0	0	0	0	0	0	0	0	0	-2	1	0	0	0	0	0	0	0	0	0	0	0	0	0	0	0	1	0
T_1	0	1	0	0	0	0	0	0	0	0	0	0	0	0	0	0	0	18	1	1	0	0	0	0	0	0	0	0	0	0	0	21
T_2	0	1	2	0	0	0	0	0	0	0	0	0	0	0	0	0	-18	0	14	8	0	0	0	0	0	0	0	0	0	0	0	7
T_3	0	6	7	5	0	0	0	0	0	0	0	0	0	0	0	0	-1	-14	0	8	-3	1	0	0	0	0	0	0	0	0	0	9
T_4	0	2	-5	0	0	0	0	0	0	0	0	0	0	0	0	0	-1	-8	-8	0	21	6	0	0	0	0	0	0	0	0	0	7
T_5	0	-1	4	4	-6	4	0	0	0	0	0	0	0	0	0	0	0	0	3	-21	0	16	2	0	0	0	0	0	0	0	-1	4
T_6	0	0	1	0	15	7	-1	0	0	0	0	0	0	0	0	0	0	0	-1	-6	-16	0	1	0	1	0	0	0	0	0	0	1
T_7	0	-1	-1	-3	7	-2	3	1	0	0	0	0	0	0	0	0	0	0	0	0	-2	-1	0	1	0	-1	-1	0	0	0	0	0
T_8	0	0	0	2	1	1	0	2	0	0	0	0	0	0	0	0	0	0	0	0	0	0	-1	0	-1	-2	1	0	0	0	0	3
T_9	0	0	0	-1	-2	1	-3	1	1	0	0	0	0	0	0	0	0	0	0	0	0	-1	0	1	0	-1	5	-1	0	1	0	1
T_{10}	0	0	0	0	0	3	0	0	-2	1	0	0	0	0	0	0	0	0	0	0	0	0	1	2	1	0	-8	1	1	0	0	0
T_{11}	0	0	0	0	0	0	2	-1	1	-2	0	0	0	0	0	0	0	0	0	0	0	0	1	-1	-5	8	0	1	1	-2	0	3
T_{12}	0	0	0	0	1	0	-2	1	0	2	0	0	0	0	0	0	0	0	0	0	0	0	0	0	1	-1	-1	0	1	2	-2	2
T_{13}	0	0	0	0	0	0	0	-1	0	3	-2	0	0	0	0	0	0	0	0	0	0	0	0	0	0	-1	-1	-1	0	4	0	1
T_{14}	0	0	0	0	0	0	0	1	-1	1	1	0	1	0	1	0	0	0	0	0	0	0	0	0	-1	0	2	-2	-4	0	1	0
T_{15}	0	0	0	0	0	0	0	0	-1	3	-2	-1	0	0	0	-1	0	0	0	0	1	0	0	0	0	0	0	2	0	-1	0	0
Sum	0	-9	-4	12	21	31	4	0	4	0	0	0	0	0	0	0	-21	-7	-9	-7	-4	-1	0	-3	-1	0	-3	-2	-1	0	0	0

S4.3 Note on the Rate of Intersystem Crossing in the Simulation

In the main paper, the mechanism of the short-time TDDFT nonadiabatic dynamics simulations included fitted time constants for singlet-to-triplet intersystem crossing τ_{ISC}=25±3 fs as well as triplet-to-singlet back intersystem crossing of τ_{bISC}=69±17 fs. The initial as well as the back-ISC are partially due to representation of the electronic states. Due to the presence of spin-orbit coupling, trajectories are never in pure singlet or triplet states during the simulation. Instead, the electronic wave function of each trajectory is described by a linear combination of all included electronic states.

To demonstrate this spin mixing, we can calculate the spin expectation value $\langle S^2 \rangle$ of each trajectory. For pure singlet states, $\langle S^2 \rangle$ would be zero, while for pure triplet states, it would amount to 2. At the beginning of the dynamics (t=0.0 fs), only 12 of the 96 trajectories exhibit $\langle S^2 \rangle$ of 0.0-0.2, i.e. being close to pure singlet states. For all remaining trajectories, $\langle S^2 \rangle$ is larger. Thus, the time constants from the mechanism obtained in this representation may not be directly comparable to experimentally observed ones. However, they still allow to establish the time scales of the relaxation dynamics.

S4.4 Evolution of S-S Bond Length of Trajectories Propagated for 200 fs

In order to investigate the behavior of trajectories at prolonged S−S distances, we propagated two trajectories which had already undergone pronounced S−S bond elongation after 150 fs for an additional 50 fs, i.e., up to a total simulation time of 200 fs. These two trajectories are shown in Figure S17. As can be seen, after reaching maximum S−S bond lengths of ca. 3.0 Å, the S−S bond length decreases again, and continues to oscillate around 2.6 Å, i.e., the bond length of the T_1^{SSlong} geometry.

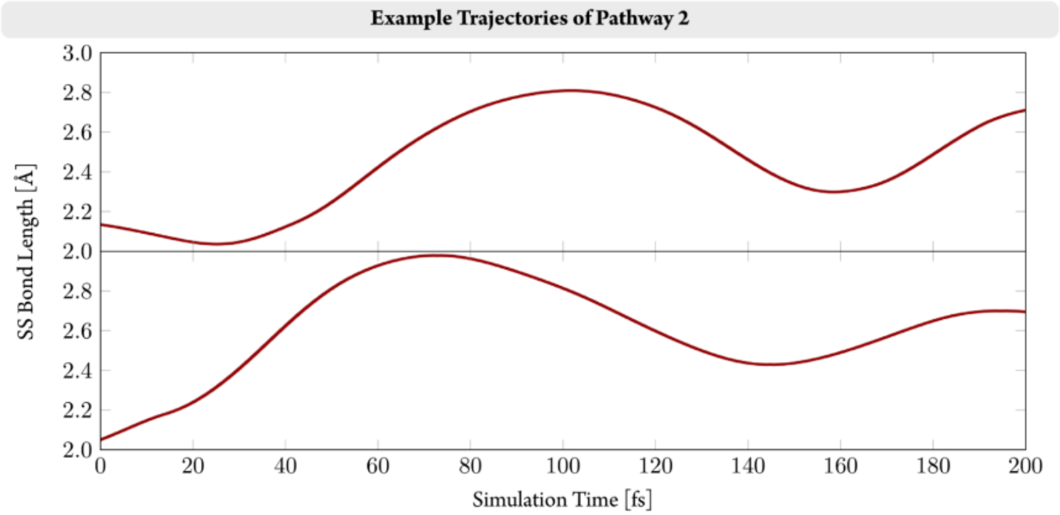

Figure S17. S−S bond length of two trajectories of pathway 2 propagated up to 200 fs.

S5 Potential Energy Scan Along the S-S Bond of the Ligand $^{S\text{-}S}$bpy^{4,4}

S5.1 Discussion

In our surface hopping dynamics simulations, two pathways of trajectories were observed. One pathway included pronounced elongations of the S−S bond length from starting values of ca. 2.1 Å close to the Franck-Condon geometry up to maximum values of ca. 3.1 Å. Upon extended simulation of some of these trajectories, it was observed that they return to bond lengths around 2.6 Å, i.e., close to the T_1^{SSlong} geometry.

This behavior of strong bond elongation can be worrisome for our simulations. It could indicate an attempt of the trajectories to dissociate which could be hindered by the disability of our electronic structure (TD)DFT method to describe. TDDFT uses DFT to compute the electronic ground state, which is a single reference method, i.e., it uses only one configuration –described by a single Slater determinant –for the electronic wave function.[27] This description is erroneous in describing homolytic bond breakage, where even a qualitatively correct ground-state wave function requires several electronic configurations.

While the TDDFT excited-state wave functions are linear combinations of multiple configurations, these configurations are built from the orbitals of the ground-state DFT wave function. TDDFT is consequently only able to give an appropriate description of the excited states of a system, if its ground state is likewise well described by DFT. Thus, the description of processes occurring in the excited states using TDDFT also requires that an adequate description of the ground state using DFT is possible at all geometries that are traversed.

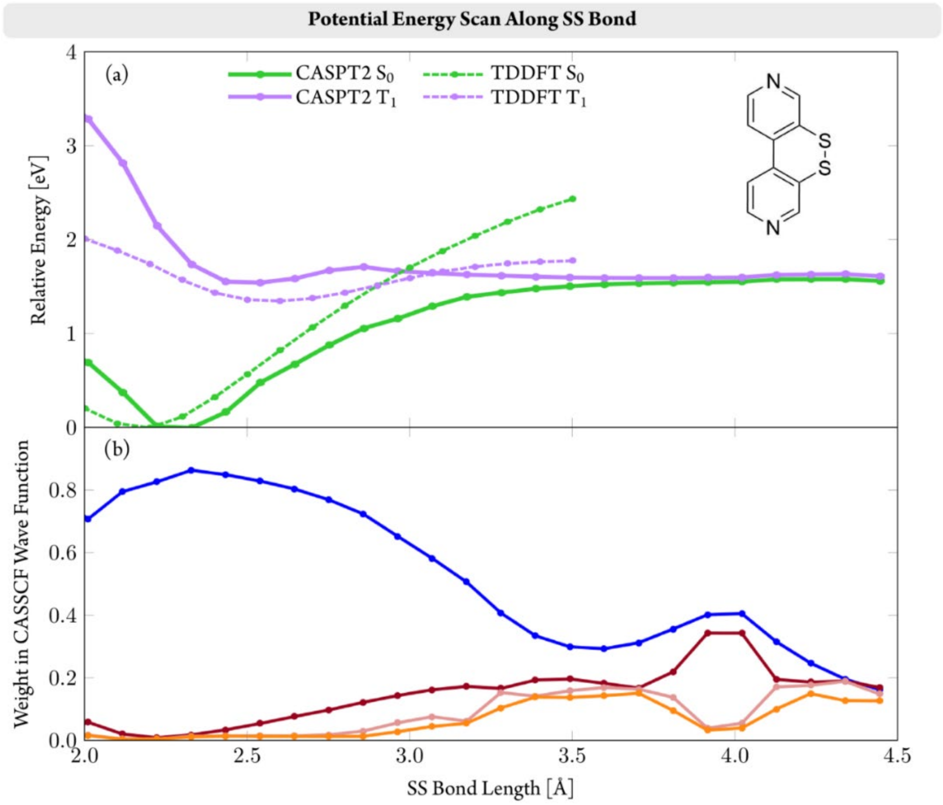

Figure S18. Relaxed scan in the lowest triplet state T_1 along the S-S bond in the isolated ligands $^{S\text{-}S}bpy^{4,4}$. (a) Comparison of TDDFT and CASPT2 energies of the S_0 and T_1 states in eV. (b) Weight of the four most important configurations in the CASSCF wavefunction of the S_0. The blue curve always refers to the "non-excited" closed-shell configuration, while other curves are only selected according to their order in the weights.

To test this, we have performed a relaxed potential energy scan in the lowest triplet state T_1 along the elongation of the S−S bond using our TDDFT setup from the simulations and using the multi-reference methods CASSCF/CASPT2.[28] Due to the large computational cost of CASSCF/CASPT2 for molecules the size of the $\mathbf{1}^+$, we performed the scan only for the isolated $^{S\text{-}S}bpy^{4,4}$ ligand. Computational details for this section are reported in Section S5.2.

While the restriction to the $^{S\text{-}S}bpy^{4,4}$ ligand was motivated to avoid huge computational costs, it should also be sufficient for our given test. The motion of pathway 2 trajectories is nearly completely described by motion of the atoms in the $^{S\text{-}S}bpy^{4,4}$ ligand. Furthermore, once in T_1^{SSlong}-like states, there is little influence of electronic excitation involving other fragments of the complex besides the aromatic ring of the $^{S\text{-}S}bpy^{4,4}$ ligand and its S−S unit, as shown in Figure 8(c) in the main paper. We emphasize this point: the T_1^{SSlong}-like states are truly localized excitations. This is a quantitative assessment, and this statement is possible as our transition-density matrix analysis captures the full excited-state wave functions of our TDDFT results (within the employed Tamm-Dancoff approximation). Thus, the results of comparing the performance of TDDFT and CASSCF/CASPT2 for the S−S bond elongation of the isolated $^{S\text{-}S}bpy^{4,4}$ ligand should be transferable to the corresponding S−S bond elongation in $\mathbf{1}^+$.

The potential energy curves of the S_0 and T_1 of a relaxed scan in the T_1 state along the S−S bond elongation are shown in Figure S18(a). The CASPT2 curve shows a minimum for the T_1 at a S−S bong length ca. at 2.5 Å, while full optimization of the T_1 at the CASSCF level results in 2.56 Å. Similarly, TDDFT optimization finds a T_1 minimum of the $^{S\text{-}S}bpy^{4,4}$ ligand at 2.57 Å, i.e., at the same length as the the T_1^{SSlong} minimum of the full $\mathbf{1}^+$. In further accordance, TDDFT predicts the same S_0/T_1 energy gap of 0.56 eV at the corresponding T_1 minima of isolated $^{S\text{-}S}bpy^{4,4}$ ligand and the complex. This gap is 0.88 eV in CASPT2

when using an IPEA shift of 0.25 a.u.,[29] however it decreases to 0.58 eV when removing the IPEA shift.[30] Despite the S_0/T_1 gap at this geometry being smaller without no IPEA shift, the overall agreement between CASPT2 and TDDFT curves is better when using the IPEA shift (see Section S5.3).

At longer S−S bond lengths than the T_1 minimum, the TDDFT and CASPT2 curves first increase simultaneously in energy until distances of ca. 2.95 Å. At this distance, a local maximum in the CASPT2 curve of the T_1 state is reached, while the TDDFT energy of the T_1 state continues to rise with increasing S−S bond length. In the CASPT2 picture, the T_1 becomes (nearly) degenerate with the S_0 at distances larger than 3.5 Å towards the dissociation limit. This signifies the possibility for the system to relax from the T_1 to the electronic ground state S_0 and subsequently return to its equilibrium geometry in competition to dissociation in the T_1 state.

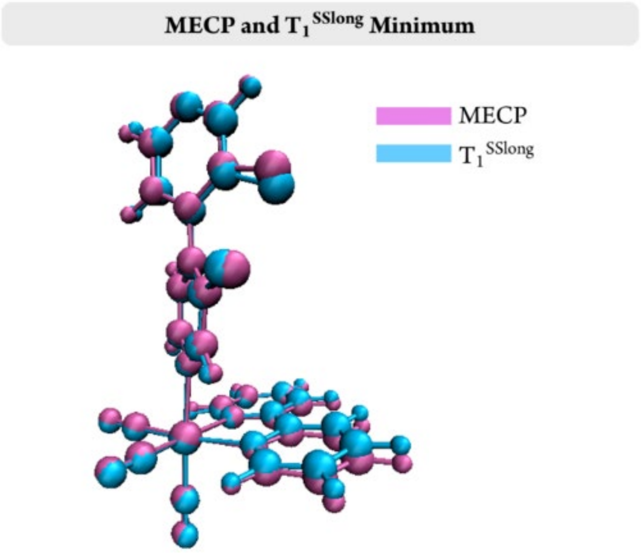

Figure S19. Superposition of geometries at the S_0/T_1 minimum-energy crossing point (MECP) and the T_1^{SSlong} minimum. The S−S bond lengths of the MECP and the T_1^{SSlong} minimum are 2.80 and 2.56 Å, respectively. The MECP lies 0.14 eV above the T_1^{SSlong} minimum.

Using TDDFT, there is crossing of the T_1 and S_0 states for the $^{S\text{-}S}bpy^{4,4}$ ligand around 2.9 Å. We note at this point that a similar S_0/T_1 crossing point can also be found for the [**1**]$^+$ complex, albeit at a slightly smaller distance of 2.80 Å (see Figure S19). The S_0/T_1 crossing point of [**1**]$^+$ lies only 0.14 eV above the T_1^{SSlong} minimum.

Beyond the S_0/T_1 crossing point, the T_1 becomes the lowest-energy state, both for the isolated $^{S-S}bpy^{4,4}$ ligand and for the [**1**]$^+$ complex. This behavior is already qualitatively wrong, due to the restriction of a single closed shell reference configuration in the (TD)DFT ansatz. During the excited-state dynamics simulation, we could also observe this reversed state ordering for some of the pathway 2 trajectories. An example of this behavior is shown in Figure S20. As can be seen, the trajectory leaves this problematic region after few dozens of femtoseconds. In principle, the trajectory should be able to follow the potential energy surface further towards dissociation in the triplet state (see Figure S18(a)). However, none of the trajectories actually follow this path too far, thus avoiding the most problematic regions in the TDDFT potential energy surfaces.

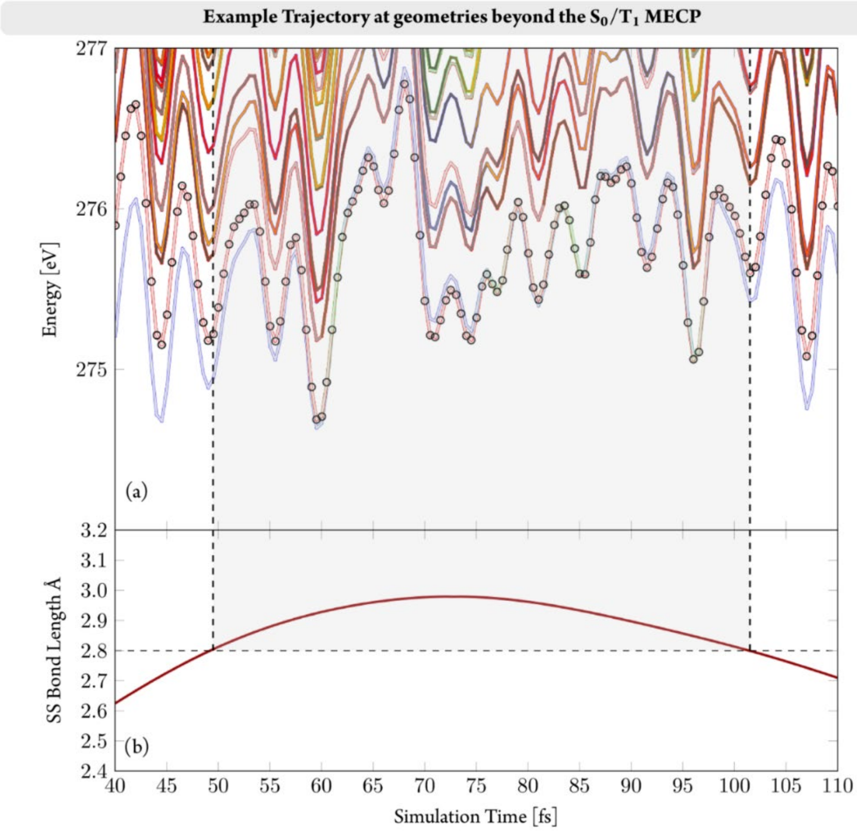

Figure S20. Example trajectory at geometries beyond the S_0/T_1 MECP. (a) Adiabatic states (not all shown). The active state is indicated by circles. In this case the active state is the T_1 throughout the shown simulation time. (b) The S-S bond length over the simulation time. The dashed line along 2.8 Å indicates the bond length of the S_0/T_1 MECP. The gray box depicts the region where the S-S bond length of the trajectory is beyond the MECP bond length. In this region, the S_0 (lowest blue curve) and T_1 (lowest red curve) become very close in energy, with the T_1 even being the lowest energy state. At bond lengths smaller than 2.8 Å the S_0 and T_1 show a more distinct separation.

It is difficult to define a clear point or region where the "problematic" territory of the TDDFT potential energy landscape begins solely by comparing the TDDFT energies with the CASSCF/CASPT2 ones. For molecules the size of $[\mathbf{1}]^+$ it is natural to expect certain quantitative energy differences between both electronic structure methods, and defining a threshold for when quantitively different results become qualitatively different is somewhat arbitrary. However, we can investigate (one of) the defining qualitative difference(s) between both approaches, i.e., the form of the ground-state wave function. For (TD)DFT, this wave function takes the form of a single configuration at each point while for CASSCF/CASPT2, it is a linear combination of multiple configurations with different weights at each point. Thus, we show in Figure S18(b) the weights of the four most important configuration in the ground-state CASSCF wave function.

As can be seen, throughout the whole range of S−S bong length, the most dominant configuration in the CASSCF wave function is the "non-excited" closed-shell configuration (blue curve). In this configuration, all π and σ/n orbitals are doubly occupied and all π* orbitals are empty. Certainly, choosing this configuration as the non-excited reference configuration in a CASSCF ansatz is arbitrary. However, this configuration resembles most closely the one from the (TD)DFT ground state. We will refer to this configuration as Φ_0.

We note that even close to the ground-state equilibrium geometry –at distances of ca. 2.1 Å –the weight of Φ_0 is only ca. 80% in the full CASSCF wave function. This is a typical behavior for CASSCF ground-state wave functions of larger molecules when an active space is used that contains already a larger number of orbitals (say: more than 2-4). This behavior can be associated with the (partial) description of

dynamical correlation energy, which can also be (partially) captured with single-reference methods such as DFT or Møller-Plesset perturbation theory.

Upon bond elongation, the weight of Φ_0 first increases to 90%, but then drops continuously until distances of 3.5 Å. Concomitant with the decrease, a few other configurations start to gain importance. Their individual weights are all below 10% for distances smaller than 2.75 Å, and they remain below 20% even for distances up to 3.5 Å. At a distance of 3.2 Å, the weight of Φ_0 has dropped to 50%, while the combined weight of the three next important configurations amounts to 25%. Thus, this distance of 3.2 Å is probably the latest point when we should assign a multi-reference character to the system based on its ground-state wave function. However, a more cautionary assignment could begin at distances as small as 2.75 Å.

The cautionary distance threshold of 2.75 Å is larger than that of the T_1^{SSlong} minimum (2.57 Å), signifying that this state can still be described very well with TDDFT. In contrast, this cautionary threshold is transgressed by the pathway 2 trajectories in the dynamics simulation. However, as these trajectories appear to oscillate around the T_1^{SSlong} minimum bong length, and only rarely reach more clearly problematic regions beyond 3.0 Å, this behavior should be acceptable.

S5.2 Computational Details

In order to investigate the behavior of the TDDFT setup, a relaxed scan in the lowest-energy triplet state T_1 along the S−S bond was performed using the TDDFT setup used in the surface hopping simulations and the multiconfigurational methods CASSCF/CASPT2[31] for the isolated $^{S\text{-}S}$bpy^{4,4} ligand. Geometry optimizations were performed for bond distances between 4.0 and 8.4 a.u. in spacings of 0.2 a.u.. For the TDDFT scan, the same parameters were used as in the dynamics (see Section S4.1), with the exception that only one root (the T_1) was included in the TDDFT geometry optimizations.

For the CASSCF/CASPT2 scan, geometries were optimized at the CASSCF level of theory, for which energies were calculated using CASPT2. All CASSCF/CASPT2 calculations were performed using OpenMolcas version 18.0[32] and the ANO-RCC-VDZP basis set for all elements.[33] An (12,12) active space of 12 orbitals and 12 electrons was used, for which exemplary orbitals computed at distances of 4.4 and 8.4 a.u. are shown in Figure S21. For integral computations, the resolution of identity technique with on-the-fly generated auxiliary basis sets based on the Cholesky decomposition (RICD) method was used.[34]

At each optimized geometry, a multi-state CASPT2 calculation were performed including 4 singlet and 4 triplet states. An imaginary level shift of 0.1 a.u. was used to avoid intruder state problems.[35] The influence of the IPEA shift[29][30] was tested by using shift values of 0 and 0.25 a.u.

In Section S5.1, only the potential energy curves for the singlet ground state S_0 and lowest excited triplet state T_1 obtained using the IPEA shift of 0.25 a.u. are shown. All other CASSCF/CASPT2 results are discussed in Section S5.3

In addition to the relaxed scan, minima of the T_1 state of the isolated $^{S\text{-}S}$bpy^{4,4} were optimized using both TDDFT and CASSCF.

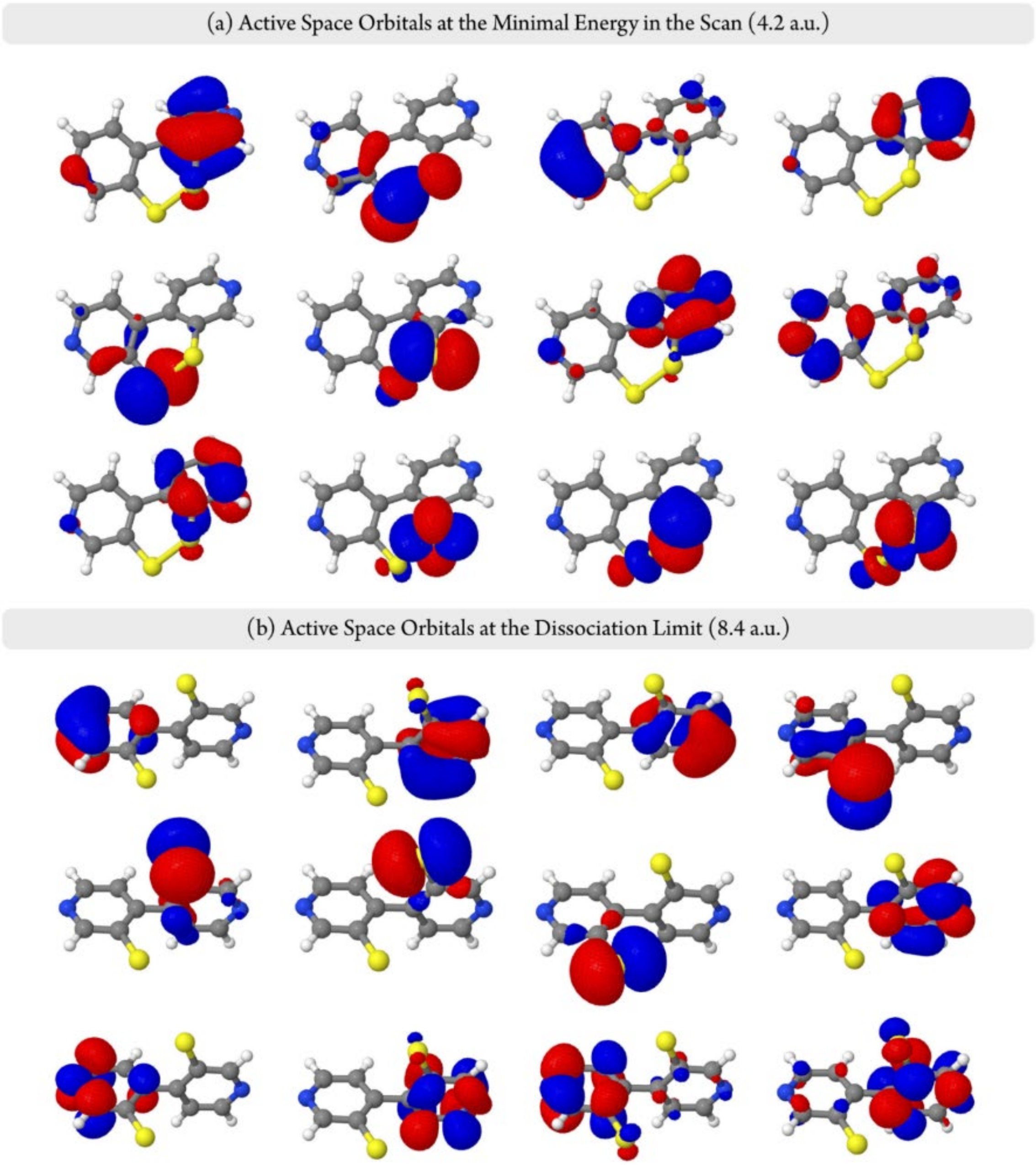

Figure S21. Orbitals in the (12,12) active space computed at two different geometries at 4.2 and 8.4 a.u.

S5.3 Influence of the IPEA Shift and State Mixing in CASSCF/CASPT2 Calculations

The IPEA shift has been introduced in CASPT2 to correct for an overestimation found in computing dissociation/atomization energies of small molecules[29,36] Recently, the role of using the IPEA shift to calculate excited states in organic molecules was questioned. Therefore, here we test the effect of including an IPEA shift of 0.25 a.u.[34] or setting it to zero. [30] The resulting potential energy curves are shown in Figure S22 for IPEA=0.25 a.u. (a) and IPEA=0 (b). Fortunately, there are only small differences between both sets of curves, and we decided to use the IPEA=0.25 a.u. curves in our discussion in Section S5.1.

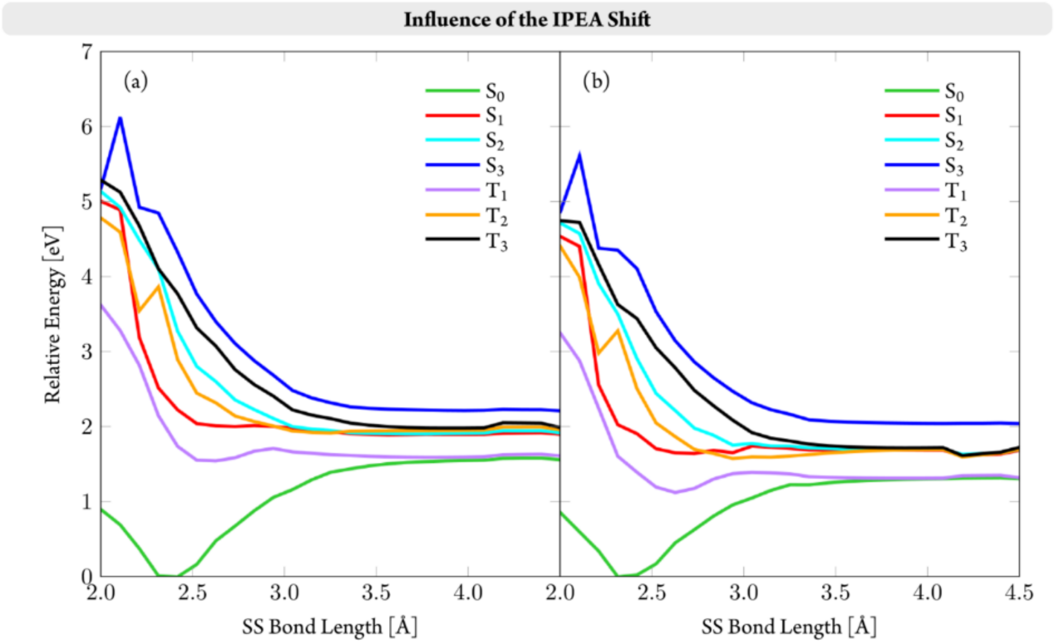

Figure S22. MS-CASPT2 curves computed using an IPEA shift of 0.25 a.u. (a) and 0 a.u. (b).

While we were only interested in the curves of the S_0 and T_1 states, we performed multi-state CASPT2 calculations including 4 singlet and 3 triplet states. This had to be done in order to allow for state mixing in the CASPT2 calculations that was necessary to obtain smooth potential energy curves. This is demonstrated in Figure S23, where we compare the CASSCF curves (a) with the MS-CASPT2 curves from the IPEA=0.25 calculations (b). As can be seen, smooth curves are only obtained at the MS-CASPT2 level both for the S_0 and the T_1.

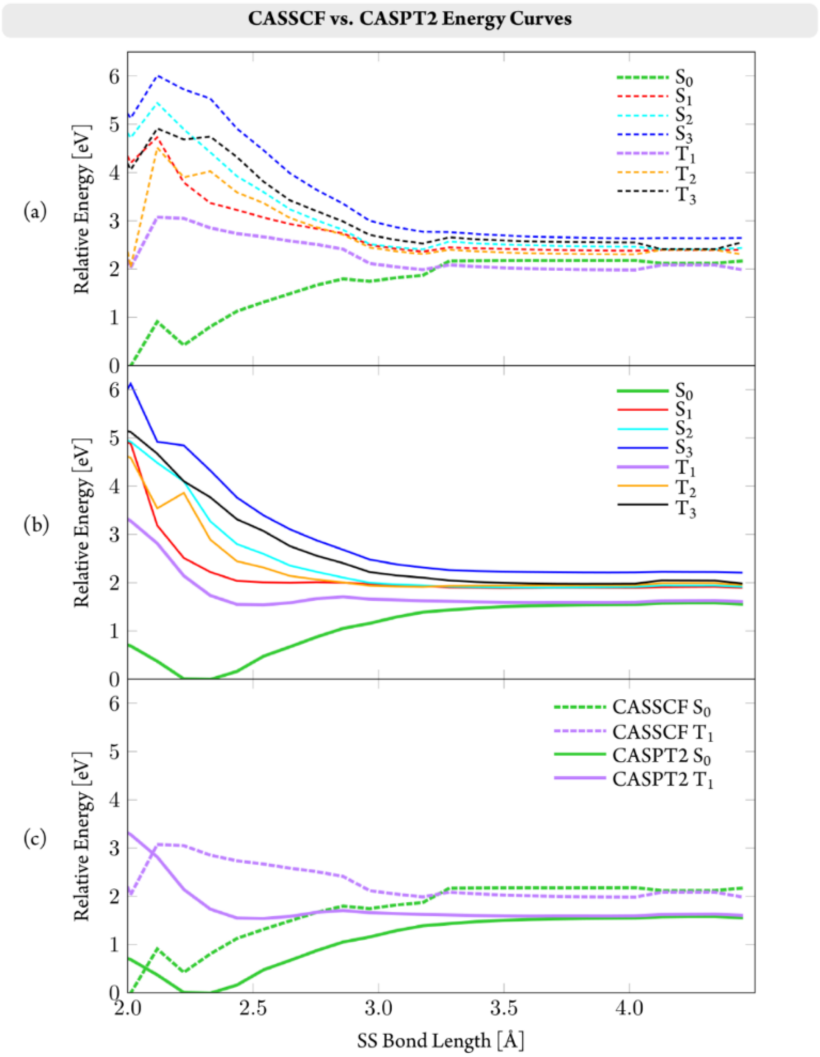

Figure S23. Comparison of CASSCF (a) and MS-CASPT(b) potential energy curves. (c) only displays the S_0 and T_1 from both calculations.

S6 Transition-Density-Matrix Analysis of Trajectories

S6.1 Pathway 2 Trajectories

In order to follow the changes in electronic character in of pathway 2 more detail, we show the transition-density analysis of an exemplary pathway 2 trajectory in Figure S24(a) alongside the changes of S−S bond length in Figure S24(b). As can be seen, until a simulation time of 60 fs, the electronic character is dominated by MLCT excitations. This is accompanied with oscillatory mixing of a number of other electronic characters, while the trajectory moves around S−S bond lengths of 2.1 Å, i.e., still close to the Franck-Condon geometry. At 60 fs, the electronic character of the exemplary trajectory changes abruptly towards the S_{loc}+L^{S}_{loc} dominated character of the pathway 2 trajectories discussed above. After the new electronic character is established, the S−S bond length starts to increase. Around 100 fs, when the bond length is close to the 2.57 Å of the T_1^{SSlong} geometry, the trajectory also adopts an electronic configuration very close to that of the T_1^{SSlong} state (Figure 8(c) in the main paper). Due to their similarity, we refer to these electronic states in the trajectories as T_1^{SSlong}-like states

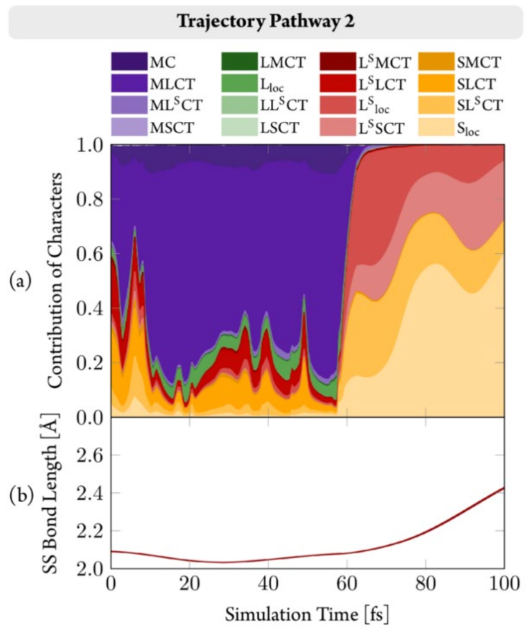

Figure S24. Analysis of a single pathway 2 trajectory. (a) Transition-density matrix analysis. (b) S−S bond length in Å.

S6.2 Pathway 1 Trajectories

In the main paper, we presented a transition-density matrix analysis of all trajectories in pathways 1 and 2 and additionally showed an example trajectory of pathway 2. Here, we also show the exemplary pathway 1 trajectories in Figure S25. Pathway 1 trajectories stay close to the Franck-Condon geometry with S−S bond lengths around 2.1 Å with electronic states of predominant MLCT character, i.e., involving charge transfer from the $Re(CO)_3$ fragment to the equatorial bpy ligand. For such trajectories we show the transition-density matrix analysis as well as the evolution of the S−S bond length in Figure S25(a,b). Some of the pathway 1 trajectories stay close to the Franck-Condon geometry but temporarily switch to other electronic states, mostly involving ligand-to-ligand charge transfer. For this, we show one exemplary trajectory of predominant $LL^{S}CT$ character in Figure S25(c,d) and one exemplary trajectory of mixed $SL^{S}CT$/SLCT/$L^{S}LCT$ character in Figure S25(e,f). All these trajectories return to MLCT-dominated electronic states after some time and do not enter S_{loc} dominated states of pathway 2 trajectories.

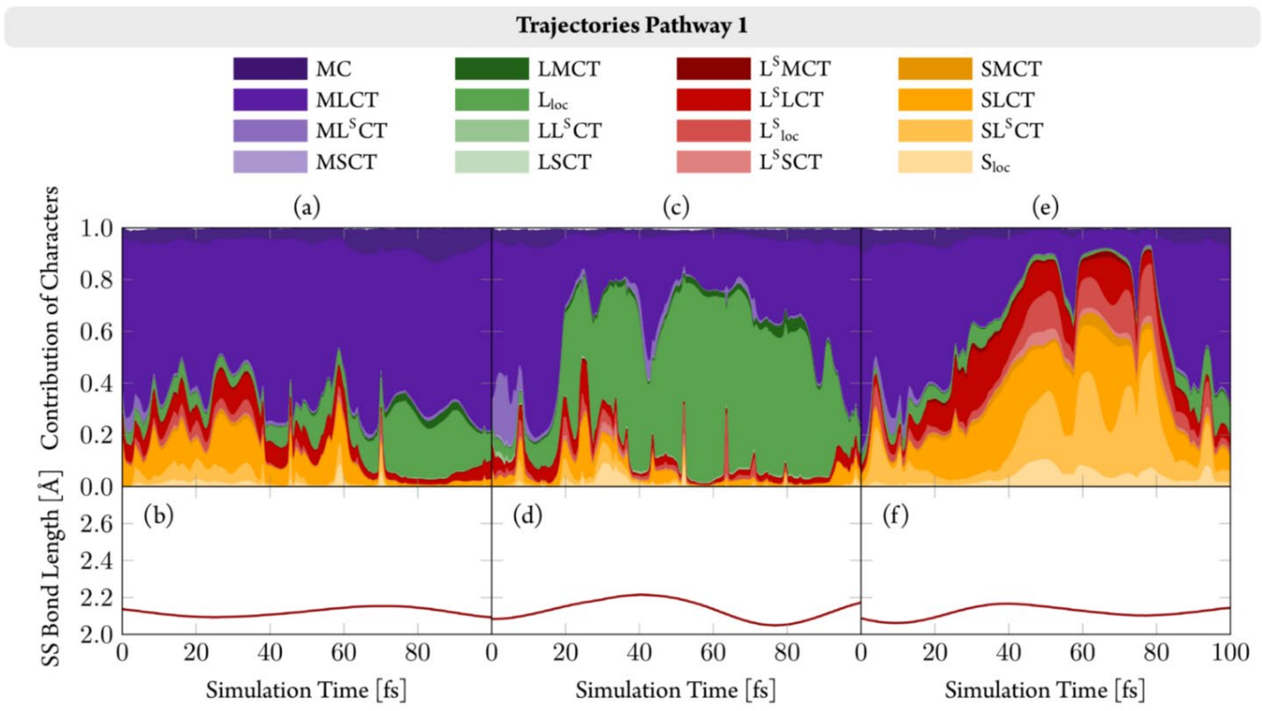

Figure S25. (a-c) Transition density matrix analysis of exemplary pathway 1 trajectories. (d-f) S−S bond length of exemplary pathway 1 trajectories.

S6.3 Electronic State Populations

The time evolution of the adiabatic electronic state populations corresponding to the trajectories assigned to pathway 1 and 2, respectively, is plotted separately in Figure S26. The T_1 state is populated faster in pathway 2 than in pathway 1. This suggests that the change in electronic character to S_{loc}-dominated contributions induces the S−S bond lengthening, which then stabilizes the respective triplet state, lowering its energy. In an adiabatic representation, this is observed as population transfer from higher lying triplets (T_n) to the T_1. From a diabatic perspective, the electronic state switches earlier (i.e. when the electronic character changes), then the populated state stabilizes, becoming the lowest lying triplet state. This stabilization happens within tens of femtoseconds, after the S_{loc} state is reached. Nonetheless, relaxation to the T_1 state still occurs on the same time scale for both pathway 1 and 2 trajectories.

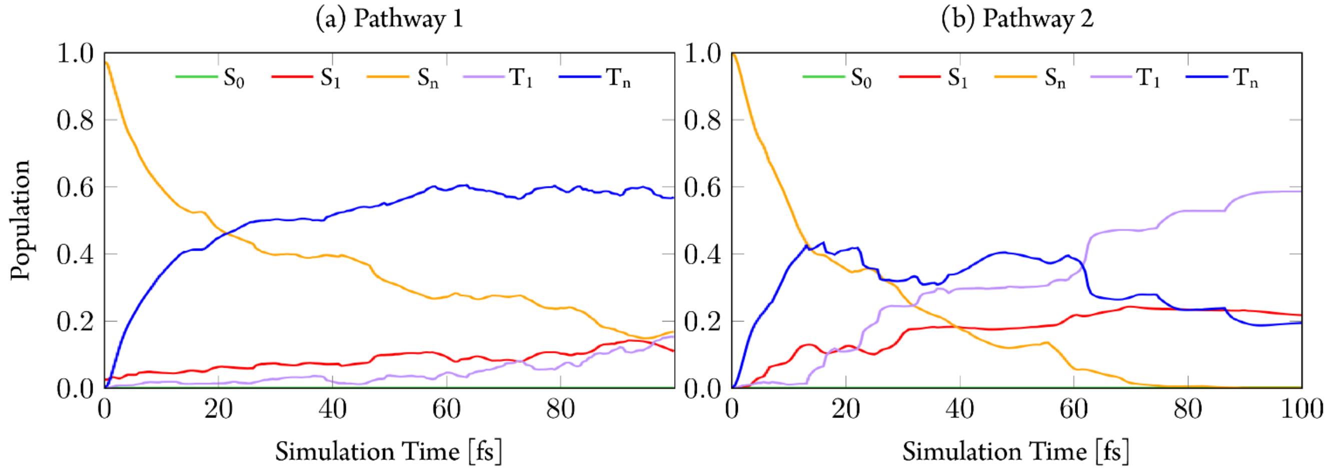

Figure S26: Adiabatic electronic state populations from TDDFT/SH dynamics for (a) pathway 1 and (b) pathway 2 trajectories.

S7 Long Time LVC Surface Hopping Dynamics

S7.1 Computational Details

S7.1.1 Parametrization of the LVC Model Hamiltonian

A linear vibronic coupling Hamiltonian[37] was constructed around the geometry of the minimum-energy conformer IV of [**1**]$^+$ for the singlet ground state as well as 15 singlet and 15 triplet excited states including all 135 vibrational degrees of freedom. Geometry optimization, frequency calculation, and excited-state calculations were performed using DFT/TDDFT with the PBE0 functional, double-zeta basis sets, and the ORCA4.2 program package as used previously (see Section S2.1).

The LVC coupling parameters were calculated using finite-differences from geometries displaced by ±0.05 a.u. along each normal mode. Intrastate coupling terms $\kappa^{(n)}$ were obtained as numerical gradients while interstate coupling parameters $\lambda^{(n,m)}$ were obtained from changes in wave function overlaps.[39] Spin-orbit couplings were taken as constant values from the reference geometry.

S7.1.2 LVC Absorption Spectrum

For a Wigner ensemble[16] of 10000 geometries from conformer 4, an absorption spectrum was calculated using the LVC model from Section S7.1.1. Figure S27 shows the obtained spectrum compared with the TDDFT based on conformer 4 and the experimental one. As can be seen, the LVC spectrum reproduces both the position of the peak as well as the pre-band shoulder of the TDDFT spectrum, although their relative intensities vary slightly. At energies higher than 4 eV, the LVC spectra falls off while the TD-DFT increases again. This is because the TDDFT calculation included 30 singlet and 30 triplet excited states. In contrast, the LVC model was restricted to 15 singlet and 15 triplet excited states, thus missing the higher-energy states that would be necessary to describe the second band. However, as these states are not likely important in the dynamics started after excitation in the first absorption band, it was not deemed necessary to include them in the LVC parametrization. Note that computing a TDDFT spectrum with only 15 singlet and 15 triplet would also not allow for a straightforward comparison between the LVC and TDDFT spectrum, as the TDDFT spectrum would include the lowest-energy states (adiabatic states) at each geometry, while the LVC spectrum always contains states of the same character (diabatic states) as is discussed e.g., in Ref. [40].

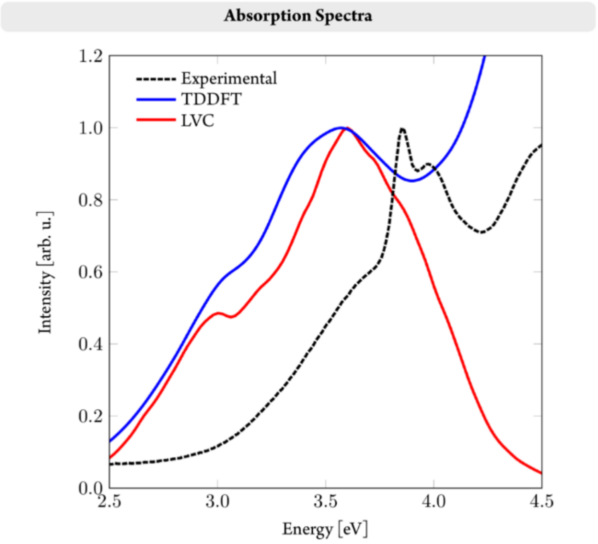

Figure S27. Comparison of experimental absorption spectrum and spectra computed using TDDFT and the LVC model. Full spectra obtained by convoluting stick spectra using Lorentzian functions of FWHM = 0.1 eV.

S7.1.3 Surface Hopping Dynamics

From the LVC absorption spectrum, 1000 initial conditions were stochastically selected[23] based on their oscillator strength within an energy window of 2.70-3.20 eV. From these initial conditions, trajectories were propagated for 10 ps with a nuclear time step of 0.5 fs and an electronic time step of 0.02 fs. Decoherence correction, surface hopping probabilities and rescaling was handled as in the short-time TDDFT/SH simulations (see Section S4.1).

S7.2 Comparison of LVC Potentials and TDDFT Potentials

The LVC potentials used in the LVC/SH dynamics are constructed in the basis of the (harmonic) normal modes of the (reference) ground-state electronic states. The coupling parameters are derived from displacements around the reference geometry. The resulting potentials –in one dimension –can take the shape shown in Figure S28. As can be seen, while the final coupled PES (green curves) are not strictly harmonic, their shape is still very much restricted to an approximate harmonic behavior when going to large displacements away from the reference equilibrium geometry.

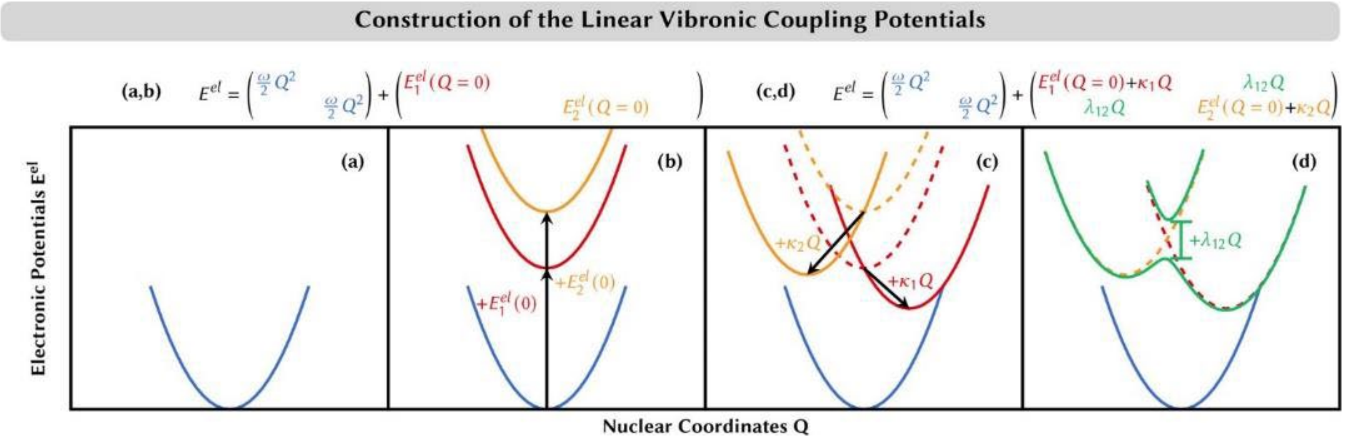

Figure S28. Construction of LVC potentials. Q_i denotes ground-state normal modes. E_i^{el} denotes vertical excitation energies. κ_i denotes intrastate couplings. λ_{ij} denotes interstate couplings. Figure taken from Ref.[41] under a creative commons license. Copyright 2021 by the Authors. Published by the American Chemical Society.

In our TDDFT/SH dynamics, we have observed pathway 2 trajectories traveling to regions of large S−S bond elongation, and we have already discussed the behavior of the TDDFT electronic potentials in comparison to multi-reference CASSCF/CASPT2 potentials in Section S.5 of this SI. Here, we are not interested in investigating how well the LVC potentials can describe the reference TDDFT potentials for this S−S bond elongation. Thus, we show in Figure S29 a comparison of LVC and TDDFT potentials of the S_0 and T_1 states from a relaxed scan in the T_1 state.

As can be seen the TDDFT potential of the ground state S_0 displays the familiar anharmonic shape of a bound electronic state. In the Figure of this relaxed scan, the T_1 appears to have a single minimum at 2.57 Å. However, as is discussed in detail more in Section S6.4 in this SI, the T_1 actually possesses a second minimum at 2.08 Å. The curves of S_0 and T_1 cross at 2.80 Å, as has also been confirmed by a minimum-energy crossing point search (see Section S5.1). The LVC potentials of both the S_0 and T_1 display a more harmonic shape. The minimum of the S_0 in the LVC model is –by construction –at the same place as the reference TDDFT S_0 minimum. The T_1 appears to have again only a single minimum based on the relaxed scan, at 2.25 Å. However, as geometry optimization in the T1 starting from different snap shots reveals, there are actually two regions of T_1 minima, as is also discussed in more detail in Section S7.4.

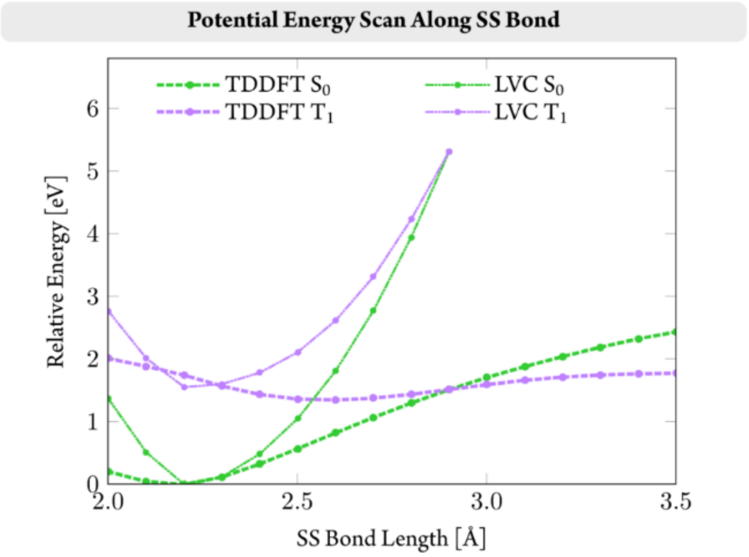

Figure S29. Comparison of S_0 and T_1 potential energy curves obtained with LVC and TDDFT from a relaxed T_1 scan along the S−S bond elongation.

For elongated S−S bond lengths, the T_1 and S_0 LVC potentials increase steeply due to their harmonic nature. Thus, the differences between the LVC and TDDFT potentials grow larger with longer S−S bond lengths. There exist also an apparent S_0/T_1 crossing point based on the shape of the relaxed scan curves at 2.8 Å, however, we were not able to identify the exact point with a minimum-energy crossing-point optimization as was possible for the TDDFT states. The most important difference between the TDDFT and LVC potential energy curves, however, lies in the differences between the T_1 minima (at longer bond lengths) and the S_0/T_1 crossing points. While the T_1^{SSlong} minimum in TDDFT is separated by the S_0/T_1 crossing point only by a small energy barrier of 0.14 eV, the differences between the T_1 minima in LVC and the approximated S_0/T_1 crossing point are ca. 3 eV. Thus, while relaxation from the T_1^{SSlong} minimum to the S_0 ground state is possible on the TDDFT potential energy surface, this pathway is closed by the artificially high energy barrier in LVC, thus confining trajectories to remain in region B in the potential energy surface.

S7.3 Comparison of LVC/SH and TDDFT/SH Dynamics

In Figure S30 we show a comparison of the electronic state populations from the LVC (a) and TDDFT (c) surface hopping dynamics for the first 100 fs simulation time including time constants based on the mechanism discussed in the paper. For the LVC/SH dynamics up until 100 fs, no reaction to the S_0 ground state was observed. Accordingly, this reaction was excluded from the present fits.

As can be seen, the time evolution of the population proceeds very similar in both simulations. The main difference is that internal conversion within the triplet manifold in the LVC/SH dynamics occurs more rapidly, leading to a faster population of the T_1.

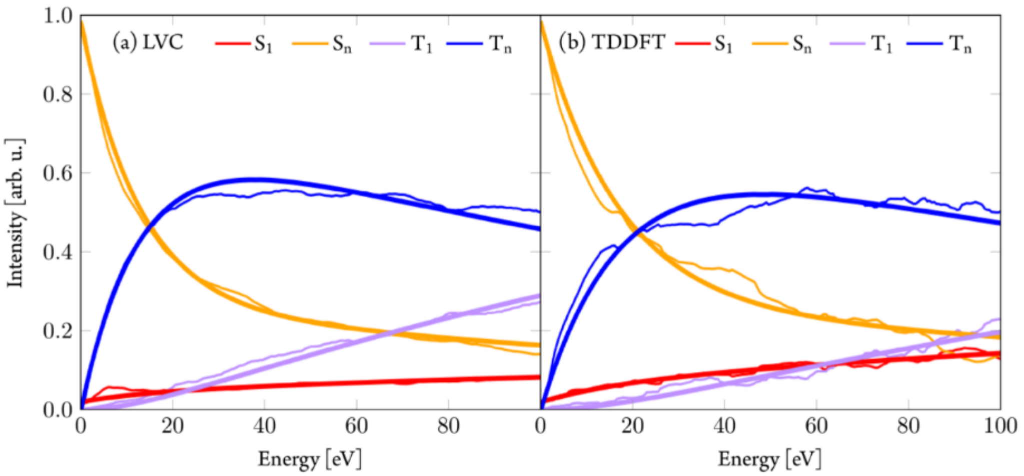

(c) Time constant [fs]	LVC/SH	TDDFT/SH
τ_{ISC}	18 ± 0.6	25 ± 3
τ_{bISC}	53 ± 3	69 ± 17
τ_{T}	171 ± 11	249 ± 50
τ_{S}	469 ± 38	285 ± 72

Figure S30. Electronic state populations from surface hopping simulations using (a) LVC and (b) TDDFT potentials. (c) Time constants from fits including error estimates from 100 bootstrap copies. Fit only performed for data until 100 fs.

We note that as in the TDDFT/SH simulations, we include a back-intersystem crossing reaction from the T_n to the S_n states in our kinetic model. However, this inclusion is a consequence of us using a simplified representation of "spin-free" states, i.e., pure singlet and triplet states that only exist in the absence of spin-orbit coupling. In the dynamics, most trajectories are in mixed-spin states in the beginning of the dynamics. Only later, upon reaching the lower-energy states, the spin assignment of these trajectories becomes clearer. This can be seen in Figure 12 in the main paper, which plots the energy gap to the ground state as a function of the S−S bond length for adiabatic states distinguished by their spin expectation value. As can be seen, at the beginning of the simulation (0 fs), most trajectories are in a spin-mixed state. However, with ongoing simulation time, this character is quenched and almost all trajectories end up in either "pure" singlet or triplet adiabatic states with spin-expectation values close to 0 (singlets) or 2 (triplets)

S7.4 Triplet Optimization from Snapshots of the LVC/SH Dynamics

Furthermore, we have performed geometry optimizations of the T_1 state from various snapshots of the LVC/SH dynamics. Geometry optimizations were first performed using the LVC potentials. These calculations were performed using ORCA4.2 while employing SHARC as an external program to provide the LVC potentials. Starting from the LVC-optimized structures, geometry optimization for the T_1 were performed using the TDDFT setup also used described in Section S4.1.

Optimizations were performed for two batches of snapshots. First, we took 100 snapshots of geometries in region B in the dynamics (see Figure 12 in the main paper) and optimized them in the LVC model. A superposition of the optimized structures is shown in Figure S31(a) (thin lines) where we have included the T_1^{SSlong} TDDFT-optimized geometry as a reference (thick lines). The LVC-optimized T_1 structures

display S−S bond lengths between 2.25 and 2.35 Å. Their S−S bond length is considerably increased in comparison with the ground-state equilibrium structure (2.06 Å), while still being smaller than that of the T_1^{SSlong} TDDFT-optimized geometry (2.57 Å). We note also that all these geometries are found as minima in the LVC potential energy surface, showing a somewhat greater flexibility as is usual found for molecular potential energy surfaces, that typically possess less, but more distinct minima. From the LVC-optimized T_1 minima, we took 30 samples and subsequently optimized them at the TDDFT level of theory. All of them converged to the T_1^{SSlong} minimum, showing that these LVC-T_1 minima indeed corresponded to the T_1^{SSlong} TDDFT minimum. This also supports our assumption that region B trajectories in the LVC/SH dynamics correspond to the pathway 2 trajectories from the TDDFT/SH dynamics.

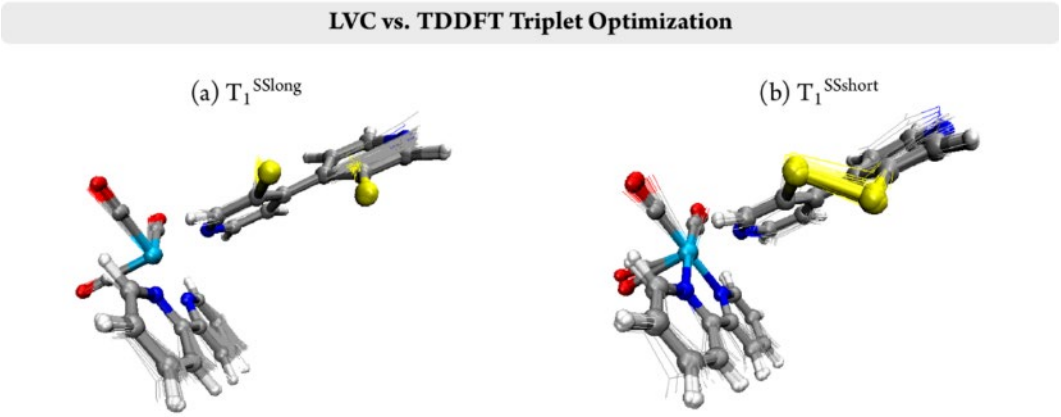

Figure S31. Superposition of LVC and TDDFT T_1 optimized structures. Thin lines are LVC-optimized structures, while thick lines are TDFT optimized structures. (a) LVC geometries with long S−S bond lengths on top of T_1^{SSlong}. (b) LVC geometries with short S−S bond lengths on top of $T_1^{SSshort}$.

Second, optimizations were performed for snapshots from the LVC/SH dynamics where trajectories were in region A. For this, we selected 100 geometries at 300 fs with SS bond lengths between 2.05 and 2.15 Å where the active state was the T_1. Among these, LVC geometry optimization of the T_1 reached two different regions: one with an energy gap to the ground state ΔE of ca. 1.22 eV and an S−S bond length around 2.25 Å, which is very similar to the T_1-LVC-optimized structures discussed above. This region was reached for 30 snapshots. The remaining 70 snapshots converged to a different region with S−S bond lengths closer to the equilibrium value (ca. 2.08 Å) and energy gaps ΔE around 1.83 eV. These optimized structures are shown in Figure S31(b) as thin lines. From these, we took 30 samples and optimized them at the TDDFT level of theory. All converged to the same structure, i.e., the $T_1^{SSshort}$ also shown in Figure S31(b) as thick lines. The $T_1^{SSshort}$ minimum is characterized by an S−S bond length of 2.08 Å an energy gap ΔE of 1.99 eV, i.e., very similar to the experimentally observed phosphorescence energy (2.16 eV).

Vibrational frequencies of the C-O bonds in the CO-ligands were computed for the optimized ground state structure, as well as the two triplet minima. The calculated vibrational frequencies for Figure 4c of the main text were multiplied by a 0.955 correction factor,[38] to accommodate for known errors of DFT computed vibrational frequencies of organometallic compounds.

S7.5 Connection Between the Two Triplet Minima

Adiabatic excitation energies of the S_0, S_1, and T_1 states at the optimized S_0, T_1^{SSlong}, and T_1^{SShort} geometries are given in Table S3. A superposition between the S_0 and $T_1^{SSshort}$ structures as well as the S_0 and T_1^{SSlong} structures is shown in Figure S32. As can be seen, the geometries of the S_0 and $T_1^{SSshort}$ minima are very similar showcasing that $T_1^{SSshort}$ minimum is close to the Franck-Condon region, whereas the S_0 and T_1^{SSlong} structures differ mainly in the S−S bond lengths.

Table S3. Adiabatic excitation energies of the S_0, S_1, and T_1 states at the optimized S_0, T_1^{SSlong}, and T_1^{SShort} geometries.

Energy [eV]	S_0-Geometry	T_1^{SSlong} -Geometry	$T_1^{SSshort}$ -Geometry
State S_0	0.00	1.00	0.26
State S_1	2.75	2.16	2.43
State T_1	2.42	1.56	2.25

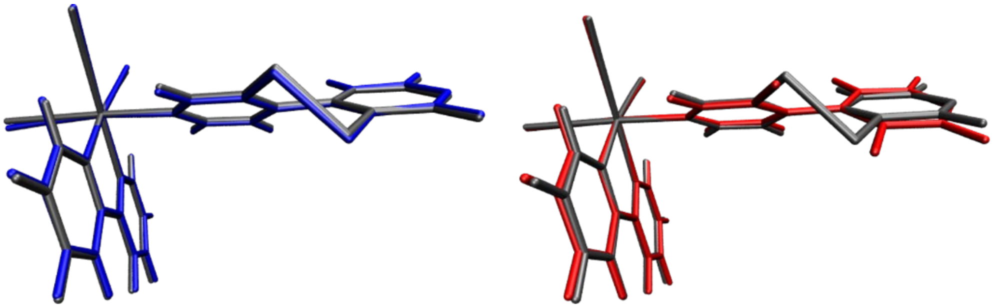

Figure S32. Superposition of S_0 (gray) and $T_1^{SSshort}$ (blue) and of S_0 (gray) and T_1^{SSlong} (red) structures.

An attempt to locate a transition state between the T_1^{SSlong} and T_1^{SShort} minima using the nudged-elastic band method was unsuccessful. Instead, we performed a linear interpolation between the $T_1^{SSshort}$ and T_1^{SSlong} minima as well as a relaxed scan along the S−S bond length –the most characteristic difference the two structures –to explore important features of the T_1 potential energy surface that connect both minima.

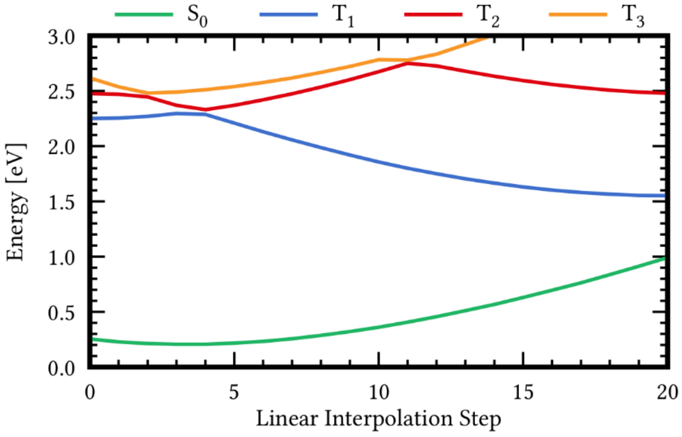

Figure S33. Potential energy curves of the S_0, T_1, T_2, and T_3 states along a linear interpolation path between the $T_1^{SSshort}$ (step 0) and T_1^{SSlong} minima (step 20).

The linear interpolation between the $T_1^{SSshort}$ (step 0) and T_1^{SSlong} minima (step 20) is shown in Figure S33. At the $T_1^{SSshort}$ geometry, the T_1 state (blue curve) corresponds to the predominantly ^{3}MLCT state as shown in Figure 13(c) in the main paper, whereas the T_3 state features a dominant ^{3}LC contribution similar to the T_1 state at the T_1^{SSlong} geometry. At the $T_1^{SSshort}$ geometry, the T_2 state is another state of ^{3}MLCT character. At step 2, there is a crossing between the T_2 and T_3 states signaling a switching in the state

character of these two adiabatic states, and at steps 4-5, there is crossing between the T_1 and T_2 states, indicating the point where the ^{3}LC-type state becomes the lowest triplet state. Compared to the T_1 energy at the $T_1^{SSshort}$ geometry, the T_1 energy at steps 4-5 shows a barrier of ca. 0.1 eV. Since this barrier is obtained from a linear interpolation and not a relaxed scan between the two minima, it should be interpreted as an upper bound to any barrier on the real (TDDFT-calculated) potential energy curves. However, since it is close to the starting point of the scan, it is likely a good approximation.

Figures S34 show relaxed scans along the S−S bond between 2.0 and 2.8 Å in the S_0 state (a) and in the T_1 state (b). Calculations were performed in steps of 0.025 Å in the range of 2.0 and 2.2 Å and in larger steps of 0.075 Å in the range of 2.2 and 2.8 Å. In both scans, the T_1 adopts a predominantly ^{3}LC character (local excitation at the $^{S-S}$bpy^{4,4} ligand) at each relaxed geometry, indicating the large influence of S−S bond has on the energy of the ^{3}LC state. At the 2.06 Å reference value of the S_0 minimum geometry in the S_0-relaxed scan, i.e., at a point close to the Franck-Condon region, the T_1 state at an excitation energy of ca. 2.4 eV and it is stabilized upon increasing the S−S bond length. In the T_1-relaxed scan, the potential energy curve of the T_1 state is very flat in the region around S−S bond lengths of 2.1 Å. This is despite the T_1 already being of ^{3}LC character, a state described by an excitation from the sulfur p orbitals to the σ* orbitals of the S−S bond that is in general stabilized by elongation of the S−S bond length. This shape of the T_1 potential in principle might enable a molecule in the ^{3}LC state to dwell for short times in this region of S−S bond distances with possibility to return to other triplet states, e.g., states of ^{3}MLCT character. However, it seems more likely that the fate of the molecule to move towards larger S−S bond is decided once entering the ^{3}LC -type state.

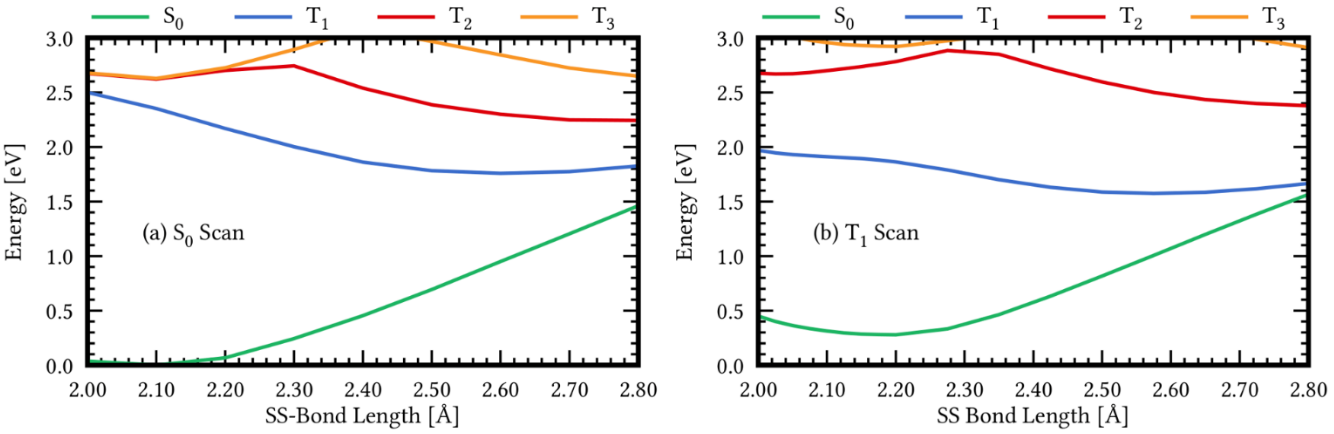

Figure S34. Relaxed scan along the S−S bond (a) in the S_0 state and (b) in the T_1 state.

While the linear-interpolation scan between the $T_1^{SSshort}$ and T_1^{SSlong} minima showed a small barrier the regions of ^{3}MLCT and ^{3}LC characters, a relaxed scans along the S−S bond distance suggest that once a triplet state assumes ^{3}LC character, it relaxes towards the T_1^{SSlong} minimum.

S7.6 Electronic state populations

To further investigate the differences between the trajectories that populate region A (group A) and region B (group B, see Figure 12 in the main paper), the time evolution of the adiabatic electronic states were plotted for trajectories from both regions separately in Figure S35. It can be seen that both groups exhibit rapid ISC from the initially excited singlet states to the triplet manifold. Furthermore, both groups populate the lowest lying triplet state T_1 within a couple hundred of femtoseconds. However, trajectories from group B seem to experience a slightly faster population of the T_1. This can again be explained by the fast stabilization of the S_{loc} state due to S−S bond lengthening, as already mentioned for the TDDFT/SH dynamics in Section S6.3. Additionally, group B trajectories shows back-transfer from the T_1 to higher lying

triplets, whereas group A trajectories almost exclusively populate the T_1 after 1 ps of simulation time. This behavior is due to the significant energy differences between region A and B (as seen in Figure 12). The triplet states of the group A trajectories are close in energy, making it feasible to hop back into a higher lying state during the simulation. By contrast, once a trajectory reaches region B, the energy of the T_1 is significantly lowered, separating it from the higher-lying triplet states, thus making a hop back to T_n states unlikely.

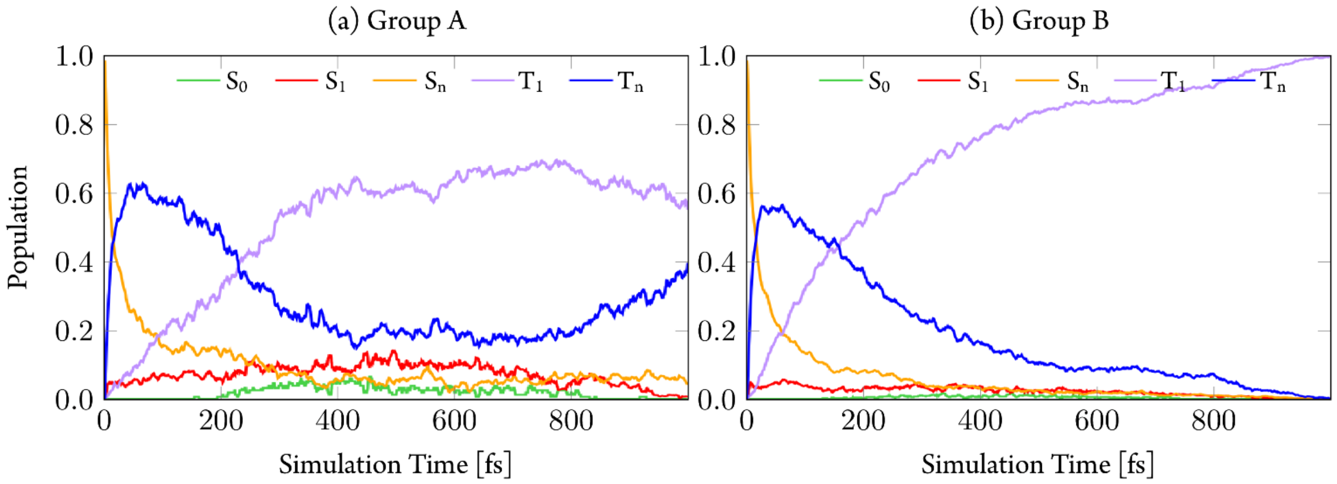

Figure S35. Adiabatic electronic state populations of the LVC/SH dynamics for trajectories from (a) Group A and (b) Group B, respectively.

S8 Excited-State Singlet Minima

In order to further characterize the excited-state potential energy landscape, geometry optimizations of the lowest-lying singlet excited state S_1 were conducted. These calculations were performed at the same level of theory as for the triplet optimizations. Two optimizations were started at the geometries of the T_1^{SSlong} and $T_1^{SSshort}$ states. Both optimizations resulted in distinct S_1 minima that resemble their triplet starting points. The states are labeled S_1^{SSlong} and $S_1^{SSshort}$ and are shown in Figure S36 alongside the natural transition orbitals and transition-density matrix analysis to characterize them. The S_1^{SSlong} minimum lies at an excitation energy of 1.06 eV with an S−S bond length of 2.57 Å. Its character is given by excitations within the $^{S\text{-}S}$bpy^{4,4} ligand with a mixture of S_{loc}, L^SSCT, SL^{S}CT and $L^S{}_{loc}$ excitations. The character is very similar to that of the T_1^{SSlong} with slightly more L^SSCT character in place of S_{loc} character (compare to Figure 8). The $S_1^{SSshort}$ minimum lies at an excitation energy of 2.16 eV with an S−S bond length of 2.08 Å. Its character is given by mainly by MLCT excitations (81%) with the second-largest contribution from L_{loc} excitations of only 6%. This character is similar to the that of the $T_1^{SSshort}$ state (Figure 13), however, the differences between the $S_1^{SSshort}$ and $T_1^{SSshort}$ characters appear somewhat larger than between the S_1^{SSlong} and T_1^{SSlong} states. The coordinates of both optimized geometries are also reported in Section S11.

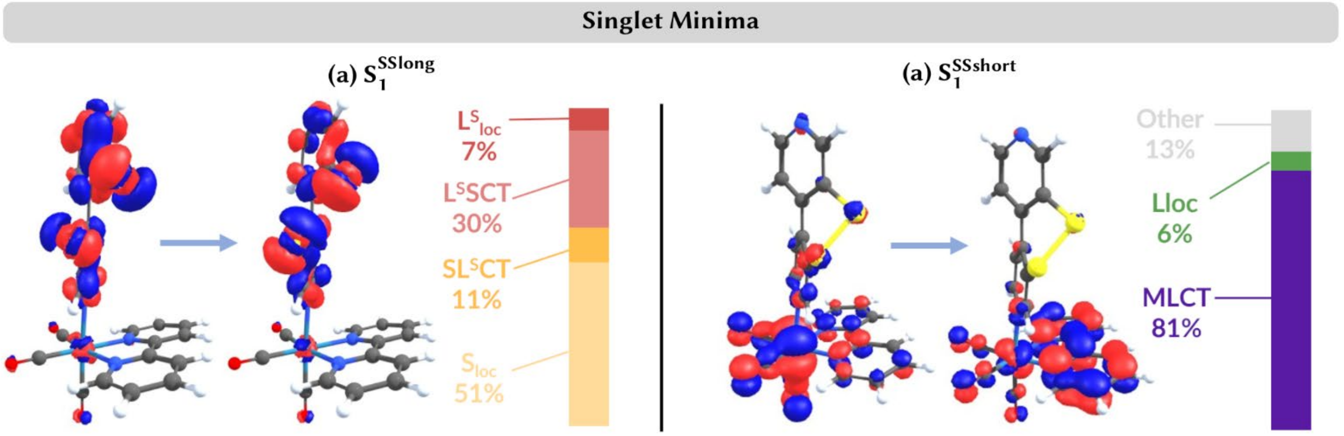

Figure S36: Natural transition orbitals of and leading contributions of the transition-density matrix analysis of the two singlet minima S_1^{SSlong} and $S_1^{SSshort}$.

S9 Charge Redistribution in the Triplet Minima

In the manuscript, we show a transition-density-matrix analysis including natural transition orbitals in Figures 8 and 13 to characterize the electronic character of the T_1^{SSlong}, and $T_1^{SSshort}$ states at their optimized geometries, respectively. This analysis gives a direct picture of the charge differences of the excited state compared to the ground state at the geometries. Unfortunately, it is not straightforward to compare directly the charge redistribution from the S_0 state at its optimized geometry to that of the triplet state at their optimized geometries in the same way, as the orbitals and electron densities available from the calculations are obtained at different geometries and thus do not overlap. We can, however, compare the charge distributions between the different geometries in an approximate manner by using atomic properties. For this, we have calculated the Lowdin charges of the three states S_0, T_1^{SSlong}, and $T_1^{Ssshort}$ at their optimized geometries as shown in Figure S37(a-c). The Lowdin charges are represent as colored circles whereby red/blue denote negative/positive Lowdin charges of a size corresponding to the radius of the circle. As can be seen, the largest charges are present on the Re atom as well as on the carbon atoms of the CO ligands. Further notable charges are found on the nitrogen atoms of both bipyridyl ligands as well as on the sulfur atoms of the S−S unit.

As differences between these charge distributions are difficult to spot in this representation, we have also calculated the differences between the Lowdin charges at the T_1^{SSlong}/$T_1^{Ssshort}$ at their optimized geometries and the S_0 state at its optimized geometry in Figure S37(d/e). Here, the red/blue circles denote larger/smaller charges in the triplet state, and the radius of the circles corresponds to these differences scaled by a factor of 5 –compared to absolute values of Figure 37(a-c) –for better visibility.

As can be seen in Figure S37(d), the charge difference in comparing T_1^{SSlong} and the S_0 state is almost entirely given by contributions from the $^{S\text{-}S}$bpy^{4,4} ligand. As can be seen, the charge flow is directed towards the lower sulfur atom located closer to the Re metal center, i.e., the sulfur atom bearing the red circle. Most of the charge flow originates from the other sulfur atom (bearing a blue circle), with additional smaller contributions mostly from the carbon atoms in the upper pyridyl unit, i.e., the unit farther away from the Re center. For the difference between the $T_1^{Ssshort}$ and S_0 states at their optimized geometries. Figure S37(e) shows a large shows a charge flow from the Re metal center and –to a lesser extent –the CO ligands towards the 2,2’-bpy ligand. Among the three CO ligands, a larger charge donation is given by axial CO ligand than by either of the two equatorial CO ligands, as denoted by the larger size of the circle.

Summarizing, the atomic charge differences yield a similar description as the transition-density-matrix analyses shown in Figures 8 and 13, despite the former also including the effects of the distinct geometries. This is likely because the excitation T_1^{SSlong} state is localized on the $^{S\text{-}S}bpy^{4,4}$ whereas the geometry of the $T_1^{SSshort}$ state is very similar to that of the S_0 ground state. Thus, the transition-density matrix analyses shown in Figures 8 and 13 capture the essential properties of the excited triplet states.

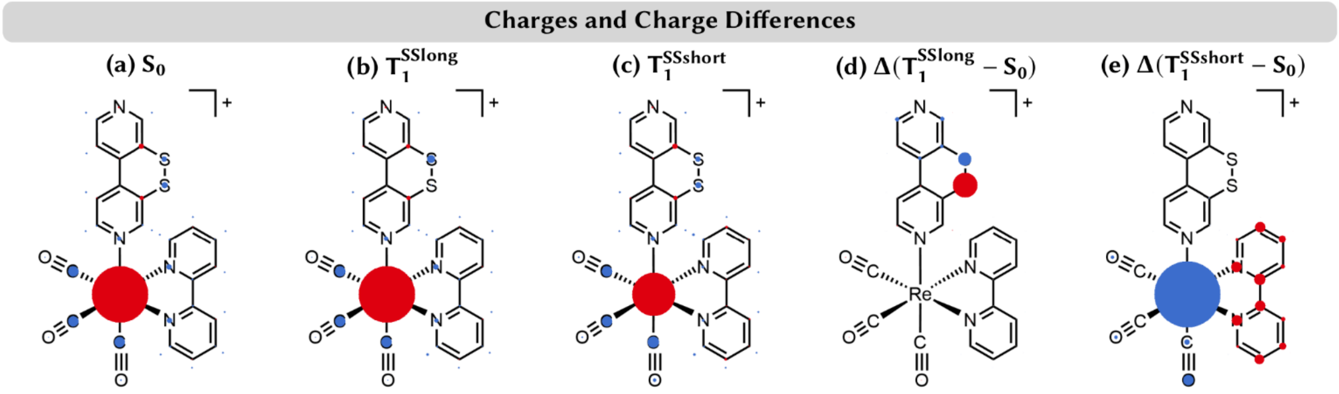

Figure S37. (a/b/c) Lowdin charges computed for the S_0 state, T_1^{SSlong}, and $T_1^{SSshort}$ states at their optimized geometries. Red/blue circles denote negative/positive charge population. The radius of the circles corresponds to the size of the charge. (d/e) Difference between the Lowdin charges at the $T_1^{SSlong}/T_1^{SSshort}$ at their optimized geometries and the S_0 state at its optimized geometry. Red/blue circles denote larger/smaller charges in the triplet states. The radius of the circles corresponds to the difference of the charges scaled by a factor of 5. Hydrogen atoms are omitted in the structure drawing, but Lowdin charges and charge differences of the hydrogen atoms are shown (wherever applicable) in this Figure.

Furthermore, the cause of the blue-shift in the C-O vibrations of the CO-ligands seen in both triplet minima was investigated. For $T_1^{SSshort}$, the C-O bands show a typical blue shift associated with the ^{3}MLCT character. In T_1^{SSlong} the charge transfer is localized at the axial $^{S\text{-}S}bpy^{4,4}$ ligand. Nonetheless, a similar blue shift of the C-O stretching frequencies is seen in the computed IR spectrum. The Mulliken charges of Re and the three CO-ligands are shown in Table S4. Both triplet minima show a decrease in electron density at the CO-ligands compared to the ground state minimum, offering an explanation about the similar blue shift of the C-O bands.

Table S4: Mulliken charges of Re and the three CO-ligands in the ground state minimum (S_0), the two triplet minima and their respective differences wrt the S_0.

Atom	S_0	$T_1^{SSshort}$	Δ ($T_1^{SSshort}$ - S_0)	T_1^{SSlong}	Δ (T_1^{SSlong} - S_0)
**Re**	0.4469	-0.4412	-0.8881	-0.4292	-0.8761
**C_1**	-0.0251	0.2162	0.2413	0.2349	0.2600
**O_1**	-0.1568	-0.0463	0.1105	-0.0847	0.0721
**C_2**	-0.0220	0.2138	0.2358	0.2243	0.2463
**O_2**	-0.1576	-0.0471	0.1105	-0.0850	0.0726
**C_3**	0.0051	0.2563	0.2512	0.2644	0.2593
**O_3**	-0.1610	-0.0298	0.1312	-0.0768	0.0842

S10 Parameters

The parameters of the LVC model employed in the present LVC/SH dynamics is provided as an additional supporting information zip file.

S11 Geometries of Optimized Structures

Here we report the coordinates of the optimized structures of the Franck-Condon geometry (conformer 4) and the two triplet minima T_1^{SSlong} and $T_1^{SSshort}$. All coordinates are given in Å.

Franck Condon Geometry (Conformer 4)

Re	0.06983202910478	-0.07094228246069	-0.02272358229834
S	5.82720439034522	-0.40416567162973	0.31180354420503
O	1.18495505473596	2.77242397773349	-0.19865066958256
N	1.99853151472087	-0.95664257468177	-0.62382112200072
C	3.14253610804802	-0.54835106906773	-0.07137646857160
H	3.07778525879183	0.21317040983460	0.69510745416105
O	-0.84426745685694	0.24024016946070	-2.92637362587400
S	6.80055737396650	-2.19266061916191	0.56780183044911
N	8.13499413734495	-3.61454563425381	-2.91056505448910
C	4.37584719006070	-1.04910951198860	-0.44153566023822
O	-2.61362135043101	1.07953926451849	0.88867209198898
N	0.72910729563144	-0.54108555955267	1.98645009886375
C	4.45447938542634	-2.02011813933902	-1.44849355888024
N	-0.54525548529916	-2.11976296175184	0.30810463922911
C	3.24917088080369	-2.45964883902271	-1.98288949116711
H	3.22484731685723	-3.23452037459484	-2.73715160847931
C	2.05990374142013	-1.91148855549792	-1.55815725468456
H	1.12337314978811	-2.24627046253804	-1.98344840573112
C	5.73310535305231	-2.54615722598233	-1.94250282441794
C	6.86535665940533	-2.66005245558888	-1.12743050191210
C	8.03033004632990	-3.20233421916527	-1.65453531847498
H	8.91075169810588	-3.30190667881485	-1.02658996797542
C	7.07244572020002	-3.47723876751095	-3.69737945235856
H	7.18568844630924	-3.79595363906148	-4.72848314337562
C	5.86642108889484	-2.95323760463548	-3.26501030649238
H	5.04502909930253	-2.85186466189945	-3.96290632770990
C	1.33328590871860	0.32982602313749	2.80007710747690
H	1.43864946834584	1.34273796061111	2.43477283829627
C	1.80531611186162	-0.02295163324558	4.04719832517930
H	2.29040241867676	0.72146561551662	4.66427628222453
C	1.00077716318649	-2.22963691487368	3.64316398554704
H	0.85486454852173	-3.25103677841031	3.96362567684693
C	1.64201461354992	-1.32967910542786	4.47293242521423
H	2.00448431302162	-1.64666055905223	5.44287649899625
C	0.54674689866477	-1.80953496550924	2.40287000149284
C	-0.50852763006572	-3.99557182891876	1.77389076868009

H	-0.20953639186581	-4.43704888669606	2.71359753767731
C	-1.23575930532920	-4.73663209264505	0.86187905778594
H	-1.50348332228052	-5.76202046293909	1.08410409757347
C	-1.61944244032010	-4.14649860106587	-0.32963869832654
H	-2.19648101852829	-4.68181991812579	-1.07164386328383
C	-1.25412452988782	-2.83780758617865	-0.56707224516699
H	-1.54031938838961	-2.33961958874851	-1.48373517882525
C	0.76257693245405	1.70594073366691	-0.13697516104426
C	-0.50014677581578	0.12358954791378	-1.83667384216593
C	-1.60739608857985	0.64520376866627	0.55012204662903
C	-0.17836505778570	-2.68323014425738	1.47599846418682

T_1^{SSlong}

Re	0.01820960906746	-0.04966180027400	-0.03435110045572
S	5.66600367788011	-0.39402569512582	0.62552233266452
O	1.09197112384870	2.82400314217338	-0.16407854029827
N	1.97130104020647	-0.89838474874585	-0.63401318619893
C	3.09038516362100	-0.53359972217972	-0.01818800609387
H	3.00393842281066	0.19774960130154	0.78216360440714
O	-0.93438465659925	0.11580324237972	-2.94749644262762
S	7.02786291334141	-2.58183252080083	0.60056569483601
N	8.01896155882182	-3.81338015716482	-2.98886378443843
C	4.37344273464642	-1.02417479480993	-0.33038365784536
O	-2.71280136661960	1.01232988845631	0.89111311880337
N	0.68612031885076	-0.48201446161056	1.98660082908901
C	4.48126036288227	-1.96227836606637	-1.38857930238156
N	-0.51809568761663	-2.12568364107325	0.30562973171354
C	3.27811630144558	-2.34171957683236	-2.01093279154466
H	3.28714658206452	-3.08853961761841	-2.80106364102925
C	2.06694670177769	-1.80993553075839	-1.62323039883134
H	1.14388923089163	-2.10994149006257	-2.11184260928849
C	5.73882896779701	-2.53179349166536	-1.89052112661951
C	6.84874562568754	-2.89652145430538	-1.08069064925278
C	7.94318613537445	-3.55871444682995	-1.70235832529866
H	8.79017670948990	-3.85586819503897	-1.07991555130670
C	6.99527709240922	-3.43270007739339	-3.75858686392253
H	7.08197229268556	-3.64202188154204	-4.82583894023120
C	5.85838090696522	-2.80928031604312	-3.26054366731688
H	5.07004061375755	-2.51482963566799	-3.94948782945940
C	1.23126111487684	0.42925401073882	2.80453417024042
H	1.27308707030175	1.45133658957115	2.43434335576751
C	1.71929707038524	0.10646475021771	4.06293925066242
H	2.15180722262300	0.88312405625644	4.68892750394183
C	1.06012701886047	-2.15912076400785	3.64807356778458
H	0.97773767697466	-3.19312162913956	3.96950776906579
C	1.63933585702564	-1.21651562217345	4.48907188969682
H	2.01490878392711	-1.51045628708653	5.46716152319714
C	0.58231715783273	-1.76501461861098	2.39778086799739
C	-0.33475554394381	-4.02567464681565	1.74385668352774

H	0.00956032320262	-4.46464103400145	2.67543962681408
C	-1.03196317319476	-4.80379074333802	0.82758526318574
H	-1.22995636281270	-5.85322424963266	1.03667577108887
C	-1.48063866341660	-4.21487112626018	-0.35145588687108
H	-2.04351467872591	-4.77720624116368	-1.09273618725000
C	-1.19985244170105	-2.87378374224899	-0.57230986268965
H	-1.53753993108906	-2.37318706450990	-1.47713088748001
C	0.67533799249007	1.75297842712290	-0.14077713207539
C	-0.57181022213143	0.08174695019189	-1.85628720361304
C	-1.69236402903976	0.61927030322039	0.54474341025256
C	-0.09461954372071	-2.68022067426696	1.46298904886120

$T_1^{SSshort}$

Re	0.02915420981106	-0.07078972017717	-0.02022355774710
S	5.77770484236333	-0.45393333626004	0.38905784261340
O	1.11895549117843	2.81614366835854	-0.31136301831785
N	1.94791251292171	-0.97183118331707	-0.61382417097103
C	3.09094396430983	-0.57756918751920	-0.03855419917152
H	3.02596280209020	0.17047067341699	0.74841680769084
O	-0.83631089555346	0.32317791194252	-2.97128615595921
S	6.75269616882864	-2.27869141976286	0.62581071653897
N	8.12770249751954	-3.60033777996618	-2.89257265264877
C	4.33309099850061	-1.09047155881473	-0.39748723297749
O	-2.75516105370620	0.87335683184619	0.98228496543639
N	0.71507239892807	-0.47236167628468	1.93463351007110
C	4.41435105549366	-2.05910200970241	-1.42128244775539
N	-0.54075432407095	-2.08968159687786	0.24332724444926
C	3.19977246909875	-2.49127706689331	-1.96488686090843
H	3.17920092061193	-3.27424807432098	-2.71938994521256
C	2.00346565812881	-1.93141467872585	-1.55019316850263
H	1.06088983493593	-2.26471643502094	-1.97651674068315
C	5.70060299993256	-2.57510302962961	-1.91942063570239
C	6.83329353279925	-2.69640904357450	-1.09134604757332
C	8.01457580567302	-3.21780728314768	-1.62828202136340
H	8.89759721225218	-3.31725263413567	-0.99332497741943
C	7.07505713209933	-3.46123723505196	-3.69064539866573
H	7.20572652978777	-3.75730325483945	-4.73278346979333
C	5.85073332685257	-2.95514313059623	-3.25726973745459
H	5.03489472258229	-2.83627155680321	-3.96780056238401
C	1.29302624185442	0.41841525045670	2.77240460062258
H	1.34769615864518	1.44581799622695	2.41766958329395
C	1.78875855992721	0.07719373300348	4.00545443533652
H	2.24354951911350	0.83404688691629	4.63770389741506
C	1.08395127384662	-2.19188554811122	3.59459181987082
H	0.98958911538955	-3.22691189806824	3.90947117727590
C	1.68521227434111	-1.27619823346488	4.42024980289987
H	2.07205821274858	-1.58352137908326	5.38928919322796
C	0.57532911175875	-1.79540390728145	2.33330165280111
C	-0.36534326869604	-4.02951351842157	1.67551774020626

H	-0.01515474968183	-4.48653039019463	2.59625503616011
C	-1.07213970831009	-4.77435219293079	0.76588118427791
H	-1.27894853198709	-5.82441747553749	0.96046144416169
C	-1.54266089641844	-4.16128424490047	-0.42409247587341
H	-2.12136603827527	-4.71272623564234	-1.15946148956747
C	-1.25377098594950	-2.83505577037661	-0.62965080902515
H	-1.60032448236647	-2.32372686942165	-1.52578698618401
C	0.71153762931204	1.75332380417686	-0.20014724621077
C	-0.52287843582762	0.17408713436226	-1.88140671400820
C	-1.73140726848349	0.54421985901625	0.61042622777620
C	-0.10029979830999	-2.65966258286679	1.42976533095443

S_1^{SSlong}

Re	0.01175198955870	-0.04602153110743	-0.02745072476212
S	5.67115117490545	-0.35518697976912	0.61460423397251
O	1.04894401149930	2.84189778746754	-0.14360509049020
N	1.97746623567093	-0.86908381621087	-0.62393061155873
C	3.09144891840307	-0.52233069867544	0.00762901660608
H	3.00229202479993	0.18368532052111	0.83048681879927
O	-0.93984222864821	0.12043467161839	-2.94113578684025
S	7.12150179934620	-2.48120270895261	0.55979685288951
N	7.93889991511347	-3.94708394999668	-2.96271165970308
C	4.38228668685189	-0.99267408293914	-0.32106321876351
O	-2.73629962452869	0.97848720810154	0.88988716371547
N	0.67405987727996	-0.47805783186473	1.99468923975844
C	4.49557917457563	-1.90974978923541	-1.40115626475792
N	-0.49914261292045	-2.13073348328615	0.30097780005871
C	3.29185434920632	-2.27175596001302	-2.03690360157468
H	3.30035851782192	-3.00100384933461	-2.84301717563640
C	2.07990805344192	-1.75145028802572	-1.64206890337902
H	1.15998998556222	-2.03744285170243	-2.14437149095058
C	5.74635422138275	-2.48895881554624	-1.89850727372801
C	6.85038597492738	-2.87553215324193	-1.07758184910818
C	7.88851317674504	-3.65237982929370	-1.68824488008629
H	8.71607118513106	-3.98500925530312	-1.05792576675989
C	6.94214006082124	-3.51369612219408	-3.74583065173757
H	7.01103314251104	-3.76136481460229	-4.80586824559036
C	5.85098580966922	-2.80822656621451	-3.26238648648636
H	5.07563690093388	-2.49425674699093	-3.95708800087337
C	1.19870823366884	0.43806465564142	2.82059955505886
H	1.22410081168573	1.46277745244567	2.45624317969197
C	1.68667957867212	0.11637185796831	4.07924674817409
H	2.10202389294995	0.89686923240538	4.71199779254087
C	1.07071479571967	-2.15836334296210	3.64746290905317
H	1.00591774045477	-3.19572603007809	3.96206316792909
C	1.62968809437994	-1.21072240096513	4.49643749197243
H	2.00731646248040	-1.50404644892951	5.47390790046803
C	0.59025492214069	-1.76503413662509	2.39791978089859
C	-0.29149320802006	-4.03550772494429	1.72958497518858

H	0.05761409000749	-4.47450775791083	2.65937211503047
C	-0.97698713030923	-4.81826622663939	0.80839859203810
H	-1.16087861196124	-5.87139876513433	1.01179152283738
C	-1.43273698290840	-4.22927602509689	-0.36787871628835
H	-1.98762837223326	-4.79526732098916	-1.11239922969711
C	-1.16967945230035	-2.88342077084702	-0.58162392185388
H	-1.51319836994057	-2.38292008370894	-1.48429484410222
C	0.64696783105416	1.76531186233277	-0.12509562973253
C	-0.57659023602846	0.08597212740455	-1.85009342500413
C	-1.70955118863147	0.59947359223471	0.54652386175392
C	-0.06992654673007	-2.68535571204510	1.45617417020656

$S_1^{SSshort}$

Re	0.03135148527883	-0.05372999876913	-0.01366472519885
S	5.78184591263796	-0.34568322600157	0.30384134721049
O	1.11997667611827	2.83145179005596	-0.31324306544578
N	1.94414369387002	-0.95122800200960	-0.61357543399888
C	3.09094093014939	-0.52176470736583	-0.07085627160692
H	3.02802155911050	0.24601475666314	0.69695913900200
O	-0.82975874208681	0.41749587812354	-2.95276786105517
S	6.77824813067449	-2.14803389013921	0.60594470675505
N	8.11242671075197	-3.63900363035419	-2.85717422225837
C	4.33376421857798	-1.02892429688977	-0.43511930447117
O	-2.80097255072647	0.82662330173123	0.90825428014683
N	0.73019460041296	-0.48673612409930	1.94927621126469
C	4.41181474449837	-2.03313934674643	-1.42425593491560
N	-0.53723515359061	-2.09210343288504	0.23945649263780
C	3.19487532190164	-2.49520758469064	-1.93687147049902
H	3.17223898234957	-3.30417671898818	-2.66319437752454
C	1.99769623434047	-1.93736512163684	-1.52227430925836
H	1.05342197916718	-2.29835651073347	-1.92107858173738
C	5.69593039334252	-2.56322622800752	-1.91237295454647
C	6.83654740391033	-2.64597465483525	-1.08999900474003
C	8.01174959550647	-3.19589932352541	-1.61206174839949
H	8.90020458829700	-3.26727975127361	-0.98085442204845
C	7.05290254367072	-3.53569320127302	-3.65171678600269
H	7.17344537234195	-3.88234626070289	-4.67921616164600
C	5.83358747494885	-3.00712568514870	-3.23182812449611
H	5.01093101356924	-2.92240559695102	-3.93933049965019
C	1.30714537467752	0.39321873804680	2.79243933435512
H	1.36068560438713	1.42515462991543	2.44939481152845
C	1.80698793802349	0.04171970099499	4.02411025541586
H	2.25976761533488	0.79295953135295	4.66434278486624
C	1.10845983997008	-2.22278406127121	3.58060540334725
H	1.02050999318685	-3.26226940414071	3.88291979638360
C	1.70870335711333	-1.31510656870103	4.41776376699242
H	2.09895930294287	-1.63656048719526	5.38121256805665
C	0.59193667325468	-1.81160483704247	2.32610600661119
C	-0.35530911625556	-4.03960680734159	1.65125528317662

H	0.00137692924713	-4.50719263988608	2.56435843746159
C	-1.07418468808741	-4.77484200705519	0.74191012111362
H	-1.28123521245594	-5.82604695302502	0.93177066343690
C	-1.55737947694451	-4.15253704663214	-0.43581893113140
H	-2.14765727643343	-4.69401170680497	-1.16932760269315
C	-1.26376917633477	-2.82317302039925	-0.62948016925746
H	-1.61875785253252	-2.30206672323092	-1.51719763914493
C	0.71725068511295	1.76853994833803	-0.19428977383694
C	-0.52033578893769	0.23403735076696	-1.86778668785137
C	-1.75937638297302	0.51909293491289	0.57000398061924
C	-0.08852671531889	-2.66699239314937	1.41640616403310

S12 Other Possible Disregarded Mechanisms

The TRIR spectra in Figure 4b could be empirically interpreted in an alternative way: A hot ^{3}MLCT state ($T_1^{SSshort}$) is populated ultrafast within the instrument time resolution. The hot ^{3}MLCT state simultaneously relaxes (manifested by the dynamic blueshift) and decays (within ~20 ps) to the lower-lying ^{3}LC state. In this way, the internal conversion (^{3}MLCT → ^{3}LC) is coupled to relaxation and ceases when the system becomes fully relaxed/equilibrated internally as well as solvationally. ^{3}LC then decays to the GS with a comparable or faster rate, so that its steady-state concentration is low and does not give rise to a discernible IR signal.

The strongest argument against this mechanism is the experimental observation that the yield of equilibrated ^{3}MLCT population after 100 ps (when vibrational relaxation is over) is independent on the excitation wavelength. For both 400 and 266 nm excitation, the yield of the long-lived ^{3}MLCT population is about η=10%. This would not be possible if η depends on a competition between relaxation and reaction, because the excess energy at 266 nm is more than 2 times higher than at 400 nm excitation (see below).

The reaction of a non-thermal distribution competing against vibrational relaxation can be excluded also from a theoretical estimation. This scenario would require that the reaction rate constant k'_{IC} of a vibrationally hot $T_1^{SSshort}$ towards T_1^{SSlong} is faster than vibrational relaxation. In the following we estimate k'_{IC} by determining microcanonical rate constants $k(E)$ for the $T_1^{SSshort} \rightarrow T_1^{SSlong}$ reaction from RRKM theory,[42] and averaging these over the vibrational energy distribution $f(E)$ in the $T_1^{SSshort}$ state generated by photoexcitation and ISC according to

$$k'_{\mathrm{IC}} = \int k(E) f(E)\, dE \quad . \qquad \text{(S2)}$$

Before excitation a canonical energy distribution within the vibrational degrees of freedom of the S_0 state of the molecule exists. This distribution is given by

$$f(E)dE = \rho(E) \exp\left(-\frac{E}{k_B\, T}\right) dE \quad , \qquad \text{(S3)}$$

where $\rho(E)$ is the vibrational density of states of the S_0, k_B is the Boltzmann constant, and the temperature is $T = 298$ K. The density of states is calculated by direct harmonic state counting,[43] using the ground state vibrational frequencies of **1**$^+$ from DFT (including a frequency correction factor of 0.955). Laser excitation and subsequent ISC transfers this distribution from the ground to the $T_1^{SSshort}$ state. Accordingly, the vibrational energy distribution in the $T_1^{SSshort}$ state, corresponds approximately to the one in the ground state, however, shifted by the difference of the applied photon energy ($E_{\mathrm{ph}} = 25000\ \mathrm{cm}^{-1}$) and the energy of the $T_1^{SSshort}$ state ($E_{\mathrm{T1}} = 20000\ \mathrm{cm}^{-1}$, derived from the onset of the luminescence spectrum at 500 nm). The two vibrational distributions are shown in Figure S38. The average vibrational energies in the S_0 and $T_1^{SSshort}$ state are 5800 and 10800 cm^{-1}, respectively.

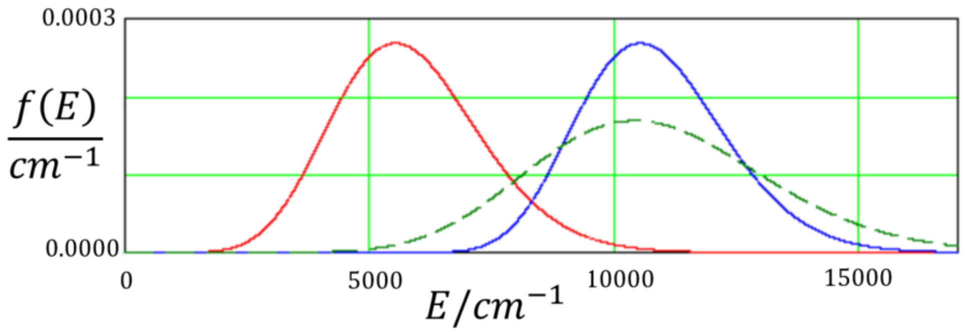

Figure S38: Vibrational energy distributions in the S_0 (red) and the $T_1^{SSshort}$ (blue) state after 400 nm excitation and ISC. The dashed curve corresponds to a thermal distribution at 410 K having the same average energy in the $T_1^{SSshort}$ state as the non-thermal blue distribution.

Microcanonical RRKM rate constants are calculated from the standard expression.[44]

$$k(E) = \frac{W^{\ddagger}(E-E_0)}{h\,\rho(E)} \quad . \tag{S4}$$

Here $\rho(E)$ is the vibrational density of states of the reactant (the $T_1^{SSshort}$ state), $W^{\ddagger}(E)$ is the vibrational sum of states of the transition state (TS), E_0 is the barrier height for the reaction, and h is Planck's constant. For the calculation of $\rho(E)$ we used the vibrational frequencies of the $T_1^{SSshort}$ state determined from DFT. Since the TS structure for the reaction could not be localized by DFT we employed the same set of frequencies also to compute $W^{\ddagger}(E)$ omitting, however, the reactive mode corresponding to the S-S stretching vibration at $512\ \mathrm{cm}^{-1}$. A lower limit for the barrier height was calculated from the measured photoluminescence lifetime ($\tau_{ph} = 270$ ns) assuming that τ_{ph} is determined by the $T_1^{SSshort} \rightarrow T_1^{SSlong}$ reaction. Applying Eq. (A) with a thermal $T_1^{SSshort}$ vibrational distribution at 298 K and a value of $E_0 = 2950\ \mathrm{cm}^{-1}$ reproduces a lifetime of $k'^{-1}_{IC} = 270$ ns. The microcanonical rate constants obtained with this E_0 value are shown in Figure S39 (red line).

Having fixed E_0, the lifetime for the vibrationally hot $T_1^{SSshort}$ state produced by 400 nm excitation and ISC can be calculated resulting in $k'^{-1}_{IC} = 6.3$ ns, i.e. the reaction is >2 orders of magnitude slower than the vibrational cooling process, thereby disproving the proposed mechanism.

Even for a vibrationally hot $T_1^{SSshort}$ distribution produced by 266 nm excitation (see dashed blue curve in Figure S39), the calculated lifetime of $k'^{-1}_{IC} = 104$ ps is too long to compete against vibrational cooling. This explains why the TRIR signals show a rather weak dependence on the pump wavelength (cf. Figure 4b and Figure S7).

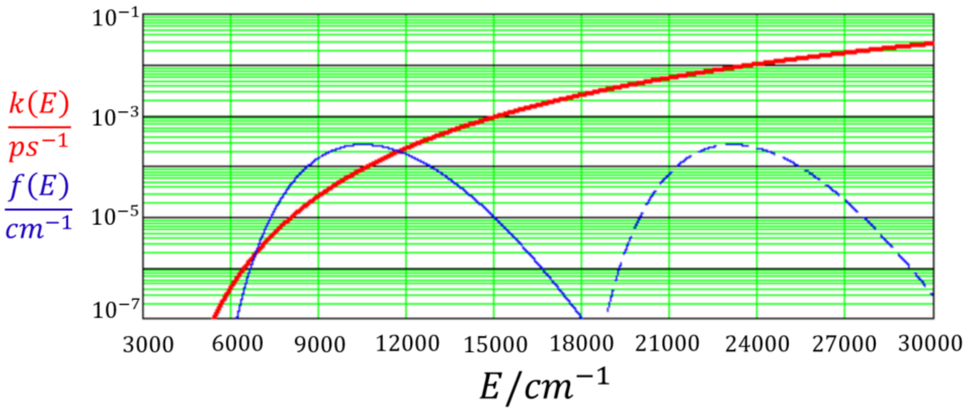

Figure S39: Microcanonical rate constants for the $T_1^{SSshort} \rightarrow T_1^{SSlong}$ reaction with a barrier height of $E_0 = 2950\ \mathrm{cm}^{-1}$ (red). The blue curves correspond to vibrational energy distributions in the $T_1^{SSshort}$ state produced by 400 (solid) and 266 nm (dashed) excitation.

S13 Crystallographic Details

Table S5. Crystal data and structure refinement for **[1]PF_6**.

Empirical formula	C24 H16 Cl2 F6 N4 O3 P Re S2	
Formula weight	874.60	
Temperature	133(2) K	
Wavelength	0.71073 Å	
Crystal system	Monoclinic	
Space group	$P2_1/n$	
Unit cell dimensions	a = 10.3402(2) Å	a= 90°.
	b = 19.4755(5) Å	b= 95.276(2)°.
	c = 14.6468(3) Å	g = 90°.
Volume	2937.08(11) $Å^3$	
Z	4	
Density (calculated)	1.978 Mg/m^3	
Absorption coefficient	4.590 mm^{-1}	
F(000)	1688	
Crystal size	0.500 x 0.400 x 0.380 mm^3	
Theta range for data collection	1.744 to 26.942°.	
Index ranges	-13<=h<=13, -24<=k<=24, -18<=l<=18	
Reflections collected	41724	
Independent reflections	6231 [R(int) = 0.0211]	
Completeness to theta = 25.242°	100.0 %	
Absorption correction	Numerical	
Max. and min. transmission	0.9552 and 0.8432	
Refinement method	Full-matrix least-squares on F^2	
Data / restraints / parameters	6231 / 0 / 388	
Goodness-of-fit on F^2	1.121	
Final R indices [I>2sigma(I)]	R1 = 0.0193, wR2 = 0.0475	
R indices (all data)	R1 = 0.0213, wR2 = 0.0482	
Extinction coefficient	n/a	
Largest diff. peak and hole	1.761 and -0.464 e.$Å^{-3}$	

S14 References

[1] S. Durben, T. Baumgartner, *Angew. Chem.* **2011**, *50*, 7948.
[2] E. Hevia, J. Pérez, L. Riera, V. Riera, I. del Río, S. García-Granda, D. Miguel, *Chem. Eur. J.* **2002**, *8*, 4510.
[3] a) F. Neese, *Wiley Interdiscip. Rev. Comput. Mol. Sci.* **2012**, *2*, 73; b) F. Neese, *Wiley Interdiscip. Rev. Comput. Mol. Sci.* **2018**, *8*.
[4] C. Adamo, V. Barone, *J. Chem. Phys.* **1999**, *110*, 6158.
[5] D. A. Pantazis, X.-Y. Chen, C. R. Landis, F. Neese, *J. Chem. Theory Comput.* **2008**, *4*, 908.
[6] F. Weigend, R. Ahlrichs, *Phys. Chem. Chem. Phys.* **2005**, *7*, 3297.
[7] E. Caldeweyher, S. Ehlert, A. Hansen, H. Neugebauer, S. Spicher, C. Bannwarth, S. Grimme, *J. Chem. Phys.* **2019**, *150*, 154122.
[8] F. Neese, F. Wennmohs, A. Hansen, U. Becker, *Chem. Phys.* **2009**, *356*, 98.
[9] E. van Lenthe, J. G. Snijders, E. J. Baerends, *J. Chem. Phys.* **1996**, *105*, 6505.
[10] P. J. Stephens, F. J. Devlin, C. F. Chabalowski, M. J. Frisch, *J. Phys. Chem.* **1994**, *98*, 11623.
[11] T. Yanai, D. P. Tew, N. C. Handy, *Chem. Phys. Lett.* **2004**, *393*, 51.
[12] Y. Tawada, T. Tsuneda, S. Yanagisawa, T. Yanai, K. Hirao, *J. Chem. Phys.* **2004**, *120*, 8425.
[13] Y. Zhao, D. G. Truhlar, *Theor Chem Account* **2008**, *120*, 215.
[14] O. S. Bokareva, G. Grell, S. I. Bokarev, O. Kühn, *J. Chem. Theory Comput.* **2015**, *11*, 1700.
[15] S. Hirata, M. Head-Gordon, *Chem. Phys. Lett.* **1999**, *314*, 291.
[16] L. Sun, W. L. Hase, *J. Chem. Phys.* **2010**, *133*, 44313.
[17] J. P. Zobel, J. J. Nogueira, L. González, *Phys. Chem. Chem. Phys.* **2019**, *21*, 13906.
[18] A. V. Marenich, C. J. Cramer, D. G. Truhlar, *J. Phys. Chem. B* **2009**, *113*, 6378.
[19] F. Plasser, *J. Chem. Phys.* **2020**, *152*, 84108.
[20] S. Mai, F. Plasser, J. Dorn, M. Fumanal, C. Daniel, L. González, *Coord. Chem. Rev.* **2018**, *361*, 74.
[21] J. C. Tully, *J. Chem. Phys.* **1990**, *93*, 1061.
[22] a) S. Mai, M. Richter, M. Heindl, Menger, M. F. S. J., A. Atkins, M. Ruckenbauer, F. Plasser, L. M. Ibele, S. Kropf, M. Oppel et al., *SHARC2.1: Surface Hopping Including Arbitrary Couplings – Program Package for Non-Adiabatic Dynamics*, **2019**; b) S. Mai, P. Marquetand, L. González, *Wiley Interdiscip. Rev. Comput. Mol. Sci.* **2018**, *8*, e1370; c) M. Richter, P. Marquetand, J. González-Vázquez, I. Sola, L. González, *J. Chem. Theory Comput.* **2011**, *7*, 1253.
[23] M. Barbatti, M. Ruckenbauer, F. Plasser, J. Pittner, G. Granucci, M. Persico, H. Lischka, *Wiley Interdiscip. Rev. Comput. Mol. Sci.* **2014**, *4*, 26.
[24] a) M. Heindl, J. Hongyan, S.-A. Hua, M. Oelschlegel, F. Meyer, D. Schwarzer, L. González, *Inorg. Chem.* **2021**, *60*, 1672; b) S.-A. Hua, M. Cattaneo, M. Oelschlegel, M. Heindl, L. Schmid, S. Dechert, O. S. Wenger, I. Siewert, L. González, F. Meyer, *Inorg. Chem.* **2020**, *59*, 4972.
[25] G. Granucci, M. Persico, A. Zoccante, *J. Chem. Phys.* **2010**, *133*, 134111.
[26] F. Plasser, M. Ruckenbauer, S. Mai, M. Oppel, P. Marquetand, L. González, *J. Chem. Theory Comput.* **2016**, *12*, 1207.
[27] A. Dreuw, M. Head-Gordon, *Chem. Rev.* **2005**, *105*, 4009.
[28] B. O. Roos in *Challenges and Advances In Computational Chemistry and Physics* (Eds.: J. Leszczynski, M. K. Shukla), Springer Netherlands, Dordrecht, **2008**, pp. 125–156.
[29] G. Ghigo, B. O. Roos, P.-Å. Malmqvist, *Chem. Phys. Lett.* **2004**, *396*, 142.
[30] J. P. Zobel, J. J. Nogueira, L. González, *Chem. Sci.* **2017**, *8*, 1482.
[31] a) B. O. Roos, P. R. Taylor, P. E. Sigbahn, *Chem. Phys.* **1980**, *48*, 157; b) K. Andersson, P.-Å. Malmqvist, B. O. Roos, *J. Chem. Phys.* **1992**, *96*, 1218.
[32] I. Fdez Galván, M. Vacher, A. Alavi, C. Angeli, F. Aquilante, J. Autschbach, J. J. Bao, S. I. Bokarev, N. A. Bogdanov, R. K. Carlson et al., *J. Chem. Theory Comput.* **2019**, *15*, 5925.
[33] B. O. Roos, R. Lindh, P.-Å. Malmqvist, V. Veryazov, P.-O. Widmark, *J. Phys. Chem. A* **2004**, *108*, 2851.

[34] F. Aquilante, R. Lindh, T. B. Pedersen, *J. Chem. Phys.* **2007**, *127*, 114107.
[35] N. Forsberg, P.-Å. Malmqvist, *Chem. Phys. Lett.* **1997**, *274*, 196.
[36] K. Andersson, B. O. Roos, *Int. J. Quantum Chem.* **1993**, *45*, 591.
[37] H. Köuppel, W. Domcke, L. S. Cederbaum, *Advances in Chemical Physics* (Eds.: I. Prigogine, S. A. Rice), Wiley, **1984**, pp. 59–246.
[38] L. H. Jones, R. S. McDowell, M. Goldblatt, *Inorg. Chem.* **1969**, *8*, 2349.
[39] M. Fumanal, F. Plasser, S. Mai, C. Daniel, E. Gindensperger, *J. Chem. Phys.* **2018**, *148*, 124119.
[40] J. P. Zobel, O. S. Bokareva, P. Zimmer, C. Wölper, M. Bauer, L. González, *Inorg. Chem.* **2020**, *59*, 14666.
[41] J. P. Zobel, L. González, *JACS Au* **2021**, *1*, 1116.
[42] A. Meyer, J. Schroeder, J. Troe, M. Votsmeier, *Journal of Photochemistry and Photobiology A: Chemistry* **1997**, *105*, 345.
[43] T. Beyer, D. F. Swinehart, *Commun. ACM* **1973**, *16*, 379.
[44] a) K. A. Holbrook, M. J. Pilling, S. H. Robertson, *Unimolecular reactions*, Wiley, Chichester, **1996**; b) J. I. Steinfeld, J. S. Francisco, W. L. Hase, *Chemical kinetics and dynamics*, Prentice Hall, Upper Saddle River, NJ, **1999**; c) R.G. Gilbert, S. C. Smith, *Theory of unimolecular and recombination reactions*, Blackwell Scientific Publ, Oxford, **1990**.
